# Synthesis of chiral 1,4-oxazepane-5-carboxylic acids from polymer-supported homoserine†

**DOI:** 10.1039/d0ra07997a

**Published:** 2020-09-30

**Authors:** Petra Králová, Barbora Lemrová, Michal Maloň, Miroslav Soural

**Affiliations:** Department of Organic Chemistry, Faculty of Science, Palacký University 771 46 Olomouc Czech Republic miroslav.soural@upol.cz; JEOL (U.K.) Ltd. JEOL House, Silver Court, Watchmead Welwyn Garden City Hertfordshire AL7 1LT UK; Institute of Molecular and Translational Medicine, Faculty of Medicine and Dentistry, Palacký University Hněvotínská 5 779 00 Olomouc Czech Republic

## Abstract

The preparation of novel 1,4-oxazepane-5-carboxylic acids bearing two stereocenters is reported in this article. Fmoc-HSe(TBDMS)-OH immobilized on Wang resin was reacted with different nitrobenzenesulfonyl chlorides and alkylated with 2-bromoacetophenones to yield *N*-phenacyl nitrobenzenesulfonamides. Their cleavage from the polymer support using trifluoroacetic acid (TFA) led to the removal of the silyl protective group followed by spontaneous lactonization. In contrast, TFA/triethylsilane (Et_3_SiH)-mediated cleavage yielded 1,4-oxazepane derivatives as a mixture of inseparable diastereomers. The regioselectivity/stereoselectivity depended on the substitution of the starting 2-bromoacetophenones and was studied in detail. Catalytic hydrogenation of the nitro group improved the separability of the resulting diastereomeric anilines, which allowed us to isolate and fully characterize the major isomers.

## Abstract 2

The preparation of novel 1,4-oxazepane-5-carboxylic acids bearing two stereocenters is reported in this article.

## Graphical abstract

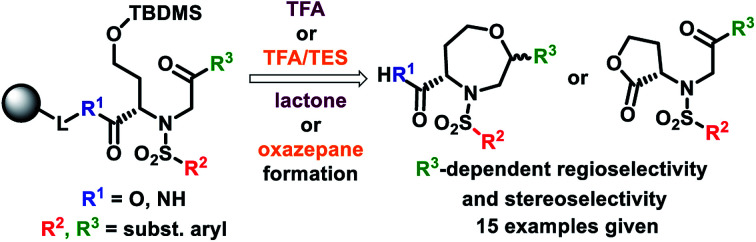

## Introduction

**Fig. 1:**
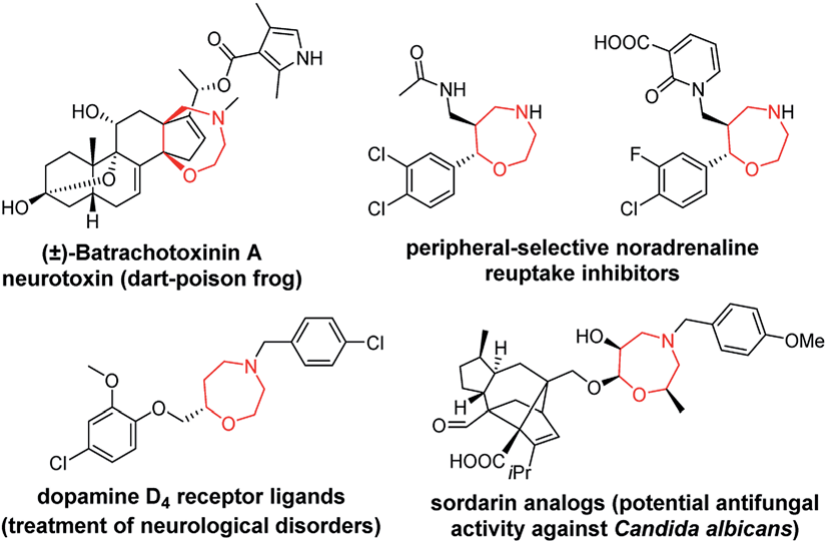
Selected pharmacologically relevant compounds bearing a 1,4-oxazepane scaffold.^1–6^

Chiral seven-membered heterocycles bearing one or more heteroatoms in their skeleton are an interesting group of compounds with unique physico-chemical and biological properties. The prominent heterocyclic scaffold within this group is represented by 1,4-oxazepanes, which occur in both synthetic compounds and natural products (Fig. 1).^1–6^

Compounds bearing 1,4-oxazepane scaffolds have been reported as potent anticonvulsants^7^ and antifungal agents^5,8^ or agents to treat inflammatory bowel disease,^9^ lupus nephritis^10^ and respiratory diseases, including asthma and bronchiectasis.^11,12^ Over the past decade, synthetic chemists have struggled to develop different strategies to access 1,4-oxazepanes from various starting materials.

The most robust synthetic approaches reported to date are based on intramolecular cyclization of alkenols,^13,14^ alkynols^15,16^ or hydroxyketones,^16^ typically using Brönsted or Lewis acids; however, some alternative methods of limited applicability have also been described recently.^17–25^

**Fig. 2:**
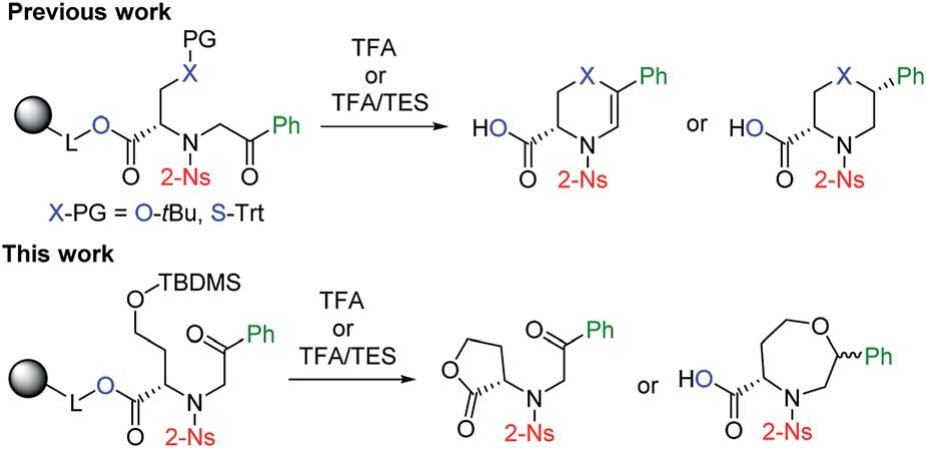
Previously reported stereoselective synthesis of chiral (thio)morpholines and the application of the method to homological starting material.

Although the synthetic availability of chiral 1,4-oxazepanes has already been determined, the decoration of the scaffold with reactive functional groups amenable to further diversification remains a challenging task due to the limited applicability of previously developed procedures for functionalizing starting materials. In our previous contribution, we reported the simple synthesis of chiral morpholines starting from resin-bound serine.^26^ Using either TFA- or TFA/Et_3_SiH-induced cleavage of the corresponding polymer-supported intermediates (Fig. 2), we synthesized either dihydrooxazine-3-carboxylic acids or morpholine-3-carboxylic acids with full control of the newly formed stereocenter. Later, we extended this method to the simple synthesis of fused [6 + 7]^27,28^ or [6 + 6]^29,30^ morpholines. To eventually synthesize the corresponding homological compounds, we decided to use polymer-supported homoserine to access the 1,4-oxazepane-5-carboxylic acids suitable for further modification. In this article, we report on the applicability, regioselectivity and stereoselectivity of the proposed method.

## Results and discussion

**Scheme 1:**
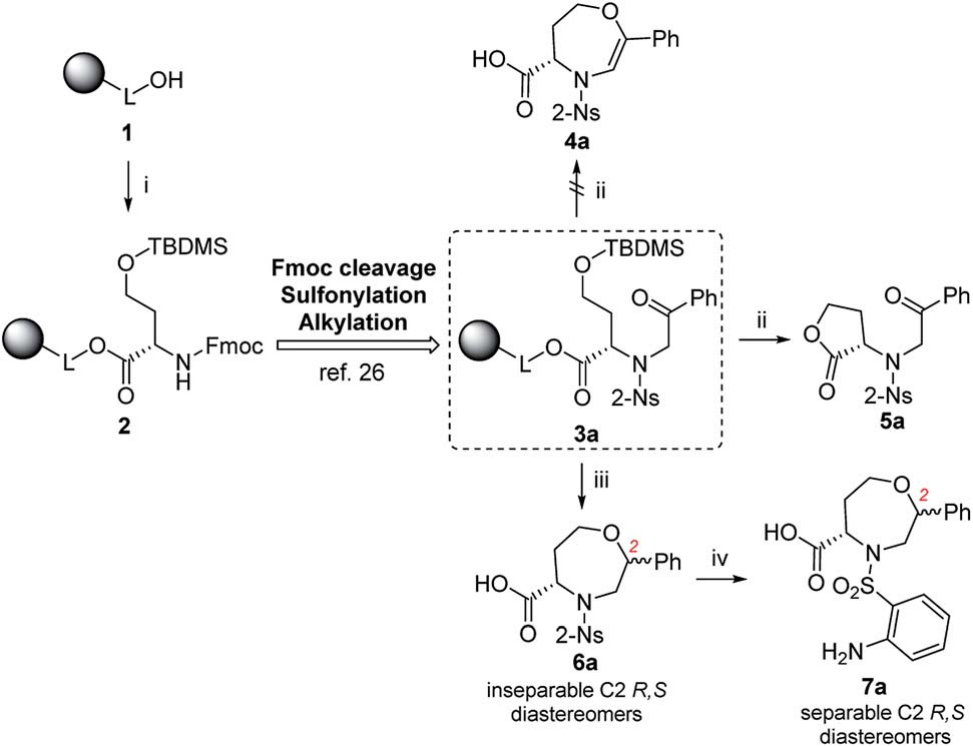
The reactivity of resin-bound intermediate 3a under different reaction conditions. Reagents and conditions: (i) 1-hydroxybenzotriazole (HOBt), 4-(dimethylamino)pyridine (DMAP), diisopropylcarbodiimide (DIC), *N*,*N*-dimethylformamide (DMF), CH_2_Cl_2_, 24 h, rt; (ii) 50% trifluoroacetic acid (TFA)/CH_2_Cl_2_, 1 h, rt; (iii) TFA/triethylsilane (Et_3_SiH)/CH_2_Cl_2_ (10 : 1 : 9), 30 min, rt; (iv) H_2_, 10% palladium on carbon (Pd/C) or platinum(iv) oxide (PtO_2_), 2-isopropanol (IPA), 24 h, rt.

Fmoc-HSe(TBDMS)-OH was prepared from homoserine in two steps^31,32^ and immobilized on Wang resin using the 1-hydroxybenzotriazole (HOBt)/diisopropylcarbodiimide (DIC) technique to suppress racemization. The key intermediate 3a was synthesized according to our previous protocols^26^ consisting of Fmoc-protective group cleavage, reaction with 2-nitrobenzenesulfonyl chloride (2-Ns-Cl) and alkylation using 2-bromoacetophenone (Scheme 1). Inspired by the smooth cyclization of serine-based analogs to 3,4-dihydro-1,4-oxazine-3-carboxylic acids,^26^ we hypothesized that exposure of resin 3a to TFA could yield the corresponding homologous product, *i.e.*, 4,5,6,7-tetrahydro-1,4-oxazepine-5-carboxylic acid 4a. The reaction yielded a single product with 87% crude purity (calculated from HPLC-UV traces at 205–400 nm) and 74% overall yield (calculated from the ^1^H NMR spectrum of the purified product). Although HRMS analysis corresponded to the molecular mass of suggested product 4a, NMR analysis (see ESI for details†) revealed preferential lactonization, which yielded compound 5a. Interestingly, when Et_3_SiH was added to the cleavage cocktail, a different course of reaction was observed. We received two chromatographically inseparable compounds with identical molecular masses (as indicated by HPLC-UV-MS analysis, the combined crude purity was 85%). To eventually improve the separability, we performed catalytic hydrogenation using palladium on carbon (Pd/C) in 2-isopropanol (IPA),^28^ which again afforded a mixture of two isomers (the combined crude purity was 91%, and the ratio was 56 : 44, as calculated from HPLC-UV traces at 205–400 nm); however, in this case, reverse-phase chromatography (RP-HPLC) indicated possible separation. Consequently, the major isomer was successfully isolated using semipreparative RP-HPLC at 34% overall yield (calculated from the ^1^H NMR spectrum of the purified product) and subjected to detailed NMR investigation.

**Fig. 3:**
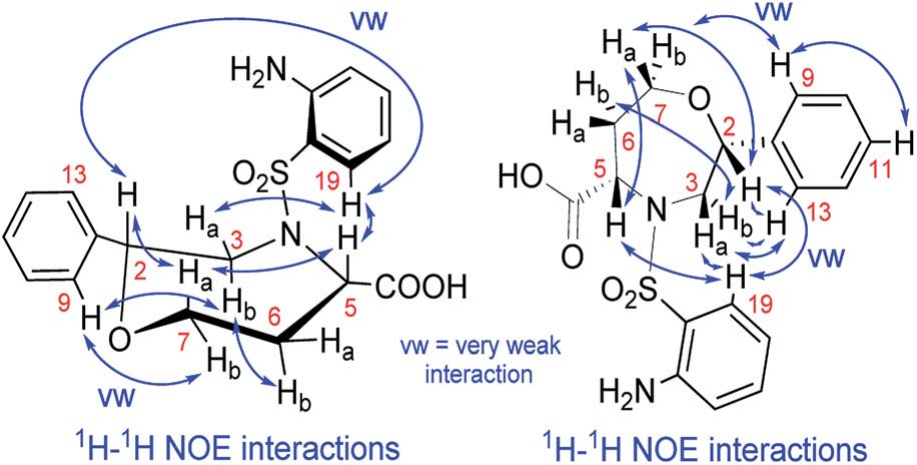
The NOE correlations prove the constitution and configuration of oxazepane derivative 7a.

We recorded and analyzed ^1^H, ^13^C{^1^H}, APT, ^1^H–^1^H COSY, ^1^H–^1^H NOESY, ^1^H–^13^C HMQC, ^1^H–^13^C HMBC and ^1^H–^15^N HMBC NMR data to determine the constitution. Complete assignment of the ^1^H, ^13^C and ^15^N signals was possible and is shown in the ESI (Fig. S17–S19 and Table S2†). In brief, by means of the homonuclear and heteronuclear correlation data, we identified the 1,4-oxazepane-5-carboxylic acid, phenyl and 2-aminobenzenesulfonyl moieties indicating the structure of 7a. The connectivity between the oxazepane and phenyl rings was confirmed by three long-range ^1^H–^13^C correlations (see ESI, Fig. S18†). Finally, the planar structure was established by the ^1^H–^1^H NOESY spectrum, which gave the key correlations between oxazepane protons and aminobenzenesulfonyl proton H^19^ and correlations between oxazepane protons and phenyl protons H^9,13^ (Fig. 3 and S19†). All the 1D and 2D NMR spectra can be found in the ESI (Fig. S20–S27†).

To determine the conformation and relative configuration of 7a, we analyzed the ^1^H–^1^H coupling constants on the oxazepane ring and the NOE correlations in detail. Despite the spatial flexibility of seven membered rings leading to multiple possible conformational states, the analysis of vicinal ^1^H–^1^H couplings indicated that the scaffold existed in the most energetically favourable chair conformation (Fig. 3). Since the configuration of the C5 stereocenter was defined by the configuration of the starting material (*S*), the configuration of the newly formed C2 stereocenter was assigned as *R*.

**Fig. 4:**
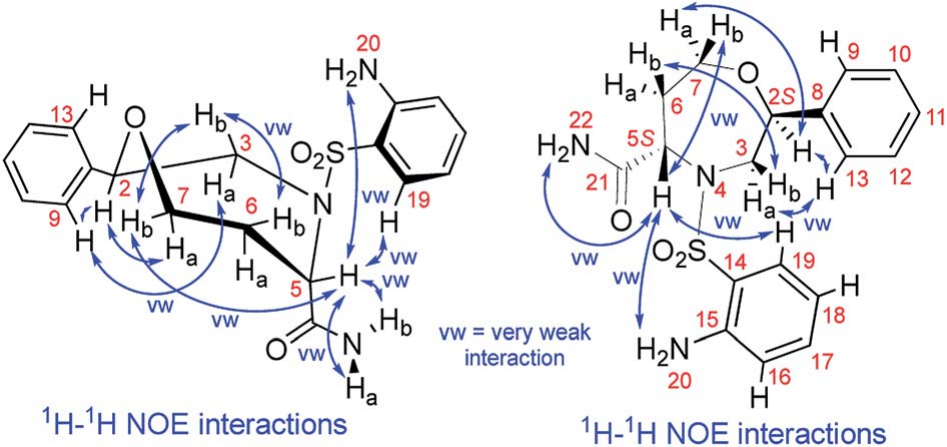
The NOE correlations proving the configuration of oxazepane derivative 7b^2^*^S^*.

Although we did not isolate and analyze the minor isomer of 7a, in the case of 7b, RP-HPLC purification enabled the separation and isolation of both isomers. Thorough NMR structural analysis indicated the formation of C2 *R*,*S* diastereomers 7b^2^*^R^* and 7b^2^*^S^* (Table 1). The ^1^H NMR spectrum of 7b^2^*^R^* was fairly similar to the spectrum of 7a; hence, we concluded that this compound had the same configuration as 7a. Compound 7b^2^*^S^* was analyzed by 1D and 2D NMR spectroscopy (see ESI, Fig. S34–S41 and Table S3†) to verify the planar structure, assign all the ^1^H and ^13^C signals and determine the relative configuration. Finally, we confirmed the configuration of 7b^2^*^S^* by analyzing the vicinal ^1^H–^1^H couplings on the oxazepane ring and NOE correlations (Fig. 4).

**Table 1:** Comparison of ^1^H chemical shifts, splitting patterns, and ^2^*J* and ^3^*J* homonuclear couplings for derivatives 7b^2^*^R^* and 7b^2^*^S^*

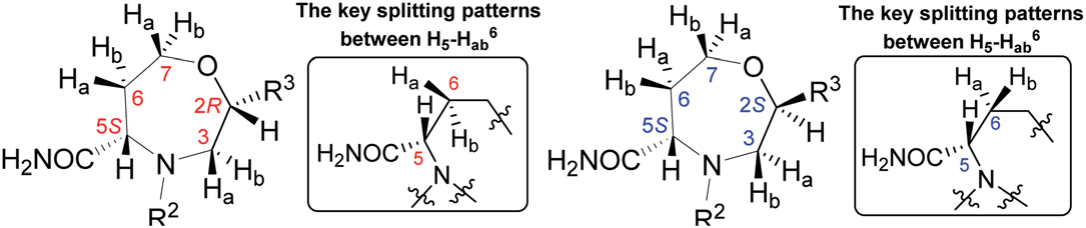						
Position	C2 *R* isomer			C2 *S* isomer		
	^1^H NMR *δ*_H_ [ppm]	Splitting pattern	*J* [Hz]	^1^H NMR *δ*_H_ [ppm]	Splitting pattern	*J* [Hz]
H^2^	4.28	dd	9.3, 1.2	4.64	dd	8.7, 1.6
H_a_^3^	3.82	ddd	16.2, 1.2, 1.2	3.63	dd	14.2, 1.6
H_b_^3^	3.53	dd	16.2, 9.6	3.43	dd	14.2, 8.7
H^5^	4.49	ddd	10.8, 7.1, 1.0	4.59	dd	4.5, 4.5
H_a_^6^	2.46	dddd	15.7, 7.1, 6.3, 1.0	2.30	dddd	15.8, 4.5, 4.5, 1.6
H_b_^6^	2.21	dddd	15.7, 10.8, 9.3, 1.5	2.10	dddd	15.8, 11.0, 4.5, 3.0
H_a_^7^	3.67	ddd	12.9, 9.3, 1.0	3.78	ddd	12.8, 11.0, 1.6
H_b_^7^	4.05	ddd	12.9, 6.3, 1.5	4.02	ddd	12.8, 4.5, 3.0

Table 1 summarizes the ^1^H chemical shifts, splitting patterns, and ^2^*J* and ^3^*J* homonuclear couplings observed in 7b^2^*^R^* and 7b^2^*^S^*. While the chemical shifts differ rather insignificantly between the two diastereomers, the splitting pattern of signal H^5^ and ^3^*J*(H^5^–H_a_^6^) and ^3^*J*(H^5^–H_b_^6^) can be used to differentiate between 7b^2^*^R^* and 7b^2^*^S^*. In the C2 *R* isomer 7b^2^*^R^*, two relatively large couplings and one very small long-range coupling leading to a doublet of doublet of doublets are observed. However, in the case of the C2 *S* isomer 7b^2^*^S^*, the vicinal coupling constants are smaller and equal or nearly equal, and hence, the signal takes the shape of a pseudotriplet (dd). The same trend was observed in all derivatives 6–7.

After proving the structure and formation of the two diastereomers, we tried to improve the stereoselectivity by using a lower reaction temperature (0 °C or −20 °C); however, a nearly equal ratio of isomers was obtained. Furthermore, we tested the use of TMSOTf/Et_3_SiH as reported earlier for an analogical starting material,^17^ but a complex mixture of unknown compounds was obtained.

**Scheme 2:**
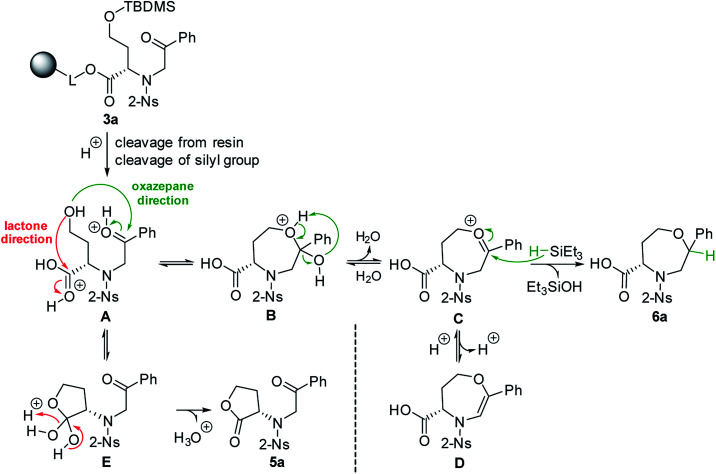
The hypothetical mechanism explaining the different reactivity of intermediate 3a.

To explain the different reaction outcomes depending on the composition of the cleavage cocktail, we suggested a reaction mechanism (Scheme 2). The protonation of intermediate 3a cleaved from the resin can be followed by intramolecular attack of ketone or carboxylic acid with the hydroxy group as the internal nucleophile. With respect to the higher electrophilicity of the ketone, we presume that the formation of intermediate B over E is preferred. Intermediate B can be further stabilized by the formation of intermediates C and D; however, due to their limited stability, all the reactions in pathway A–D are reversible, which can lead to regeneration of starting material A. The same is true for the formation of E from A. In contrast, the conversion of E to 5a is irreversible, as lactone does not undergo hydrolysis under the conditions used. In the presence of Et_3_SiH, the preferential formation of B is considered again; however, the subsequently formed intermediates C and D are attacked by triethylsilane as the external nucleophile, which leads to the formation of stable compound 6a. Consequently, the formation of lactone 5a was not observed in this case.

**Fig. 5:**
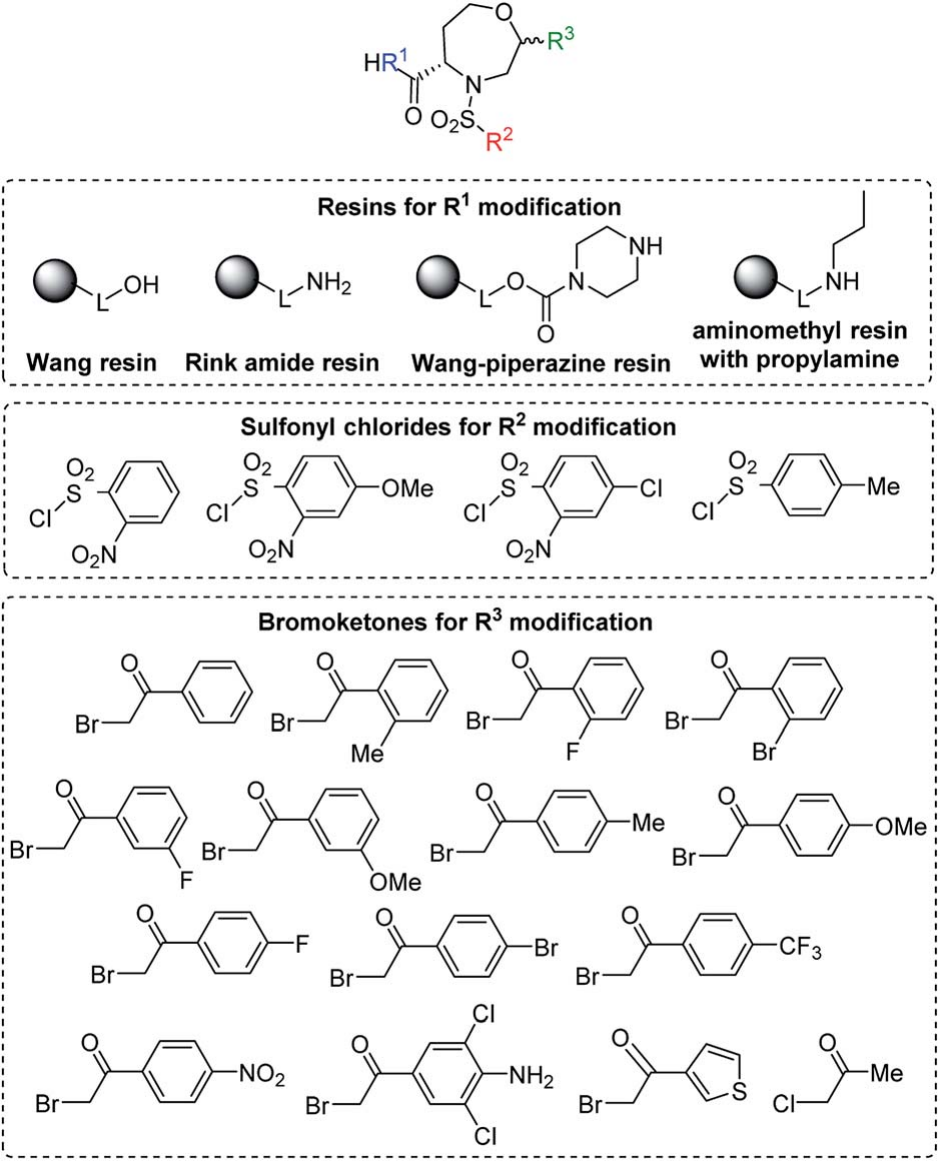
General structure of target compounds and the list of the tested starting synthons (see Table 2 for the list of target compounds).

After the determination of the reaction outcome, we used different starting materials to evaluate the limitations and scope of the methodology and to reveal the structure–regioselectivity and structure–stereoselectivity relationships. For this purpose, Wang resin was replaced with Rink amide resin, Wang-piperazine resin and BAL resin with immobilized propylamine^26^ to alter the carboxylic group to carboxamides. Furthermore, variously substituted sulfonyl chlorides and fourteen 2-bromoacetophenones bearing electron-donating or electron-withdrawing groups were selected, including one heterocyclic derivative (thienyl) and another aliphatic derivative (Me; Fig. 5).

First, we tested the combination of 2-Ns-Cl and 2-bromoacetophenone with different resins. The use of Rink amide resin (R^1^ = NH_2_) required repetition of the alkylation step and yielded the final aniline 7b in 89% crude purity as a mixture of C2 *R*,*S* diastereomers in a ratio of 45 : 55. Its RP-HPLC enabled the separation of both isomers in 9% (C2 *R* isomer) and 4% overall yields (C2 *S* isomer, Table 2). In the case of the Wang-piperazine resin (R^1^ = piperazin-1-yl), the TFA/Et_3_SiH-mediated cleavage of the corresponding sulfonamide from the resin unexpectedly caused the cleavage of the piperazine moiety, and oxazepane-5-carboxylic acid derivative 7a was obtained as a mixture of C2 *R*,*S* isomers in a ratio of 72 : 28. The major diastereomer was isolated in 10% overall yield. The use of BAL resin-immobilized propylamine failed in the alkylation step stage, which is in accordance with our previous results.^26^

**Table 2:** The list of synthesized and fully characterized compounds^a^^,^^b^^,^^c^^,^^d^^,^^e^^,^^f^

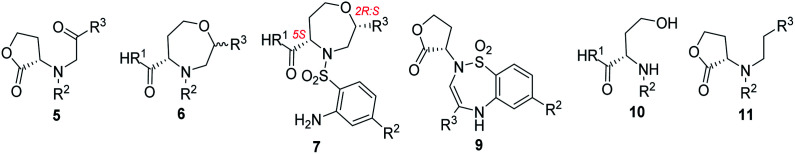							
Cmpd	R^1^	R^2^	R^3^	Crude diastereomeric ratio of C2 *R* : *S* stereoisomers^a^ [%]	Crude combined purity^b^ [%]	Final purity of major purified isomer^c^^,^^d^ [%]	Overall yield of major isomer^e^ [%]
5a	—	2-Ns	Ph	—	87	99^c^	74
6g	O	4-Me-Ph	Ph	62 : 38	73	97^d^	18
7a	O	H	Ph	56 : 44	91	98^d^	34
7a^f^	O	H	Ph	72 : 28	76	98^d^	10
7b^2^*^R^*	NH	H	Ph	45 : 55	89	100^d^	9
7b^2^*^S^*						100^d^	4
7e	O	MeO	Ph	69 : 31	91	100^d^	21
7f^2^*^RS^*	O	Cl	Ph	70 : 30	86	78^d^	20
7i^2^*^R^*	O	H	2-F-Ph	29 : 71	69	100^d^	4
7i^2^*^S^*						93^d^	10
7k	O	H	3-F-Ph	64 : 36	70	100^d^	10
7m	O	H	4-Me-Ph	77 : 23	90	100^d^	19
7o	O	H	4-F-Ph	67 : 33	77	94^d^	10
7q	O	H	4-CF_3_-Ph	61 : 39	33	96^d^	8
7t	O	H	3-Thienyl	93 : 7	89	98^d^	22
9h	—	H	2-Me-Ph	—	50	99^c^	19
9j	—	H	2-Br-Ph	—	49	99^c^	13
10r	O	2-Ns	—	—	82	99^c^	50
11n	—	2-Ns	4-MeO-Ph	—	77	99^c^	33

a Ratio of diastereomers prior to RP HPLC purification calculated from HPLC-UV traces at 205–400 nm.

b Combined crude purity of diastereomers after the entire reaction sequence calculated from HPLC-UV traces at 205–400 nm.

c Calculated from HPLC-UV traces at 205–400 nm after RP-HPLC purification.

d Ratio of C2 *R*,*S* diastereomers calculated from ^1^H NMR of the purified product.

e Calculated from the ^1^H NMR spectrum of the purified product.

f Product prepared from Wang-piperazine resin.

**Scheme 3:**
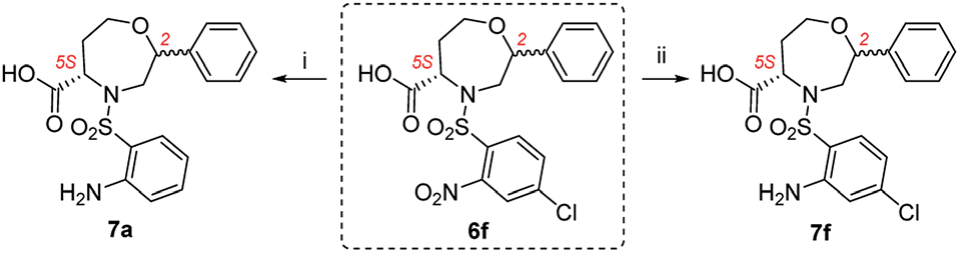
Dehalogenation of oxazepane derivative 6f during hydrogenation. Reagents and conditions: (i) H_2_, Pd/C, IPA, 24 h, rt; (ii) H_2_, PtO_2_, IPA, 24 h, rt.

After that, various sulfonylating agents (Fig. 5) were tested, starting from intermediate 2 and using 2-bromoacetophenone as the alkylating agent. In the case of sulfonamides 3e and 3g bearing 4-methoxy-2-nitrobenzenesulfonyl group or tosyl group as R^2^, the alkylation required a longer reaction time (2 and 6 days, respectively) to completion. The TFA/Et_3_SiH cleavage of tosyl intermediate 3g yielded the desired oxazepane in 73% crude purity as a partially separable mixture of C2 *R*,*S* isomers in a ratio of 62 : 38. Its RP-HPLC purification yielded the major isomer C2 *R* in 18% overall yield. In the case of 3e and 3f bearing 4-methoxy and 4-chloro-2-nitrobenzene-sulfonyl as R^2^ substituents, the nitro-oxazepane derivatives 6e and 6f were obtained as inseparable mixtures of C2 *R*,*S* diastereoisomers in 81–90% crude purities. Similar to previously reported results,^28^ the hydrogenation of 6f led to undesired hydrogenolysis of the C–Cl bond (Scheme 3). For this reason, Pd/C was replaced with PtO_2_, which yielded aniline 7f in 86% crude purity as a mixture of C2 *R*,*S* diastereomers in a ratio of 70 : 30. In this case, the isolation of the major isomer from the diastereomeric mixture was problematic and furnished the product C2 *R* in only 78% diastereomeric purity. In the case of 6e bearing a 4-methoxy group, hydrogenation using PtO_2_ suppressed the previously reported demethylation,^28^ and the final compound 7e was obtained in 91% crude purity as a mixture of C2 *R*,*S* diastereomers in a ratio of 69 : 31. RP-HPLC purification enabled the separation of the major C2 *R* isomer in 21% overall yield.

**Scheme 4:**
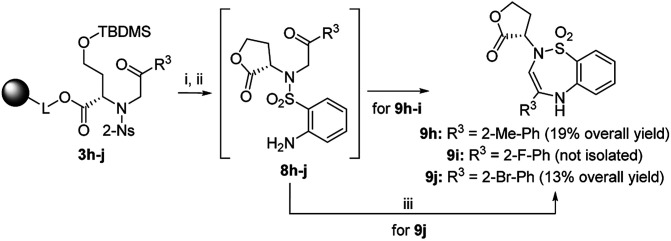
Preferential lactonization and formation of benzothiadiazepine 1,1-dioxide 9. Reagents and conditions: (i) TFA/Et_3_SiH/CH_2_Cl_2_ (10 : 1 : 9), 30 min, rt; (ii) H_2_, 10% Pd/C (for derivatives 9h–i) or PtO_2_ (for derivative 9j), IPA, 24 h, rt; (iii) 5% TFA/CH_2_Cl_2_, 24 h, rt.

Finally, we tested various 2-haloketones bearing electron-donating and electron-withdrawing groups in the *o*-, *m*- and *p*-positions, and one heterocyclic and aliphatic derivative was included. All these building blocks were tested in combination with intermediate 2 and 2-Ns-Cl. In the case of 7j and 7p bearing 2-Br-Ph or 4-Br-Ph as R^3^, PtO_2_ had to be used to avoid undesired debromination. The preferential formation of oxazepane was observed in each case. In the case of 7p, inseparable C2 *R*,*S* diastereomers were isolated in 14% overall yield as diastereomeric anilines in a ratio of 42 : 58. In the case of 3i bearing 2-F-Ph as R^3^, the regioselectivity was compromised, and the TFA/Et_3_SiH reaction yielded 1,4-oxazepane 6i accompanied by lactone 5i (26% according to HPLC). The following hydrogenation of the reaction mixture yielded the corresponding diastereomeric aniline 7i (69% combined crude purity) and amino-lactone 8i, which spontaneously cyclized to benzothiadiazepine 1,1-dioxide 9i (26% according to HPLC-UV-MS analysis) (Scheme 4).^33^ In this case, RP-HPLC purification enabled the separation of both diastereomers. Intermediates 3h (R^3^ = 2-Me-Ph) and 3j (R^3^ = 2-Br-Ph) yielded lactones 5h and 5j as the major products (72% and 64% crude purities). Their hydrogenation yielded the corresponding anilines 8h and 8j, which were cyclized to benzothiadiazepine 1,1-dioxides 9h and 9j.^33^ Derivatives 9h and 9j were isolated using semipreparative RP-HPLC at 19% overall yield and fully characterized. Interestingly, in contrast to previously reported results,^33^ compounds 9 were fully stable and did not undergo ring contraction to benzothiadiazine 1,1-dioxides.

**Scheme 5:**
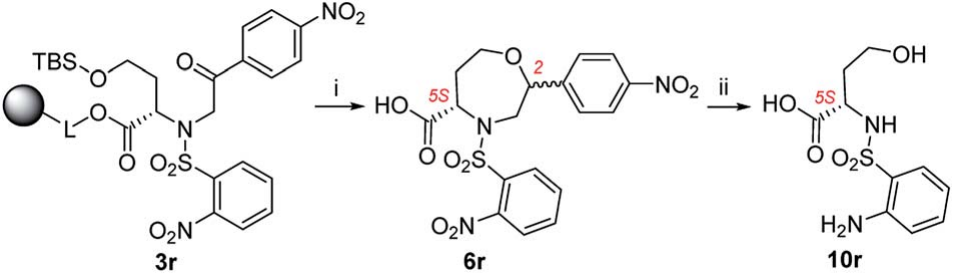
Cleavage of oxazepane derivative 6r during hydrogenation. Reagents and conditions: (i) TFA/Et_3_SiH/CH_2_Cl_2_ (10 : 1 : 9), 30 min, rt; (ii) H_2_, Pd/C, IPA, 24 h, rt.

**Scheme 6:**
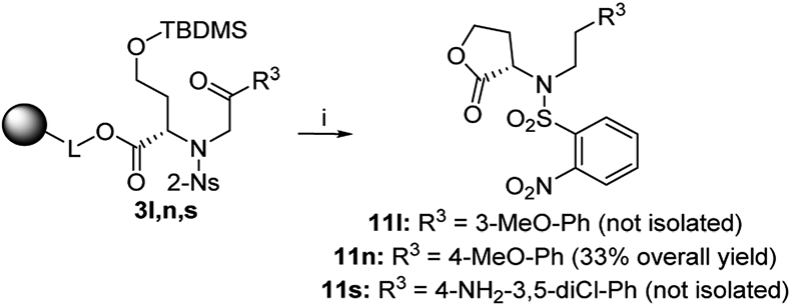
Preferential lactonization followed by ketone reduction to products 11. Reagents and conditions: (i) TFA/Et_3_SiH/CH_2_Cl_2_ (10 : 1 : 9), 24 h, rt.

In the case of 3k (3-F-Ph as R^3^), the TFA/Et_3_SiH reaction and the subsequent hydrogenation yielded diastereomeric aniline 7k in a ratio of 64 : 36 and 70% combined crude purity, and the major isomer was isolated and fully characterized. On the other hand, intermediate 3l (3-MeO-Ph as R^3^) yielded a mixture of oxazepane derivative 6l and lactone 11l in a ratio of approximately 1 : 1, and for this reason, the products were not isolated (Scheme 6). Intermediates 3m, 3o–q, t were synthesized from *p*-substituted 2-bromoacetophenones (4-Me-Ph, 4-F-Ph, 4-Br-Ph and 4-CF_3_-Ph as R^3^) and 3-thienyl derivative yielded oxazepanes 7m, 7o–q, t as separable C2 *R*,*S* diastereomers in variable ratios (Table 2) with combined crude purities in a range of 33–91%. In the case of oxazepane derivative 6r (4-NO_2_-Ph as R^3^), hydrogenation using either Pd/C or PtO_2_ led to cleavage of the heterocyclic scaffold, and compound 10r was isolated (Scheme 5) in 82% crude purity and 50% overall yield (Table 2).

In the cases of 3n (4-MeO-Ph as R^3^) and 3s (4-NH_2_-3,5-diCl-Ph as R^3^), we observed reversed regioselectivity, and after TFA/Et_3_SiH cleavage, lactone derivatives 11n and 11s were formed exclusively; however, 11s was accompanied with oxazepane 6s as the minor product (30% crude purity). Due to the excess triethylsilane in the reaction mixture, the abovementioned derivatives were obtained as phenylethyl derivatives 11 (Scheme 6). The exposure to TFA/Et_3_SiH had to be prolonged to make the reduction quantitative. In combination with the outcome obtained from intermediate 3l (3-MeO-Ph as R^3^), we can state that electron-donating R^3^ groups in the *m*- or *p*-positions contribute to the formation of lactones, as they probably diminish the reactivity of adjacent ketones toward nucleophilic addition, which suppresses the formation of intermediate C (Scheme 2).

Similar to previously reported results,^34^ the alkylation step with 2-chloroacetone provided the corresponding oxazepane 6u in only 50% conversion. For this reason, the product was not isolated.

## Conclusions

To conclude, we developed a simple methodology to prepare 2-phenyl-substituted-1,4-oxazepane-5-carboxylic acid derivatives. Although the formation of the oxazepane scaffold was nonstereoselective, as in the case of serine-based analogs leading to chiral morpholines,^26^ the separation and full characterization of major diastereomers was feasible. The developed strategy provided rather minor limitations, *e.g.*, competitive lactonization in the cases of *m*- and *p*-electron-donating groups as R^3^ substituents. Importantly, the developed protocols and corresponding intermediates can be applied for the synthesis of differently fused 1,4-oxazepanes based on previously reported approaches targeted to fused morpholines.^27–30^

## Experimental section

### General information

Solvents and chemicals were purchased from Sigma-Aldrich (Milwaukee, WI, http://www.sigmaaldrich.com), Acros Organic (Geel, Belgium, http://www.acros.com) and Fluorochem (Hadfield, United Kingdom, http://www.fluorochem.co.uk). Wang resin (100–200 mesh, 1% DVB, 1.4 mmol g^−1^), Rink resin (100–200 mesh, 1% DVB, 0.4 mmol g^−1^) and aminomethyl resin (100–200 mesh, 1% DVB, 0.98 mmol g^−1^) were obtained from AAPPTec (Louisville, KY, http://www.aapptec.com). Solid-phase synthesis was carried out in plastic reaction vessels (syringes, each equipped with a porous disk) using a manually operated synthesizer (Torviq, Niles, MI, http://www.torviq.com). All reactions were carried out at ambient temperature (23 °C) unless stated otherwise. The synthesis of Fmoc-HSe(TBDMS)-OH,^31,32^ immobilization of Fmoc-HSe(TBDMS)-OH on the resin^26^ and *N*-phenacyl sulfonamides 3a–u^26^ was performed according to these reported protocols. The LC-MS analyses were carried out on UHPLC-MS system consisting of UHPLC chromatograph Acquity with photodiode array detector and single quadrupole mass spectrometer (Waters), using X-Select C18 column with the mobile phase consisting of 10 mM ammonium acetate (AmAc) in H_2_O and MeCN. The ESI source operated at discharge current of 5 μA, vaporizer temperature of 350 °C and capillary temperature of 200 °C. For the LC/MS analysis, a sample of resin (∼5 mg) was treated with TFA in CH_2_Cl_2_, the cleavage cocktail was evaporated under a stream of nitrogen, and cleaved compounds extracted into MeCN/H_2_O (20% or 50%; 1 mL). Purification was carried out on C18 semipreparative RP-HPLC with the gradient of 10 mM aqueous AmAc and MeCN, flow rate 15 mL min^−1^ or by normal phase by silica gel chromatography. Residual solvents (H_2_O and AmAc buffer) were lyophilized by the ScanVac Coolsafe 110-4 working at −110 °C. All 1D and 2D NMR experiments were performed with using ECX500 spectrometer (JEOL RESONANCE, Tokyo, Japan) at magnetic field strength of 11.75 T corresponding to ^1^H and ^13^C resonance frequencies of 500.16 MHz and 125.77 MHz at 27 °C. Chemical shifts (*δ*) are reported in parts per million (ppm) and coupling constants (*J*) are reported in Hertz (Hz). The signals of MeCN-*d*_3_ were set at 1.94 ppm in ^1^H NMR spectra and at 118.26 ppm in ^13^C NMR spectra. ^15^N chemical shifts were referenced to external 90% formamide in DMSO-*d*_6_ at 112.00 ppm.^35^ The assignment of ^1^H, ^13^C{^1^H} and ^15^N signals was done by APT, ^1^H–^1^H COSY, ^1^H–^1^H NOESY, ^1^H–^13^C HMQC, ^1^H–^13^C HMBC and ^1^H–^15^N HMBC. Abbreviations in NMR spectra: br. s – broad singlet, br. d – broad doublet, s – singlet, d – doublet, dd – doublet of doublets, ddd – doublet of doublets of doublets, dddd – doublet of doublets of doublets of doublets, m – multiplet. Acetate salt (residual agent from the semipreparative HPLC purification) exhibited a singlet at 2.09–2.53 ppm in ^1^H NMR spectra and two resonances at 41.00 ppm and 171.00 ppm in ^13^C NMR spectra. HRMS analysis was performed using LC-MS (Dionex Ultimate 3000, Thermo Fischer Scientific, MA, USA) with Exactive Plus Orbitrap high-resolution mass spectrometer (Thermo Exactive Plus, Thermo Fischer Scientific, MA, USA) operating at positive or negative full scan mode (120 000 FWMH) in the range of 100–1000 *m*/*z* with electrospray ionization working at 150 °C and the source voltage of 3.6 kV. Chromatographic separation was performed on column Phenomenex Gemini (C18, 50 × 2 mm, 3 μm particle) with isocratic elution and mobile phase (MP) containing MeCN/10 mM AmAc (80 : 20; v/v). The samples were dissolved in the initial MP. The acquired data were internally calibrated with phthalate as a contaminant in MeOH (*m*/*z* 297.15909). IR spectra were measured by DRIFT (Diffuse Reflectance Infrared Fourier Transform) on a Thermo Nicolet AVATAR 370 FTIR spectrometer. Absorbance peaks (wavenumbers) are reported in reciprocal centimeters (cm^−1^) and transmittances (*T*) are reported in percentages (%). Specific optical rotations were measured on Automatic Compact Polarimeter POL-1/2 (ATAGO, Japan) with LED Light Source and 589 nm interference filter at 24 °C. The length of cuvette was 2 cm and specific optical rotations are reported as follows: [*α*]^T^_D_, concentration (g mL^−1^) and solvent.

#### General method for calculation of yields using ^1^H NMR

^1^H NMR spectra of external standard at three different concentration levels were measured. In each spectrum, the solvent signal was integrated followed by the integration of selected H^Ar^ signal of external standard. Ratios of solvent/standard signal areas along with known quantity of standard were used to construct a calibration curve. Then, ^1^H NMR spectra of studied sample were measured and the ratio of solvent/sample (selected H^Ar^ signal) areas was determined. Using the calibration curve, the quantity of compound in the sample was calculated.

#### General procedure for cleavage the TBDMS protecting group and lactonization 5a–u

The polymer-supported intermediate 3a–u (500 mg) was cleaved in the mixture of TFA/CH_2_Cl_2_ (5 mL, 50%) for 1 h at room temperature. Then the resin was washed three times with fresh cleavage cocktail (5 mL) and the combined fractions were evaporated using a stream of nitrogen, lyophilized overnight and purified by semipreparative RP HPLC.

#### (−)-(*S*)-2-Nitro-*N*-(2-oxo-2-phenethyl)-*N*-(2-oxotetrahydrofuran-3-yl)benzenesulfonamide 5a

Cleaved from 742 mg of resin 3a (0.528 mmol g^−1^, 0.342 mmol of substrate). White amorphous solid (117.4 mg, 0.291 mmol, 74%). HPLC purity 99%. ^1^H NMR (500 MHz, MeCN-*d*_3_): *δ* = 8.26 (dd, *J* = 7.7, 1.6 Hz, 1H, HC9), 7.94–7.96 (m, 2H, HC^18,22^), 7.74–7.83 (m, 3H, HC^10–12^), 7.63–7.67 (m, 1H, HC^20^), 7.50–7.54 (m, 2H, HC^19,21^), 5.25 (d, *J* = 19.2 Hz, 1H, H_a_C^15^), 5.05 (dd, *J* = 12.4, 8.7 Hz, 1H, HC^3^), 4.60 (d, *J* = 19.2 Hz, 1H, H_b_C^15^), 4.31 (ddd, *J* = 9.0, 9.0, 1.0 Hz, 1H, H_b_C^5^), 4.24 (ddd, *J* = 11.2, 9.0, 5.8 Hz, 1H, H_a_C^5^), 2.57 (dddd, *J* = 12.7, 8.7, 5.8, 1.0 Hz, 1H, H_b_C^4^), 2.29 (dddd, *J* = 12.5, 12.5, 11.2, 9.0 Hz, 1H, H_a_C^4^). ^13^C{^1^H} NMR (126 MHz, MeCN-*d*_3_): *δ* = 194.7 (C16), 174.4 (C2), 148.9 (C13), 135.5 (C17), 135.4 (C9), 134.9 (C20), 133.8 (C8), 133.21 (C10), 131.6 (C12), 129.8 (C19,21), 128.9 (C18,22), 125.1 (C11), 66.7 (C5), 58.6 (C3), 53.2 (C15), 28.4 (C4). ^15^N NMR (51 MHz, MeCN-*d*_3_): *δ* = 90.0 (N6), 372.9 (N14). HRMS (ESI, pos.): *m*/*z* calcd for C_18_H_17_N_2_O_7_S [M + H]^+^ 405.0751 found 405.0750. IR (DRIFT): *ν̄* = 3101, 3953, 2930, 2918, 1780, 1595, 1544, 1378, 1340, 1301, 1206, 1157, 1080, 1063, 760, 742, 700 cm^−1^. [*α*]^25^_D_ = −21.0° (*c* = 0.00067 g mL^−1^ in MeCN).

#### General procedure for cyclization to 1,4-oxazepanes 6a–u

The polymer-supported intermediate 3a–u (500 mg) was cleaved in TFA/Et_3_SiH/CH_2_Cl_2_ (5 mL, 10 : 1 : 9) for 30 min (except for 3n and 3s) or 24 h (for derivatives 3n and 3s) at room temperature. Then the resin was washed three times with fresh cleavage cocktail (5 mL) and the combined fractions were evaporated using a stream of nitrogen and lyophilized overnight. The crude products were purified using RP-HPLC.

#### (−)-Ammonium (2*R*,5*S*)-2-phenyl-4-tosyl-1,4-oxazepane-5-carboxylate 6g

Cleaved from 860 mg of resin 3g (0.273 mmol g^−1^, 0.235 mmol of substrate). The separable mixture of C2 *R* : *S* diastereomers in a ratio of 62 : 38, the isolation of major C2 *R* epimer was performed. White amorphous solid (16.1 mg, 0.043 mmol, 18%). HPLC purity 97%. NMR: mixture with 3% of C2 *S* isomer. ^1^H NMR (500 MHz, MeCN-*d*_3_): *δ* = 7.75 (d, *J* = 8.3 Hz, 2H), 7.23–7.37 (m, 7H), 4.81 (br. s, 5H, residual water), 4.55 (dd, *J* = 10.6, 7.1 Hz, 1H), 4.32 (d, *J* = 9.1 Hz, 1H), 4.02 (ddd, *J* = 12.7, 6.3, 1.5 Hz, 1H), 3.78 (ddd, *J* = 15.9, 1.0, 1.0 Hz, 1H), 3.47–3.55 (m, 1H), 3.49 (ddd, *J* = 12.7, 9.5, 1.2 Hz, 1H), 2.47–2.54 (m, 1H), 2.39 (s, 3H), 2.13 (dddd, *J* = 15.9, 10.6, 9.1, 1.6 Hz, 1H). ^13^C{^1^H} NMR (126 MHz, MeCN-*d*_3_): *δ* = 175.7, 144.8, 141.0, 138.9, 130.7, 129.3, 128.7, 128.2, 127.0, 83.2, 67.7, 59.5, 54.4, 35.7, 21.5. HRMS (ESI, pos.): *m*/*z* calcd for C_19_H_25_N_2_O_5_S [M + H]^+^ 393.1479 found 393.1482. IR (DRIFT): *ν̄* = 3062, 3029, 2950, 2862, 11 917, 1723, 1596, 1450, 1334, 1306, 1171, 1152, 761, 746, 699 cm^−1^. [*α*]^24^_D_ = −36.2° (*c* = 0.00062 g mL^−1^, MeCN).

#### (−)-(*S*)–*N*-(4-Methoxyphenethyl)-2-nitro-*N*-(2-oxotetrahydrofuran-3-yl)benzenesulfonami-de 11n

Cleaved from 577 mg of resin 3n (0.225 mmol g^−1^, 0.130 mmol of substrate) for 24 h at room temperature. Pale yellow amorphous solid (17.8 mg, 0.042 mmol, 33%). HPLC purity 99%. ^1^H NMR (500 MHz, MeCN-*d*_3_): *δ* = 8.13 (dd, *J* = 7.7, 1.5 Hz, 1H), 7.72–7.82 (m, 3H), 7.11 (d, *J* = 8.5 Hz, 2H), 6.83 (d, *J* = 8.5 Hz, 2H), 4.90 (dd, *J* = 11.7, 9.0 Hz, 1H), 4.36–4.42 (m, 1H), 4.25 (ddd, *J* = 10.7, 9.0, 6.3 Hz, 1H), 3.75 (s, 3H), 3.45–3.54 (m, 1H), 3.26–3.36 (m, 1H), 2.83–2.90 (m, 2H), 2.48 (dddd, *J* = 12.5, 9.0, 6.3, 1.5 Hz, 1H), 2.33–2.44 (m, 1H). ^13^C{^1^H} NMR (126 MHz, MeCN-*d*_3_): *δ* = 174.7, 159.5, 149.3, 135.5, 133.3, 133.3, 131.4, 131.1, 130.9, 125.1, 114.9, 66.6, 58.5, 55.8, 49.9, 36.1, 27.9. HRMS (ESI, pos.): *m*/*z* calcd for C_19_H_21_N_2_O_7_S [M + H]^+^ 421.1064 found 421.1066. IR (DRIFT): *ν̄* = 2932, 2836, 1783, 1542, 1512, 1367, 1342, 1250, 1241, 1159, 785, 752, 708 cm^−1^. [*α*]^24^_D_ = −76.9° (*c* = 0.00029 g mL^−1^, MeCN).

#### General procedure for catalytical hydrogenation of nitro group 7a–f, h–m, o–r, t, including 8j, 9h, and 10r

The synthesis of anilines 7a–f, h–m, o–r, t and amino-lactones 8j, 9h was performed according to the reported protocol^28^ using 10% Pd/C or PtO_2_ (for derivatives 6l, 6p, 6r, 6t, 5j) for 24 h at room temperature. The crude evaporators were purified by silica gel chromatography in CH_2_Cl_2_/methanol (MeOH) (9/1; v/v, for derivatives 7a–f, h–m, o–r, t) or ethyl acetate (EA)/*n*-hexane (Hex; 4/6 or 1/1; v/v; for derivatives 8j, 9h, respectively) to remove residual catalyst after hydrogenation, and then subjected to RP-HPLC.

#### (−)-(2*R*,5*S*)-4-((2-Aminophenyl)sulfonyl)-2-phenyl-1,4-oxazepa-ne-5-carboxylic acid 7a

##### Prepared from Wang resin

Cleaved from 785 mg of resin 3a (0.140 mmol g^−1^, 0.110 mmol of substrate). The separable mixture of C2 *R* : *S* diastereomers in a ratio of 56 : 44, the isolation of the major C2 *R* epimer was performed. White amorphous solid (14.2 mg, 0.038 mmol, 34%). HPLC purity 97%. NMR: mixture with 2% of C2 *S* isomer. ^1^H NMR (500 MHz, MeCN-*d*_3_): *δ* = 7.66 (dd, *J* = 8.1, 1.5 Hz, 1H, HC^19^), 7.34 (ddd, *J* = 8.3, 7.2, 1.5 Hz, 1H, HC^17^), 7.23–7.30 (m, 3H, HC^10–12^), 7.15 (m, 2H, HC^9,13^), 6.84 (dd, *J* = 8.3, 1.0 Hz, 1H, HC^16^), 6.75 (ddd, *J* = 8.1, 7.2, 1.0 Hz, 1H, HC^18^), 4.66 (dd, *J* = 10.4, 7.1 Hz, 1H, HC^5^), 4.16 (d, *J* = 9.5 Hz, 1H, HC^2^), 4.02 (ddd, *J* = 12.7, 6.3, 1.1 Hz, 1H, H_b_C^7^), 3.70 (d, *J* = 16.1 Hz, 1H, H_a_C^3^), 3.65 (dd, *J* = 12.7, 8.9 Hz, 1H, H_a_C^7^), 3.50 (dd, *J* = 16.1, 9.5 Hz, 1H, H_b_C^3^), 2.54 (ddd, *J* = 15.4, 7.1, 6.3 Hz, 1H, H_a_C^6^), 2.16 (dddd, *J* = 15.4, 10.4, 8.9, 1.1 Hz, 1H, H_b_C^6^). ^13^C{^1^H} NMR (126 MHz, MeCN-*d*_3_): *δ* = 174.8 (C21), 147.5 (C15), 141.0 (C8), 135.4 (C17), 131.0 (C19), 129.3 (C10,12), 128.7 (C11), 127.1 (C9,13), 121.9 (C14), 118.6 (C16), 117.7 (C18), 82.7 (C2), 67.5 (C7), 59.1 (C5), 54.1 (C3), 35.6 (C6). ^15^N NMR (51 MHz, MeCN-*d*_3_): *δ* = 99.9 (N4), 63.0 (N20). HRMS (ESI, pos.): *m*/*z* calcd for C_18_H_21_N_2_O_5_S [M + H]^+^ 377.1166 found 377.1167. IR (DRIFT): *ν̄* = 2950, 2864, 1719, 1617, 1483, 1452, 1317, 1169, 1140, 751, 698 cm^−1^. [*α*]^24^_D_ = −64.1° (*c* = 0.00020 g mL^−1^, MeCN).

##### Prepared from Wang-piperazine resin

Cleaved from 669 mg of resin 3a (0.601 mmol g^−1^, 0.402 mmol of substrate). The separable mixture of C2 *R* : *S* diastereomers in a ratio of 72 : 28, the isolation only of the major C2 *R* epimer was performed. White amorphous solid (14.5 mg, 0.039 mmol, 10%). HPLC purity 99%. NMR: mixture with 2% of C2 *S* isomer. The analytical data (^1^H and ^13^C NMR, HRMS, IR (DRIFT) and [*α*]^24^_D_) corresponded with 7a prepared from Wang resin.

#### (−)-(2*R*,5*S*)-4-((2-Aminophenyl)sulfonyl)-2-phenyl-1,4-oxazepane-5-carboxamide 7b^2^*^R^*

Cleaved from 1000 mg of resin 3b (0.225 mmol g^−1^, 0.225 mmol of substrate). The separable mixture of C2 *R* : *S* diastereomers in a ratio of 45 : 55, the isolation of C2 *R* epimer was performed. White amorphous solid (7.2 mg, 0.019 mmol, 9%). HPLC purity 99%. ^1^H NMR (500 MHz, MeCN-*d*_3_): *δ* = 7.70 (dd, *J* = 8.2, 1.5 Hz, 1H), 7.39 (ddd, *J* = 8.4, 7.1, 1.5 Hz, 1H), 7.26–7.33 (m, 3H), 7.20–7.21 (m, 2H), 6.89 (dd, *J* = 8.4, 1.1 Hz, 1H), 6.80 (ddd, *J* = 8.2, 7.1, 1.1 Hz, 1H), 6.34 (br. s, 1H), 5.68 (br. s, 1H), 5.38 (br. s, 2H), 4.49 (ddd, *J* = 10.8, 7.1, 1.0 Hz, 1H), 4.28 (dd, *J* = 9.3, 1.2 Hz, 1H), 4.05 (ddd, *J* = 12.9, 6.3, 1.5 Hz, 1H), 3.82 (ddd, *J* = 16.2, 1.2 Hz, 1.2, 1H), 3.67 (ddd, *J* = 12.9, 9.3, 1.0 Hz, 1H), 3.53 (dd, *J* = 16.2, 9.6 Hz, 1H), 2.46 (dddd, *J* = 15.7, 7.1, 6.3, 1.0 Hz, 1H), 2.21 (dddd, *J* = 15.7, 10.8, 9.3, 1.5 Hz, 1H). ^13^C{^1^H} NMR (126 MHz, MeCN-*d*_3_): *δ* = 174.2, 147.4, 140.9, 135.7, 131.1, 129.3, 128.7, 127.1, 121.7, 118.8, 118.1, 82.7, 67.4, 59.3, 54.7, 35.6. HRMS (ESI, pos.): *m*/*z* calcd for C_18_H_22_N_3_O_4_S [M + H]^+^ 376.1326 found 376.1326. IR (DRIFT): *ν̄* = 3447, 3352, 3205, 3062, 3030, 2952, 2864, 1675, 1616, 1483, 1452, 1318, 1301, 1140, 748, 698 cm^−1^. [*α*]^24^_D_ = −62.5° (*c* = 0.00024 g mL^−1^, MeCN).

#### (+)-(2*S*,5*S*)-4-((2-Aminophenyl)sulfonyl)-2-phenyl-1,4-oxazepa-ne-5-carboxamide 7b^2^*^S^*

Cleaved from 1000 mg of resin 3b (0.225 mmol g^−1^, 0.225 mmol of substrate). The separable mixture of C2 *R* : *S* diastereomers in a ratio of 45 : 55, the isolation of C2 *S* epimer was performed. White amorphous solid (3.2 mg, 0.009 mmol, 4%). HPLC purity 99%. ^1^H NMR (500 MHz, MeCN-*d*_3_): *δ* = 7.58 (dd, *J* = 8.1, 1.6 Hz, 1H, HC^19^), 7.35 (ddd, *J* = 8.5, 7.2, 1.6 Hz, 1H, HC^17^), 7.28–7.32 (m, 2H, HC^10,12^), 7.24–7.28 (m, 1H, HC^11^), 7.17–7.20 (m, 2H, HC^9,13^), 6.85 (dd, *J* = 8.5, 1.1 Hz, 1H, HC^16^), 6.75 (br. s, 1H, H_b_N22), 6.70 (ddd, *J* = 8.2, 7.2, 1.1 Hz, 1H, HC^18^), 5.99 (br. s, 1H, H_a_N^22^), 5.62 (br. s, 2H, HN^20^), 4.64 (dd, *J* = 8.7, 1.6 Hz, 1H, HC^2^), 4.59 (dd, *J* = 4.5, 4.5 Hz, 1H, HC^5^), 3.94 (ddd, *J* = 12.8, 4.5, 3.0 Hz, 1H, H_b_C^7^), 3.78 (ddd, *J* = 12.8, 11.0, 1.6 Hz, 1H, H_a_C^7^), 3.63 (dd, *J* = 14.2, 1.6 Hz, 1H, H_a_C^3^), 3.43 (dd, *J* = 14.2, 8.7 Hz, 1H, H_b_C^3^), 2.30 (dddd, *J* = 15.8, 4.5, 4.5, 1.6 Hz, 1H, H_a_C^6^), 2.10 (dddd, *J* = 15.8, 11.0, 4.5, 3.0 Hz, 1H, overlap with water, H_b_C^6^). ^13^C{^1^H} NMR (126 MHz, MeCN-*d*_3_): *δ* = 174.4 (C21), 148.0 (C15), 141.9 (C8), 135.7 (C17), 131.3 (C19), 129.3 (C10,12), 128.6 (C11), 126.6 (C9,13), 119.1 (C14), 118.7 (C16), 117.5 (C18), 81.4 (C2), 67.0 (C7), 59.4 (C5), 56.8 (C3), 33.0 (C6). HRMS (ESI, pos.): *m*/*z* calcd for C_18_H_22_N_3_O_4_S [M + H]^+^ 376.1326 found 376.1325. IR (DRIFT): *ν̄* = 3448, 3350, 3216, 3061, 3029, 2969, 2954, 2920, 1676, 1616, 1483, 1452, 1318, 1303, 1142, 752, 699 cm^−1^. [*α*]^24^_D_ = +271.4° (*c* = 0.00007 g mL^−1^, MeCN).

#### (−)-(2*R*,5*S*)-4-((2-Amino-4-methoxyphenyl)sulfonyl)-2-phenyl-1,4-oxazepane-5-carboxylic acid 7e

Cleaved from 606 mg of resin 3e (0.381 mmol g^−1^, 0.230 mmol of substrate). The separable mixture of C2 *R* : *S* diastereomers in a ratio of 69 : 31, the isolation of major C2 *R* epimer was performed. White amorphous solid (20.1 mg, 0.050 mmol, 21%). HPLC purity 99%. ^1^H NMR (500 MHz, MeCN-*d*_3_): *δ* = 7.58 (d, *J* = 8.9 Hz, 1H), 7.22–7.30 (m, 3H), 7.15–7.16 (m, 2H), 6.31–6.35 (m, 2H), 5.02 (br. s, 2H), 4.61 (dd, *J* = 10.9, 7.1 Hz, 1H), 4.20 (d, *J* = 9.5 Hz, 1H), 4.02 (ddd, *J* = 12.7, 6.4, 1.2 Hz, 1H), 3.76 (s, 3H), 3.58–3.39 (m, 2H), 3.50 (dd, *J* = 16.1, 9.5 Hz, 1H), 2.46–2.54 (m, 1H), 2.16 (dddd, *J* = 15.4, 10.9, 9.2, 1.2 Hz, 1H). ^13^C{^1^H} NMR (126 MHz, MeCN-*d*_3_): *δ* = 175.8, 165.5, 149.5, 141.1, 133.1, 129.3, 128.6, 127.0, 114.3, 105.4, 101.5, 82.7, 67.6, 59.5, 56.1, 54.1, 35.7. HRMS (ESI, pos.): *m*/*z* calcd for C_19_H_23_N_2_O_6_S [M + H]^+^ 407.1271 found 407.1269. IR (DRIFT): *ν̄* = 3469, 3370, 3228, 3029, 2939, 2864, 1736, 1725, 1605, 1450, 1303, 1133, 752, 699, 689 cm^−1^. [*α*]^24^_D_ = −16.8° (*c* = 0.00030 g mL^−1^, MeCN).

#### (−)-(2*RS*,5*S*)-4-((2-Amino-4-chlorophenyl)sulfonyl)-2-phenyl-1,4-oxazepane-5-carboxylic acid 7f^2^*^RS^*

Cleaved from 658 mg of resin 3f (0.381 mmol g^−1^, 0.251 mmol of substrate). The inseparable mixture of C2 *R* : *S* diastereomers in a ratio of 70 : 30, the interpretation of major C2 *R* epimer was performed. White amorphous solid (21.0 mg, 0.051 mmol, 20%). HPLC purity 78%. NMR: mixture with 22% of C2 *S* isomer. ^1^H NMR (500 MHz, MeCN-*d*_3_): *δ* = 7.71 (d, *J* = 8.5 Hz, 1H), 7.39 (s, 1H), 7.29–7.30 (m, 2H), 7.20–7.26 (m, 3H), 6.90 (d, *J* = 8.3 Hz, 1H), 6.01 (br. s, 2H), 4.66 (dd, *J* = 10.3, 7.2 Hz, 1H), 4.41 (d, *J* = 9.6 Hz, 1H), 4.06 (ddd, *J* = 12.6, 6.3, 1.5 Hz, 1H), 3.71 (ddd, *J* = 12.6, 8.9, 1.0 Hz, 1H), 3.65 (d, *J* = 16.0 Hz, 1H), 3.46 (dd, *J* = 16.0, 9.6 Hz, 1H), 2.51–2.60 (m, 1H), 2.19 (dddd, *J* = 15.6, 10.5, 8.9, 1.5 Hz, 1H). ^13^C{^1^H} NMR (126 MHz, MeCN-*d*_3_): *δ* = 175.7, 151.0, 148.6, 141.0, 132.6, 129.3, 128.7, 127.0, 121.4, 119.8, 114.7, 82.9, 67.6, 59.4, 54.5, 35.7. HRMS (ESI, pos.): *m*/*z* calcd for C_18_H_20_ClN_2_O_5_S [M + H]^+^ 411.0776 found 411.0777. IR (DRIFT): *ν̄* = 3303, 3064, 3029, 2970, 2950, 2868, 1736, 1727, 1587, 1450, 1317, 1292, 1163, 758, 699 cm^−1^. [*α*]^24^_D_ = −17.3° (*c* = 0.00098 g mL^−1^, MeCN).

#### (−)-(2*R*,5*S*)-4-((2-Aminophenyl)sulfonyl)-2-(2-fluorophenyl)-1,4-oxazepane-5-carboxylic acid 7i^2^*^R^*

Cleaved from 386 mg of resin 3i (0.273 mmol g^−1^, 0.105 mmol of substrate). The separable mixture of C2 *R* : *S* diastereomers in a ratio of 29 : 71, the isolation of major C2 *R* epimer was performed. White amorphous solid (1.6 mg, 0.004 mmol, 4%). HPLC purity 94%. ^1^H NMR (500 MHz, MeCN-*d*_3_): *δ* = 7.64 (dd, *J* = 8.2, 1.5 Hz, 1H), 7.28–7.37 (m, 3H), 7.14 (ddd, *J* = 8.6, 7.5, 1.1 Hz, 1H), 7.06 (ddd, *J* = 10.6, 8.3, 1.1 Hz, 1H), 6.82 (dd, *J* = 8.2, 1.1 Hz, 1H), 6.72 (ddd, *J* = 8.2, 7.2, 1.1 Hz, 1H), 4.69 (dd, *J* = 10.1, 7.2 Hz, 1H), 4.64 (dd, *J* = 9.7, 0.9 Hz, 1H), 4.03 (ddd, *J* = 12.7, 6.4, 1.9 Hz, 1H), 3.85 (ddd, *J* = 15.8, 0.9, 0.9 Hz, 1H), 3.68 (ddd, *J* = 12.7, 8.9, 1.4 Hz, 1H), 3.55 (ddd, *J* = 15.8, 9.7 Hz, 1H), 2.54 (dddd, *J* = 15.6, 7.2, 6.4, 1.4 Hz, 1H, overlap with water), 2.19 (dddd, *J* = 15.6, 10.1, 8.9, 1.9 Hz, 1H). ^13^C{^1^H} NMR (126 MHz, MeCN-*d*_3_): *δ* = 175.5, 160.4 (d, *J* = 243.2 Hz), 147.4, 135.3, 130.9, 130.6 (d, *J* = 9.1 Hz), 129.2, 129.2, 127.7 (d, *J* = 13.1 Hz), 125.3 (d, *J* = 3.0 Hz), 121.8, 117.7, 116.8 (d, *J* = 26.2 Hz), 76.9, 67.4, 58.4, 52.6, 35.4. HRMS (ESI, pos.): *m*/*z* calcd for C_18_H_20_FN_2_O_5_S [M + H]^+^ 395.1071 found 395.1072. IR (DRIFT): *ν̄* = 3462, 3372, 3214, 2926, 2867, 1724, 1617, 1567, 1483, 1453, 1317, 1142, 753 cm^−1^. [*α*]^24^_D_ = −68.2° (*c* = 0.00011 g mL^−1^, MeCN).

#### (−)-(2*S*,5*S*)-4-((2-Aminophenyl)sulfonyl)-2-(2-fluorophenyl)-1,4-oxazepane-5-carboxylic acid 7i^2^*^S^*

Cleaved from 386 mg of resin 3i (0.273 mmol g^−1^, 0.105 mmol of substrate). The separable mixture of C2 *R* : *S* diastereomers in a ratio of 29 : 71, the isolation of C2 *R* epimer was performed. White amorphous solid (4.1 mg, 0.010 mmol, 10%). HPLC purity 93%. NMR: mixture with 7% of C2 *R* isomer. ^1^H NMR (500 MHz, MeCN-*d*_3_): *δ* = 7.57 (dd, *J* = 8.0, 1.5 Hz, 1H), 7.40 (ddd, *J* = 7.5, 7.5, 1.7 Hz, 1H), 7.26–7.31 (m, 2H), 7.14 (ddd, *J* = 8.6, 7.5, 1.1 Hz, 1H), 7.02 (ddd, *J* = 10.6, 8.2, 1.1 Hz, 1H), 6.79 (dd, *J* = 8.2, 1.1 Hz, 1H), 6.65 (ddd, *J* = 8.2, 7.2, 1.1 Hz, 1H), 4.93 (dd, *J* = 8.9, 2.0 Hz, 1H), 4.78 (dd, *J* = 4.8, 4.8 Hz, 1H), 4.02 (ddd, *J* = 13.0, 3.8, 3.8 Hz, 1H), 3.78 (ddd, *J* = 13.0, 11.2, 2.0 Hz, 1H), 3.61 (dd, *J* = 13.8, 2.0 Hz, 1H), 3.43 (dd, *J* = 13.8, 8.9 Hz, 1H), 2.37 (dddd, *J* = 15.6, 4.8, 3.8, 2.0 Hz, 1H), 2.24 (dddd, *J* = 15.6, 11.2, 4.8, 3.8 Hz, 1H). ^13^C{^1^H} NMR (126 MHz, MeCN-*d*_3_): *δ* = 174.3, 159.9 (d, *J* = 244.2 Hz), 148.0, 135.3, 131.1, 130.5 (d, *J* = 8.1 Hz), 128.8, 128.7, 128.6 (d, *J* = 4.0 Hz), 125.4 (d, *J* = 3.0 Hz), 119.7, 117.1, 116.0 (d, *J* = 21.2 Hz), 76.0, 68.0, 59.9, 54.7, 33.8. HRMS (ESI, pos.): *m*/*z* calcd for C_18_H_20_FN_2_O_5_S [M + H]^+^ 395.1071 found 395.1072. IR (DRIFT): *ν̄* = 3463, 3370, 2920, 2863, 1719, 1618, 1566, 1484, 1453, 1318, 1141, 753 cm^−1^. [*α*]^24^_D_ = −93.0° (*c* = 0.00050 g mL^−1^, MeCN).

#### (−)-(2*R*,5*S*)-4-((2-Aminophenyl)sulfonyl)-2-(3-fluorophenyl)-1,4-oxazepane-5-carboxylic acid 7k

Cleaved from 600 mg of resin 3k (0.353 mmol g^−1^, 0.212 mmol of substrate). The separable mixture of C2 *R* : *S* diastereomers in a ratio of 64 : 36, the isolation of major C2 *R* epimer was performed. White amorphous solid (8.5 mg, 0.022 mmol, 10%). HPLC purity 99%. ^1^H NMR (500 MHz, MeCN-*d*_3_): *δ* = 7.68 (dd, *J* = 8.1, 1.5 Hz, 1H), 7.34 (ddd, *J* = 8.4, 7.1, 1.5 Hz, 1H), 7.29 (ddd, *J* = 7.9, 7.9, 6.0 Hz, 1H), 6.87–6.99 (m, 3H), 6.85 (dd, *J* = 8.3, 1.0 Hz, 1H), 6.75 (ddd, *J* = 8.1, 7.1, 1.0 Hz, 1H), 4.62 (dd, *J* = 10.8, 7.2 Hz, 1H), 4.19 (d, *J* = 9.2 Hz, 1H), 4.02 (ddd, *J* = 12.6, 6.3, 1.1 Hz, 1H), 3.70 (ddd, *J* = 15.8, 1.1, 1.1 Hz, 1H), 3.65 (ddd, *J* = 12.6, 9.2, 0.7 Hz, 1H), 3.50 (dd, *J* = 16.2, 9.6 Hz, 1H), 2.45–2.56 (m, 1H), 2.15 (dddd, *J* = 15.8, 10.8, 9.2, 1.2 Hz, 1H). ^13^C{^1^H} NMR (126 MHz, MeCN-*d*_3_): *δ* = 175.4, 163.6 (d, *J* = 243.2 Hz), 147.5, 143.7 (d, *J* = 7.1 Hz), 135.4, 131.1 (d, *J* = 9.1 Hz), 131.0, 123.0 (d, *J* = 2.0 Hz), 121.8, 118.6, 117.6, 115.3 (d, *J* = 21.2 Hz), 113.8 (d, *J* = 23.2 Hz), 82.0, 67.6, 59.4, 54.0, 36.0. HRMS (ESI, pos.): *m*/*z* calcd for C_18_H_20_FN_2_O_5_S [M + H]^+^ 395.1071 found 395.1073. IR (DRIFT): *ν̄* = 3469, 3371, 2932, 2867, 1719, 1616, 1566, 1482, 1450, 1318, 1137, 89, 785, 748, 688 cm^−1^. [*α*]^24^_D_ = −25.7° (*c* = 0.00070 g mL^−1^, MeCN).

#### (+)-(2*R*,5*S*)-4-((2-Aminophenyl)sulfonyl)-2-(*p*-tolyl)-1,4-oxaze-pane-5-carboxylic acid 7m

Cleaved from 615 mg of resin 3m (0.173 mmol g^−1^, 0.168 mmol of substrate). The separable mixture of C2 *R* : *S* diastereomers in a ratio of 77 : 23, the isolation of major C2 *R* epimer was performed. White amorphous solid (8.0 mg, 0.021 mmol, 19%). HPLC purity 99%. ^1^H NMR (500 MHz, MeCN-*d*_3_): *δ* = 7.66 (dd, *J* = 8.1, 1.5 Hz, 1H), 7.34 (ddd, *J* = 8.4, 7.1, 1.5 Hz, 1H), 7.11 (d, *J* = 7.9 Hz, 2H), 7.04 (d, *J* = 7.9 Hz, 2H), 6.85 (dd, *J* = 8.2, 1.0 Hz, 1H), 6.75 (ddd, *J* = 8.1, 7.1, 1.0 Hz, 1H), 4.67 (dd, *J* = 10.8, 7.1 Hz, 1H), 4.14 (d, *J* = 9.0 Hz, 1H), 4.01 (ddd, *J* = 12.7, 6.3, 1.5 Hz, 1H), 3.69 (ddd, *J* = 16.2, 1.0, 1.0 Hz, 1H), 3.64 (ddd, *J* = 12.7, 9.0, 1.0 Hz, 1H), 3.49 (dd, *J* = 16.2, 9.6 Hz, 1H), 2.46–2.56 (m, 1H), 2.29 (s, 3H), 2.16 (dddd, *J* = 15.6, 10.8, 9.0, 1.5 Hz, 1H). ^13^C{^1^H} NMR (126 MHz, MeCN-*d*_3_): *δ* = 174.6, 147.5, 138.4, 138.1, 135.4, 131.0, 129.9, 127.0, 122.0, 118.6, 117.6, 82.6, 67.4, 59.1, 54.1, 35.6, 21.1. HRMS (ESI, pos.): *m*/*z* calcd for C_19_H_23_N_2_O_5_S [M + H]^+^ 391.1322 found 391.1324. IR (DRIFT): *ν̄* = 3460, 3368, 3211, 2950, 2865, 1719, 1617, 1599, 1483, 1452, 1317, 1169, 814, 748, 690 cm^−1^. [*α*]^24^_D_ = +34.8° (*c* = 0.00023 g mL^−1^, MeCN).

#### (+)-(2*R*,5*S*)-4-((2-Aminophenyl)sulfonyl)-2-(4-fluorophenyl)-1,4-oxazepane-5-carboxylic acid 7o

Cleaved from 618 mg of resin 3o (0.261 mmol g^−1^, 0.161 mmol of substrate). The separable mixture of C2 *R* : *S* diastereomers in a ratio of 67 : 33, the isolation of major C2 *R* epimer was performed. Pale yellow amorphous solid (6.3 mg, 0.016 mmol, 10%). HPLC purity 99%. NMR: mixture with 6% of C2 *S* isomer ^1^H NMR (500 MHz, MeCN-*d*_3_): *δ* = 7.66 (dd, *J* = 8.1, 1.6 Hz, 1H), 7.33 (ddd, *J* = 8.4, 7.2, 1.6 Hz, 1H), 7.14 (br. d, *J* = 8.6 Hz, 2H), 6.99 (br. d, *J* = 8.9 Hz, 2H), 6.84 (dd, *J* = 8.2, 1.0 Hz, 1H), 6.74 (ddd, *J* = 8.1, 7.1, 1.0 Hz, 1H), 4.58 (dd, *J* = 10.8, 7.0 Hz, 1H), 4.15 (d, *J* = 9.3 Hz, 1H), 3.99 (ddd, *J* = 12.6, 6.3, 1.3 Hz, 1H), 3.60–3.67 (m, 2H), 3.50 (dd, *J* = 16.2, 9.6 Hz, 1H), 2.44–2.55 (m, 1H), 2.14 (dddd, *J* = 15.6, 10.8, 9.2, 1.3 Hz, 1H). ^13^C{^1^H} NMR (126 MHz, MeCN-*d*_3_): *δ* = 176.2, 163.0 (d, *J* = 244.2 Hz), 147.5, 137.2 (d, *J* = 3.0 Hz), 135.4, 131.0, 129.0 (d, *J* = 9.1 Hz), 121.9, 118.6, 117.5, 115.9 (d, *J* = 21.2 Hz), 81.9, 67.7, 59.9, 54.0, 35.7. HRMS (ESI, pos.): *m*/*z* calcd for C_18_H_20_FN_2_O_5_S [M + H]^+^ 395.1071 found 395.1073. IR (DRIFT): *ν̄* = 3460, 3368, 3210, 2929, 2863, 1717, 1601, 1566, 1509, 1483, 1453, 1317, 1300, 1140, 834, 802, 748, 689 cm^−1^. [*α*]^24^_D_ = +35.3° (*c* = 0.00017 g mL^−1^, MeCN).

#### (−)-(2*R*,5*S*)-4-((2-Aminophenyl)sulfonyl)-2-(4-(trifluoromethyl)phenyl)-1,4-oxazepane-5-car-boxylic acid 7q

Cleaved from 510 mg of resin 3q (0.657 mmol g^−1^, 0.335 mmol of substrate). The separable mixture of C2 *R* : *S* diastereomers in a ratio of 61 : 39, the isolation of major C2 *R* epimer was performed. Yellow amorphous solid (8.3 mg, 0.017 mmol, 8%). HPLC purity 97%. NMR: mixture with 4% of C2 *S* isomer. ^1^H NMR (500 MHz, MeCN-*d*_3_): *δ* = 7.77 (dd, *J* = 7.9, 1.3 Hz, 1H), 7.63 (br. d, *J* = 8.2 Hz, 2H), 7.50–7.58 (m, 1H), 7.42 (d, *J* = 8.2 Hz, 1H), 7.39 (br. d, *J* = 8.2 Hz, 2H), 6.94–7.02 (m, 1H), 4.71 (dd, *J* = 9.1, 7.2 Hz, 1H), 4.41 (d, *J* = 9.4 Hz, 1H), 4.08 (ddd, *J* = 12.6, 6.1, 0.9 Hz, 1H), 3.76 (dd, *J* = 16.2 Hz, 1H), 3.74 (dd, *J* = 12.6, 9.1 Hz, 1H), 3.47 (dd, *J* = 16.0, 9.4 Hz, 1H), 2.51–2.63 (m, 1H), 2.13–2.25 (m, 1H). ^13^C{^1^H} NMR (126 MHz, MeCN-*d*_3_): *δ* = 174.3, 149.9, 145.2, 135.6, 130.7, 130.1 (q, *J* = 32.1 Hz), 127.7, 126.2 (q, *J* = 3.9 Hz), 125.4 (q, *J* = 272.0 Hz), 123.2, 120.4, 115.3, 82.3, 67.5, 58.9, 54.1, 41.2, 35.5. HRMS (ESI, pos.): *m*/*z* calcd for C_19_H_20_F_3_N_2_O_5_S [M + H]^+^ 445.1040 found 445.1042. IR (DRIFT): *ν̄* = 3320, 2937, 2871, 1731, 1597, 1459, 1323, 1164, 1149, 835, 758 cm^−1^. [*α*]^24^_D_ = −73.1° (*c* = 0.00039 g mL^−1^, MeCN).

#### (−)-(2*R*,5*S*)-4-((2-Aminophenyl)sulfonyl)-2-(thiophen-3-yl)-1,4-oxazepane-5-carboxylic acid 7t

Cleaved from 570 mg of resin 3t (0.213 mmol g^−1^, 0.121 mmol of substrate). The separable mixture of C2 *R* : *S* diastereomers in a ratio of 93 : 7, the isolation of major C2 *R* epimer was performed. White amorphous solid (10.8 mg, 0.028 mmol, 22%). HPLC purity 98%. NMR: mixture with 2% of C2 *S* isomer. ^1^H NMR (500 MHz, MeCN-*d*_3_): *δ* = 7.76 (dd, *J* = 7.8, 0.9 Hz, 1H), 7.52 (t, *J* = 7.8 Hz, 1H), 7.39 (d, *J* = 8.3 Hz, 1H), 7.32 (dd, *J* = 5.0, 3.0 Hz, 1H), 7.15–7.18 (m, 1H), 6.94 (dd, *J* = 5.0, 1.3 Hz, 2H), 4.64 (dd, *J* = 10.0, 7.3 Hz, 1H), 4.46 (d, *J* = 9.5 Hz, 1H), 4.02 (ddd, *J* = 12.7, 6.4, 1.5 Hz, 1H), 3.74 (d, *J* = 16.0 Hz, 1H), 3.69 (ddd, *J* = 12.7, 9.0, 1.0 Hz, 1H), 3.49 (dd, *J* = 16.0, 9.5 Hz, 1H), 2.47–2.56 (m, 1H), 2.15 (dddd, *J* = 15.6, 10.4, 9.0, 1.5 Hz, 1H). ^13^C{^1^H} NMR (126 MHz, MeCN-*d*_3_): *δ* = 175.2, 150.0, 142.1, 135.5, 130.7, 127.0, 126.9, 123.0, 122.3, 120.2, 115.2, 79.3, 67.5, 59.3, 53.5, 35.7. HRMS (ESI, neg.): *m*/*z* calcd for C_16_H_17_N_2_O_5_S_2_ [M − H]^−^ 381.0573 found 381.0581. IR (DRIFT): *ν̄* = 3297, 3102, 2950, 2864, 1720, 1596, 1458, 1317, 1293, 1216, 1147, 1030, 955, 758 cm^−1^. [*α*]^24^_D_ = −221.9° (*c* = 0.00029 g mL^−1^, MeCN).

#### (−)-(*S*)-3-(1,1-Dioxo-4-(*o*-tolyl)benzo[*f*][1,2,5]thiadiazepin-2(5*H*)-yl)dihydrofuran-2(3*H*)-one 9h

Cleaved from 592 mg of resin 3h (0.521 mmol g^−1^, 0.308 mmol of substrate). Pale yellow amorphous solid (21.6 mg, 0.058 mmol, 19%). HPLC purity 99%. ^1^H NMR (500 MHz, MeCN-*d*_3_): *δ* = 7.77 (dd, *J* = 8.0, 1.6 Hz, 1H), 7.40 (ddd, *J* = 8.3, 7.3, 1.6 Hz, 1H), 7.34 (ddd, *J* = 8.9, 7.3, 1.6 Hz, 1H), 7.26–7.31 (m, 2H), 7.21–7.25 (m, 1H), 6.99 (dd, *J* = 8.3, 0.9 Hz, 1H), 6.91–6.97 (m, 2H), 4.94 (d, *J* = 1.2 Hz, 1H), 4.89 (dd, *J* = 11.3, 8.8 Hz, 1H), 4.35 (ddd, *J* = 9.0, 9.0, 1.6 Hz, 1H), 4.16 (ddd, *J* = 10.8, 9.0, 6.1 Hz, 1H), 2.39–2.48 (m, 1H), 2.38 (s, 3H), 2.05–2.12 (m, 1H). ^13^C{^1^H} NMR (126 MHz, MeCN-*d*_3_): *δ* = 173.9, 143.4, 140.3, 138.5, 136.6, 134.8, 131.3, 131.1, 130.5, 130.3, 128.3, 126.7, 120.9, 120.4, 101.4, 66.7, 58.6, 26.3, 19.5. HRMS (ESI, neg.): *m*/*z* calcd for C_19_H_17_N_2_O_4_S [M − H]^−^ 369.0904 found 369.0909. IR (DRIFT): *ν̄* = 3345, 3066, 3016, 2920, 1775, 1663, 1474, 1335, 1159, 750 cm^−1^. [*α*]^24^_D_ = −42.1° (*c* = 0.00063 g mL^−1^, MeCN).

#### (+)-(*S*)-((2-Aminophenyl)sulfonyl)-l-homoserine 10r

Cleaved from 503 mg of resin 3r (0.657 mmol g^−1^, 0.330 mmol of substrate). Pale yellow amorphous solid (45.6 mg, 0.166 mmol, 50%). HPLC purity 99%. ^1^H NMR (500 MHz, MeCN-*d*_3_): *δ* = 7.63 (dd, *J* = 8.0, 1.4 Hz, 1H), 7.34 (ddd, *J* = 8.3, 7.2, 1.4 Hz, 1H), 6.85 (dd, *J* = 8.23, 1.1 Hz, 1H), 6.75 (ddd, *J* = 8.0, 7.2 1.1 Hz, 1H), 6.09 (br. s, 1H), 5.27 (br. s, 2H), 4.23–4.30 (m, 1H), 4.05–4.14 (m, 2H), 2.27 (dddd, *J* = 12.5, 8.6, 5.9, 1.2 Hz, 1H), 1.96–2.05 (m, 1H). ^13^C{^1^H} NMR (126 MHz, MeCN-*d*_3_): *δ* = 175.2, 147.1, 135.4, 130.2, 121.6, 118.3, 117.6, 66.6, 52.5, 30.7. HRMS (ESI, pos.): *m*/*z* calcd for C_10_H_13_N_2_O_5_S [M − H]^−^ 273.0540 found 273.0546. IR (DRIFT): *ν̄* = 3476, 3375, 3261, 1770, 1619, 1482, 1453, 1318, 754 cm^−1^. [*α*]^24^_D_ = +5.8° (*c* = 0.00119 g mL^−1^, MeCN).

#### Cyclization to benzothiadiazepine 1,1-dioxide 9j

The crude residue 8j was dissolved in CH_2_Cl_2_ (3.8 mL) and TFA (200 μL, 5%) was added. The reaction mixture was shaken for 24 h at room temperature. Then it was evaporated to dryness using a stream of nitrogen. The product was purified by semipreparative RP-HPLC.

#### (−)-(*S*)-3-(4-(2-Bromophenyl)-1,1-dioxobenzo[*f*][1,2,5]thiadiazepin-2(5*H*)-yl)dihydrofuran-2(3*H*)-one 9j

Cleaved from 589 mg of resin 3j (0.521 mmol g^−1^, 0.307 mmol of substrate). Pale yellow amorphous solid (16.8 mg, 0.039 mmol, 13%). HPLC purity 98%. ^1^H NMR (500 MHz, MeCN-*d*_3_): *δ* = 7.78 (dd, *J* = 8.0, 1.6 Hz, 1H), 7.68 (dd, *J* = 8.0, 1.2 Hz, 1H), 7.48 (dd, *J* = 7.6, 1.9 Hz, 1H), 7.40–7.45 (m, 2H), 7.35 (ddd, *J* = 8.0, 7.4, 1.9 Hz, 1H), 7.02 (br. s, 1H), 7.00 (dd, *J* = 8.3, 0.9 Hz, 1H), 6.97 (ddd, *J* = 8.0, 7.4, 0.9 Hz, 1H), 5.00 (d, *J* = 1.2 Hz, 1H), 4.91 (dd, *J* = 11.5, 9.0 Hz, 1H), 4.35 (ddd, *J* = 10.4, 9.0, 1.4 Hz, 1H), 4.15 (ddd, *J* = 10.9, 9.0, 6.1 Hz, 1H), 2.47–2.58 (m, 1H), 2.06 (dddd, *J* = 12.4, 8.8, 6.1, 1.4 Hz, 1H). ^13^C{^1^H} NMR (126 MHz, MeCN-*d*_3_): *δ* = 173.7, 142.4, 140.2, 137.5, 134.9, 134.0, 132.8, 132.1, 131.2, 128.7, 128.5, 124.1, 120.9, 120.8, 102.7, 66.7, 58.7, 26.2. HRMS (ESI, pos.): *m*/*z* calcd for C_18_H_16_BrN_2_O_4_S [M + H]^+^ 435.0009 found 435.0011. IR (DRIFT): *ν̄* = 3349, 2921, 2851, 1776, 1662, 1594, 1475, 1338, 1162, 751 cm^−1^. [*α*]^24^_D_ = −51.7° (*c* = 0.00029 g mL^−1^, MeCN).

## Conflicts of interest

There are no conflicts to declare.

## Supplementary Material

RA-010-D0RA07997A-s001
