# Supplementary material for: Synthesis of chiral 1,4-oxazepane-5-carboxylic acids from polymer-supported homoserine
Source: RSC Adv. 2020 Sep 30;10(59):35906–16. doi: 10.1039/d0ra07997a (PMC9056997; doi:10.1039/d0ra07997a)
Supplement: RA-010-D0RA07997A-s001 [file RA-010-D0RA07997A-s001.pdf]

Electronic Supplementary Material (ESI) for RSC Advances.
This journal is © The Royal Society of Chemistry 2020

Supporting Information

Synthesis of Chiral 1,4-Oxazepane-5-Carboxylic Acids from Polymer-Supported Homoserine

Petra Králová,[1] Barbora Lemrová,[1] Michal Maloň,[2] and Miroslav Soural[1,3]*

[1] Department of Organic Chemistry, Faculty of Science, Palacký University, 771 46 Olomouc, Czech Republic
[2] JEOL (U.K.) Ltd., JEOL House, Silver Court, Watchmead, Welwyn Garden City, Hertfordshire AL7 1LT, United Kingdom
[3] Institute of Molecular and Translational Medicine, Faculty of Medicine and Dentistry, Palacký University, Hněvotínská 5, 779 00 Olomouc, Czech Republic

*Corresponding author. E-mail: miroslav.soural@upol.cz

Content

Analytical Data (NMR, IR, HRMS and optical rotation) of Prepared Derivatives S2

(-)-(*S*)-2-nitro-*N*-(2-oxo-2-phenethyl)-*N*-(2-oxotetrahydrofuran-3-yl)benzenesulfonamide **5a** S2

(-)-ammonium (2*R*,5S)-2-phenyl-4-tosyl-1,4-oxazepane-5-carboxylate **6g** S9

(-)-(2R,5S)-4-((2-aminophenyl)sulfonyl)-2-phenyl-1,4-oxazepane-5-carboxylic acid **7a** S11

(-)-(2*R*,5S)-4-((2-aminophenyl)sulfonyl)-2-phenyl-1,4-oxazepane-5-carboxamide **7b**^{2R}................S18

(+)-(2*S*,5S)-4-((2-aminophenyl)sulfonyl)-2-phenyl-1,4-oxazepane-5-carboxamide **7b**^{2S} S20

(-)-(2*R*,5S)-4-((2-amino-4-methoxyphenyl)sulfonyl)-2-phenyl-1,4-oxazepane-5-carboxylic acid **7e** S25

(-)-(2*RS*,5S)-4-((2-amino-4-chlorophenyl)sulfonyl)-2-phenyl-1,4-oxazepane-5-carboxylic acid **7f**^{2RS} S27

(-)-(2*R*,5S)-4-((2-aminophenyl)sulfonyl)-2-(2-fluorophenyl)-1,4-oxazepane-5-carboxylic acid **7i**^{2R} S29

(-)-(2*S*,5S)-4-((2-aminophenyl)sulfonyl)-2-(2-fluorophenyl)-1,4-oxazepane-5-carboxylic acid **7i**^{2S}.S31

(-)-(2*R*,5S)-4-((2-aminophenyl)sulfonyl)-2-(3-fluorophenyl)-1,4-oxazepane-5-carboxylic acid **7k** ..S33

(+)-(2*R*,5S)-4-((2-aminophenyl)sulfonyl)-2-(*p*-tolyl)-1,4-oxazepane-5-carboxylic acid **7m** S35

(+)-(2*R*,5S)-4-((2-aminophenyl)sulfonyl)-2-(4-fluorophenyl)-1,4-oxazepane-5-carboxylic acid **7o** .S37

(-)-(2*R*,5S)-4-((2-aminophenyl)sulfonyl)-2-(4-(trifluoromethyl)phenyl)-1,4-oxazepane-5-carboxylic acid **7q** S39

(-)-(2*R*,5S)-4-((2-aminophenyl)sulfonyl)-2-(thiophen-3-yl)-1,4-oxazepane-5-carboxylic acid **7t** S41

(-)-(*S*)-3-(1,1-dioxo-4-(*o*-tolyl)benzo[*f*][1,2,5]thiadiazepin-2(5*H*)-yl)dihydrofuran-2(3*H*)-one **9h** S43

(-)-(*S*)-3-(4-(2-bromophenyl)-1,1-dioxobenzo[*f*][1,2,5]thiadiazepin-2(5*H*)-yl)dihydrofuran-2(3*H*)-one **9j** S45

(+)-(*S*)-((2-aminophenyl)sulfonyl)-*L*-homoserine **10r** S47

(-)-(*S*)-*N*-(4-methoxyphenethyl)-2-nitro-*N*-(2-oxotetrahydrofuran-3-yl)benzenesulfonamide **11n** ...S50

Analytical Data (NMR, IR, HRMS and optical rotation) of Prepared Derivatives

(-)-(*S*)-2-nitro-*N*-(2-oxo-2-phenethyl)-*N*-(2-oxotetrahydrofuran-3-yl)benzenesulfonamide 5a

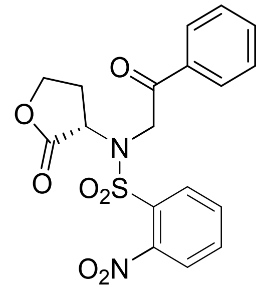

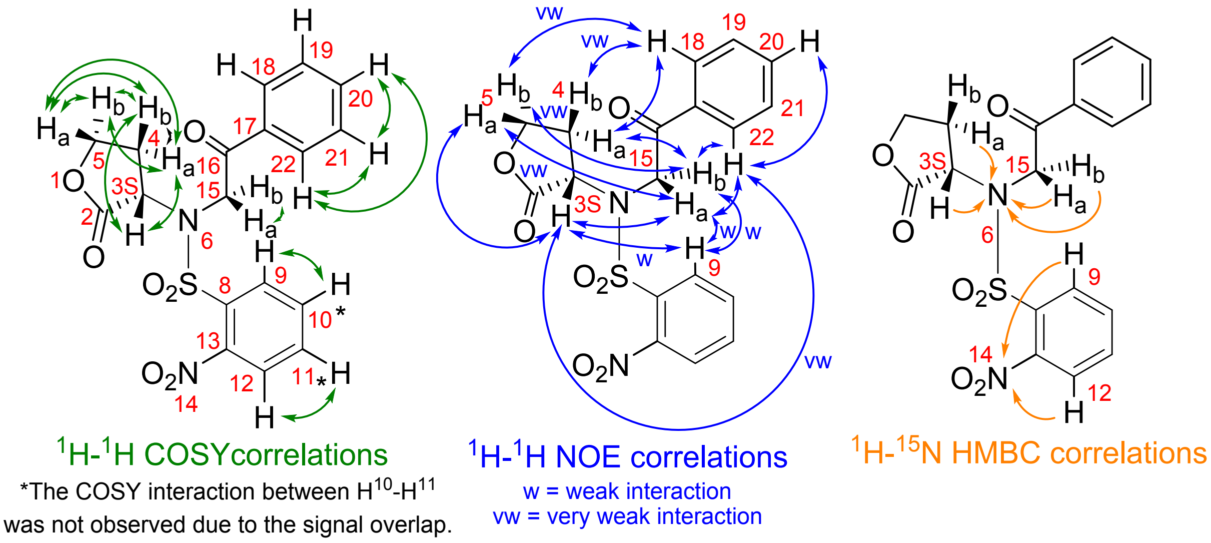

Figure S1. Detailed COSY, NOE and ^{1}H-^{15}N HMBC NMR analysis of lactone **5a**

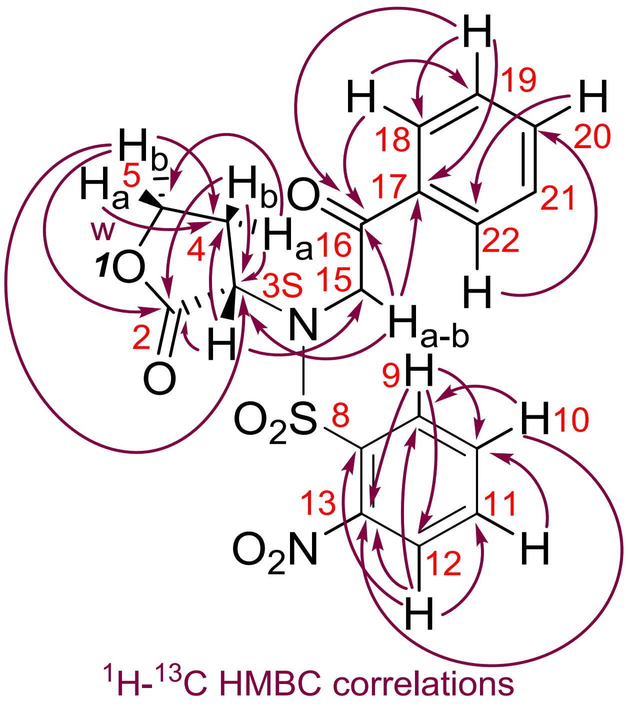

Figure S2. Detailed ^{1}H-^{13}C HMBC NMR analysis of lactone **5a**

Table S1. ^1H NMR (500 MHz) and $^{13}C\{^1H\}$ NMR (126 MHz) data, including detailed COSY, ^1H-^{13}C HMBC and NOE correlations for lactone **5a**[a]

position	^1H NMR δ_H [ppm][b]	splitting pattern, *J* [Hz], integration	$^{13}C\{^1H\}$ NMR δ_C [ppm]	COSY correlations	^1H-^{13}C HMBC correlations	NOE correlations
**2**	-	-	174.4	-	-	-
**3**	5.05	dd, *J* = 12.4, 8.7 Hz, 1H	58.6	H_a^4, H_b^4	28.4, 53.2, 174.4	H_a^5, H^9 (weak int).), H_a^{15}, $H^{18,22}$ (very weak int.)
**4**	H_a : 2.29	dddd, *J* = 12.5, 12.5, 11.2, 9.0 Hz, 1H	28.4	H^3, H_a^5, H_b^5	58.6, 66.2	H_b^{15}, $H^{18,22}$
	H_b: 2.57	dddd, *J* = 12.7, 8.7, 5.8, 1.0 Hz, 1H		H^3, H_a^5, H_b^5 (weak int.)	58.6, 174.4	$H^{18,22}$ (very weak int.)
**5**	H_a : 4.24	ddd, *J* = 11.2, 9.0, 5.8 Hz, 1H	66.7	H_a^4, H_b^4, H_b^5<br>H_a^4, H_b^4 (weak int.), H_a^5	28.4 (weak int.)	H^3, H_a^{15}
	H_b: 4.31	ddd, *J* = 9.0, 9.0, 1.0 Hz, 1H		H_a^5,	28.4, 58.6, 174.4	H_a^{15} (very weak int.), $H^{18,22}$ (very weak int.)
**8**	-	-	133.8	-	-	-
**9**	8.26	dd, *J* = 7.7, 1.6 Hz, 1H	131.6	H^{10}	125.4, 135.4, 148.9	H^{11}, H^{12}
**10**	7.81	m, 1H	125.1	H^9	131.6, 148.9	H^{12}
**11**	7.77	m, 1H	133.3	H^{12}	125.4	H^9
**12**	7.75	m, 1H	135.4	H^{11}	131.6, 133.3, 133.8, 148.9	H^9, H^{10}
**13**	-	-	148.9	-		
**15**	H_a : 5.25	*J* = 19.2 Hz, 1H	53.2	H_b^{15}	58.6, 135.5, 194.7	H^3, H_a^5 (very weak int.), H^9 (weak int.), $H^{18,22}$
	H_b: 4.60	d, *J* = 19.2 Hz, 1H		H_a^{15}	58.6, 135.5, 194.7	H_a^4, H_b^5 (very weak int.), H^9 (weak int.), $H^{18,22}$
**16**	-	-	194.7	-	-	-
**17**	-	-	135.5	-	-	-
**18,22**	7.94-7.96	m, 2H	128.9	$H^{19,21}$	129.8, 134.9, 194.7	H^{20}
**19,21**	7.50-7.54	m, 2H	129.8	$H^{18,22}$, H^{20}	128.9, 135.5, 194.7	-
**20**	7.63-7.67	m, 1H	134.9	H^{21}	128.9	$H^{18,22}$

[a]Assignments are based on extensive 1D and 2D NMR analysis (^1H-^1H COSY, ^1H-^{13}C HMQC, ^1H-^{13}C HMBC and ^1H-^1H NOESY); measured in MeCN-d_3; int. = interaction.

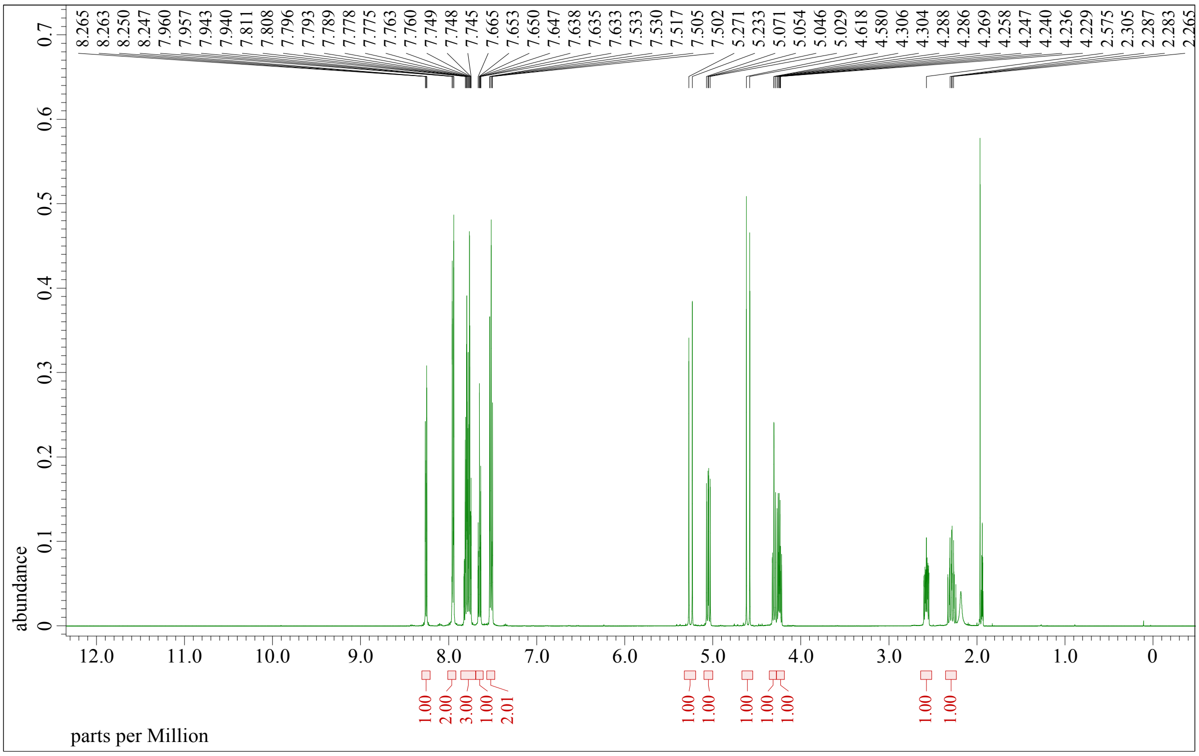

Figure S3. ^{1}H NMR spectrum of **5a** (500 MHz, MeCN-d_3); note: the signal of non-deuterated MeCN at 1.96 ppm and MeCN-d_3 at 1.94 ppm in ratio 62:38 in ^{1}H NMR

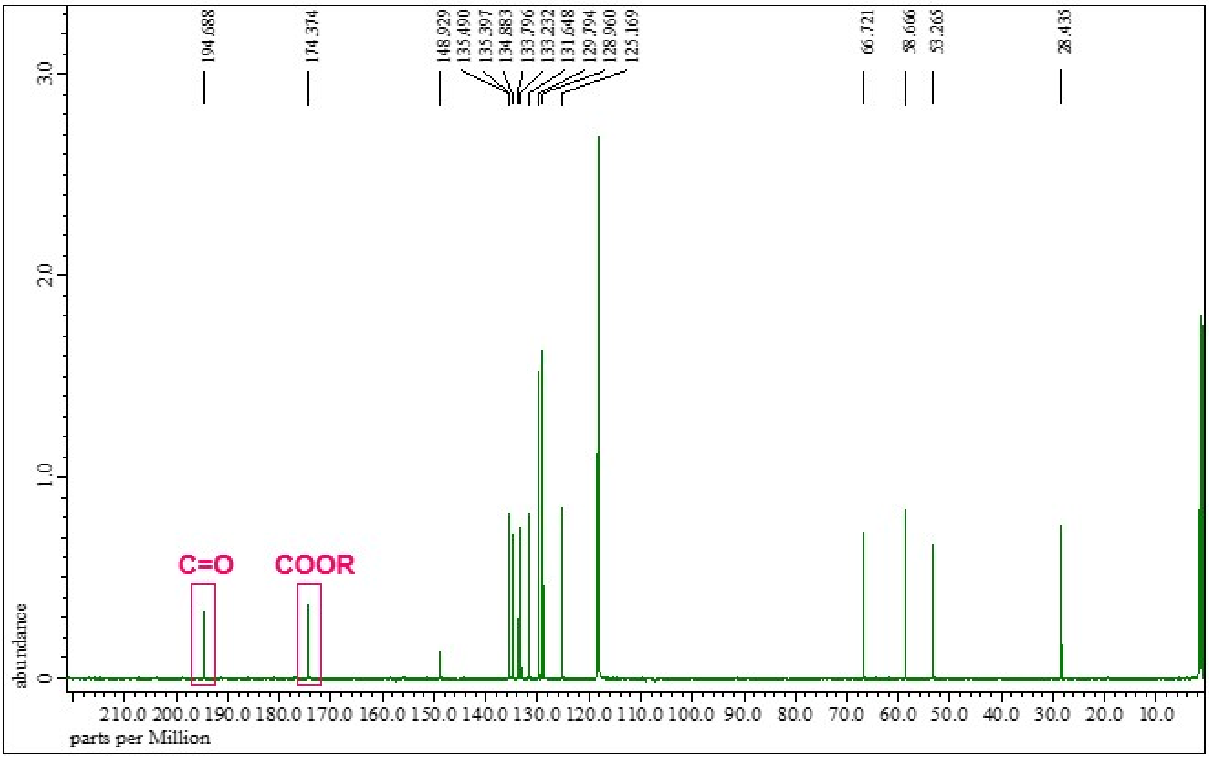

Figure S4. $^{13}C\{^{1}H\}$ NMR spectrum of **5a** (126 MHz, MeCN-d_3)

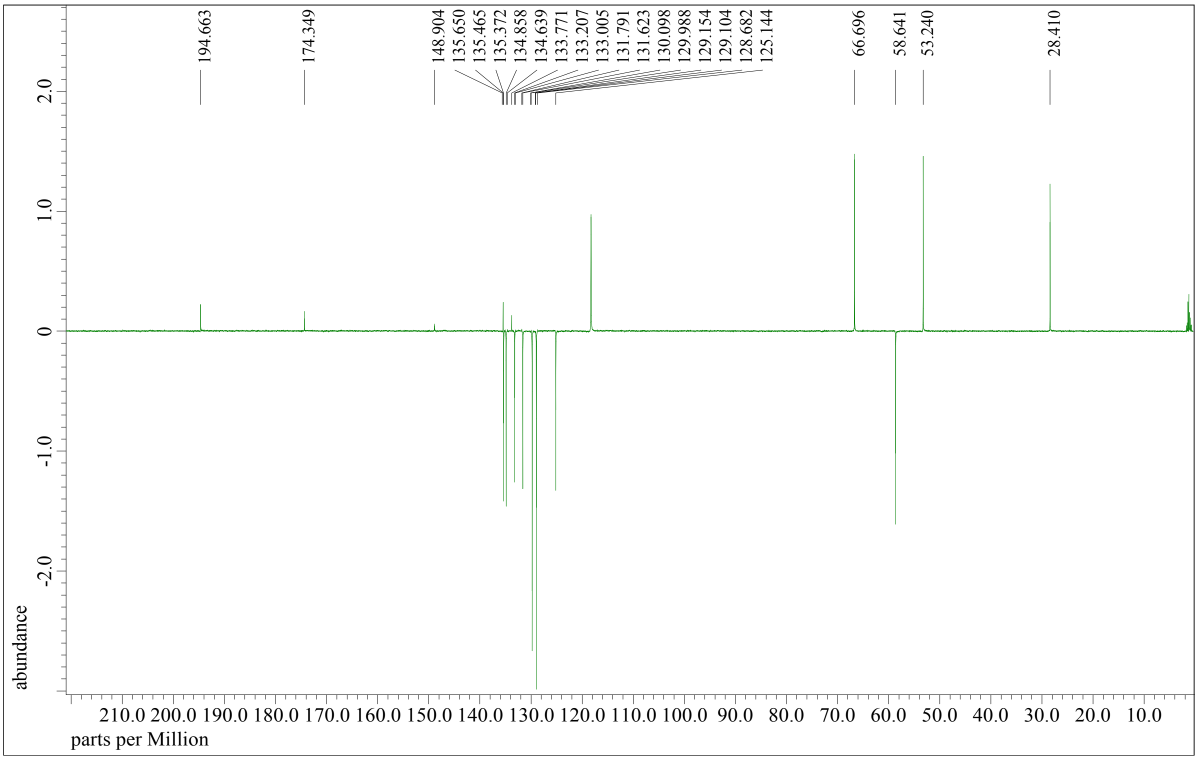

Figure S5. ^{13}C APT NMR spectrum of **5a** (126 MHz, MeCN-d_3)

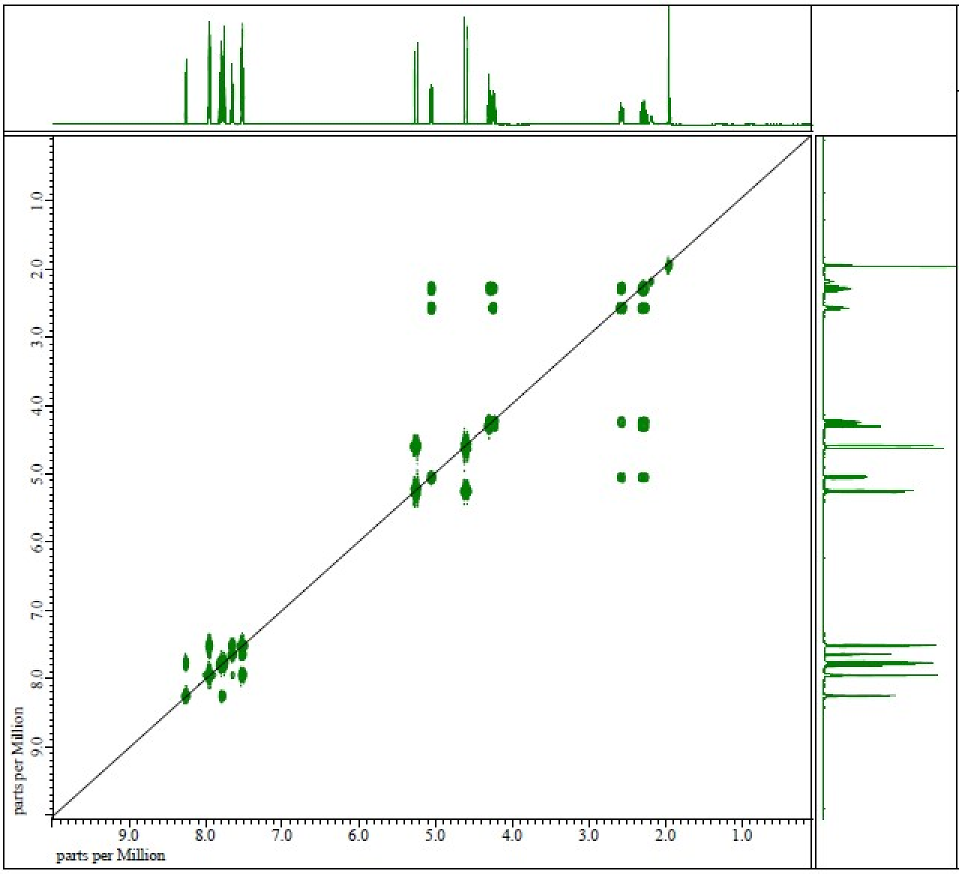

Figure S6. ^{1}H-^{1}H COSY NMR spectrum of **5a** (500 MHz, MeCN-d_3)

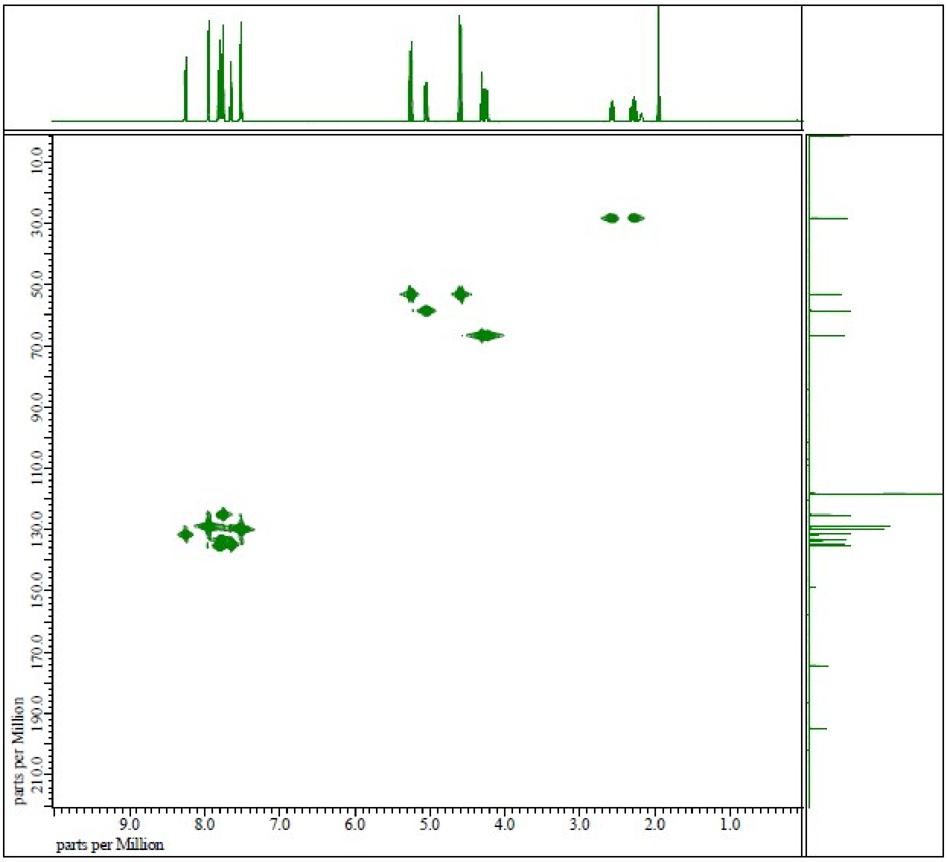

Figure S7. ^{1}H-^{13}C HMQC NMR spectrum of **5a** (MeCN-d_3)

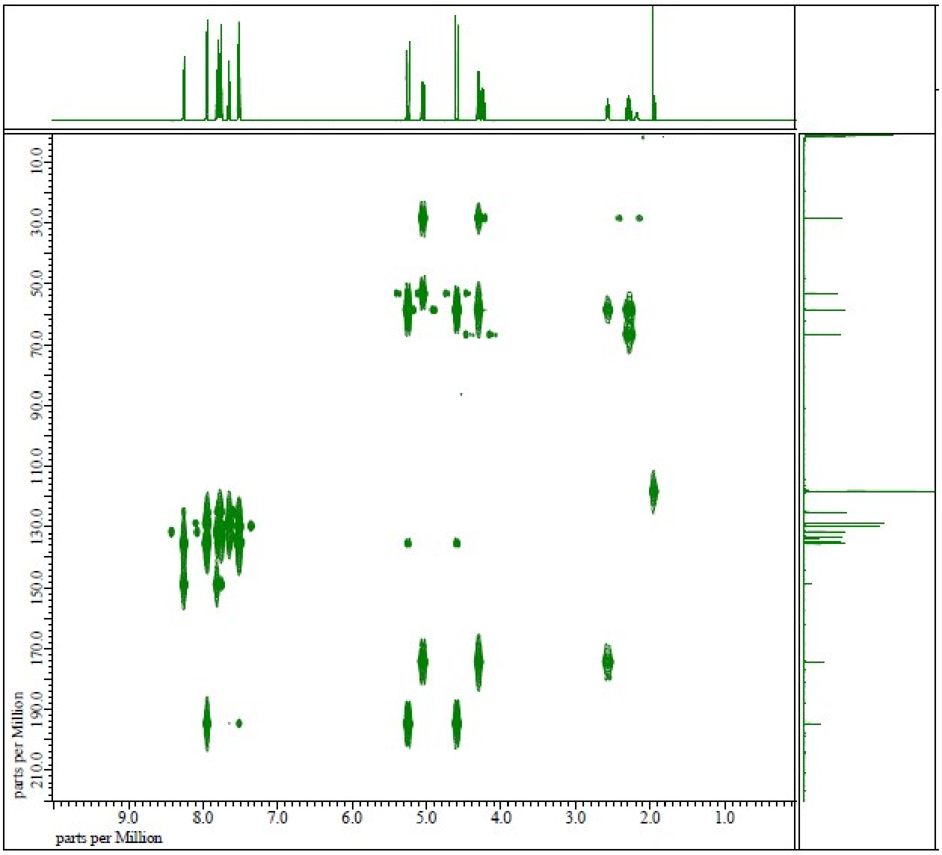

Figure S8. ^{1}H-^{13}C HMBC NMR spectrum of **5a** (MeCN-d_3)

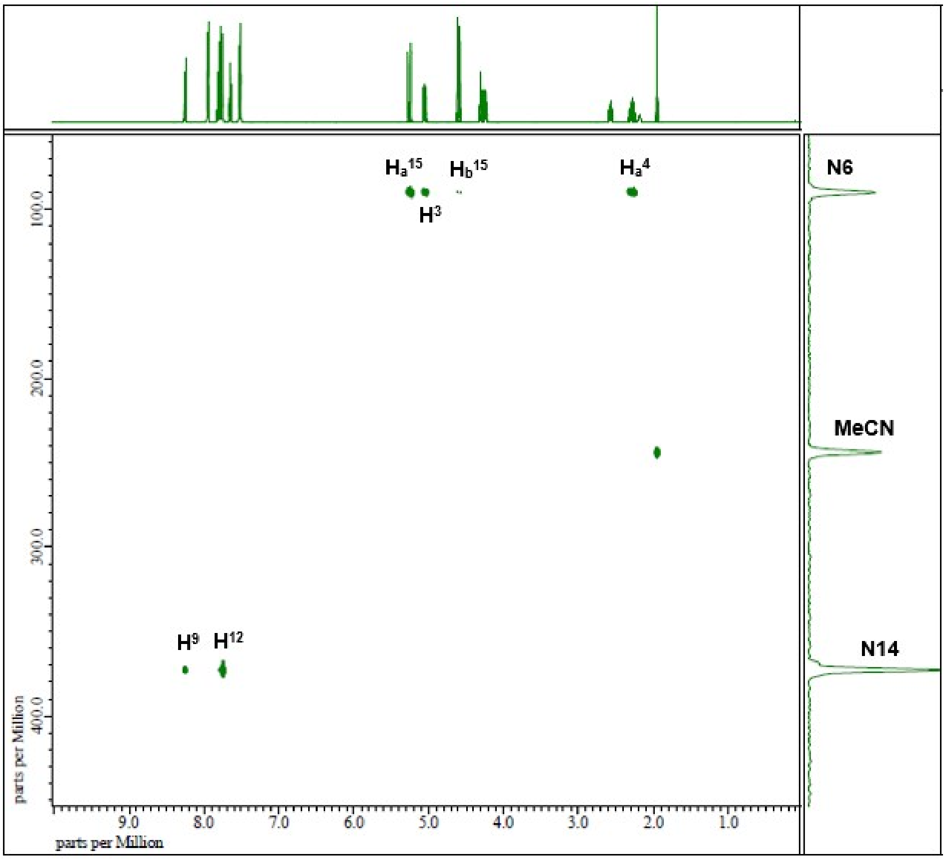

Figure S9. ^{1}H-^{15}N HMBC NMR spectrum of **5a** (MeCN-d_3)

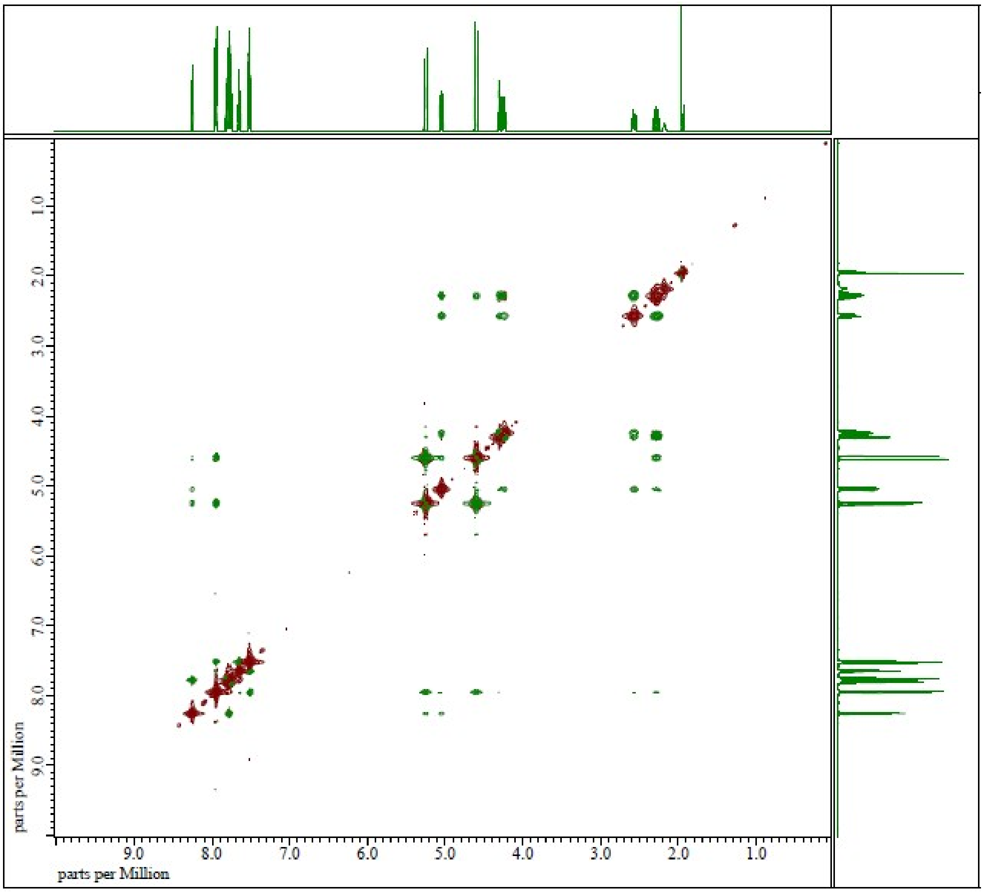

Figure S10. ^{1}H-^{1}H NOESY NMR spectrum of **5a** (MeCN-d_3)

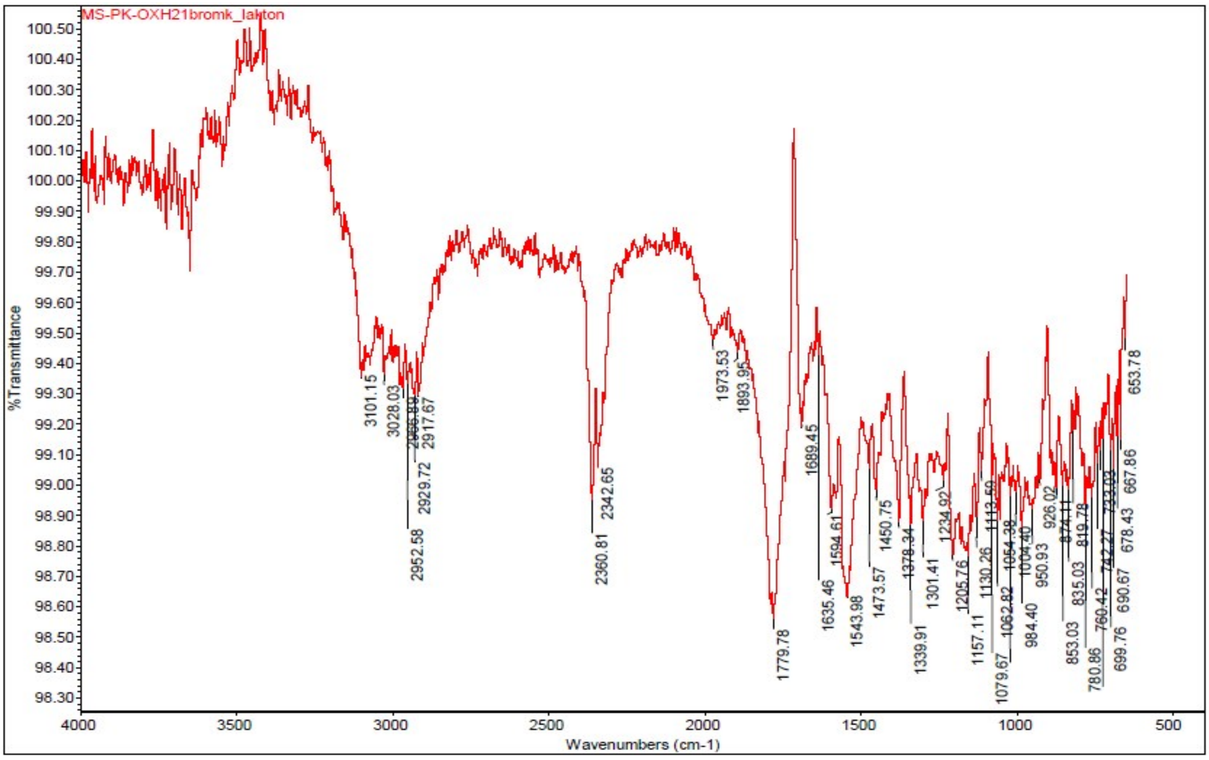

Figure S11. IR spectrum of **5a**

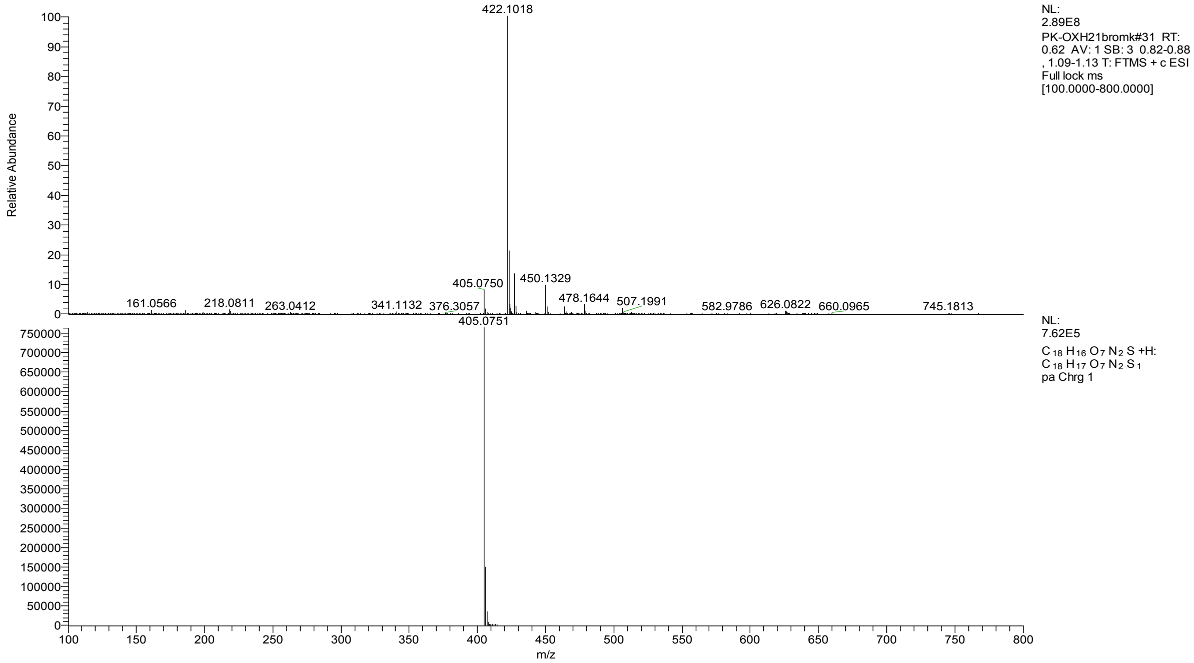

Figure S12. HRMS spectrum of **5a**. Note: ion 422 belongs to the ammonium adduct originating from mobile phase.

(-)-ammonium (2*R*,5S)-2-phenyl-4-tosyl-1,4-oxazepane-5-carboxylate 6g

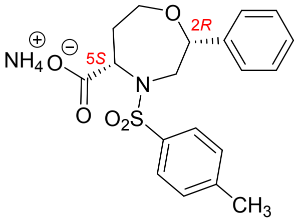

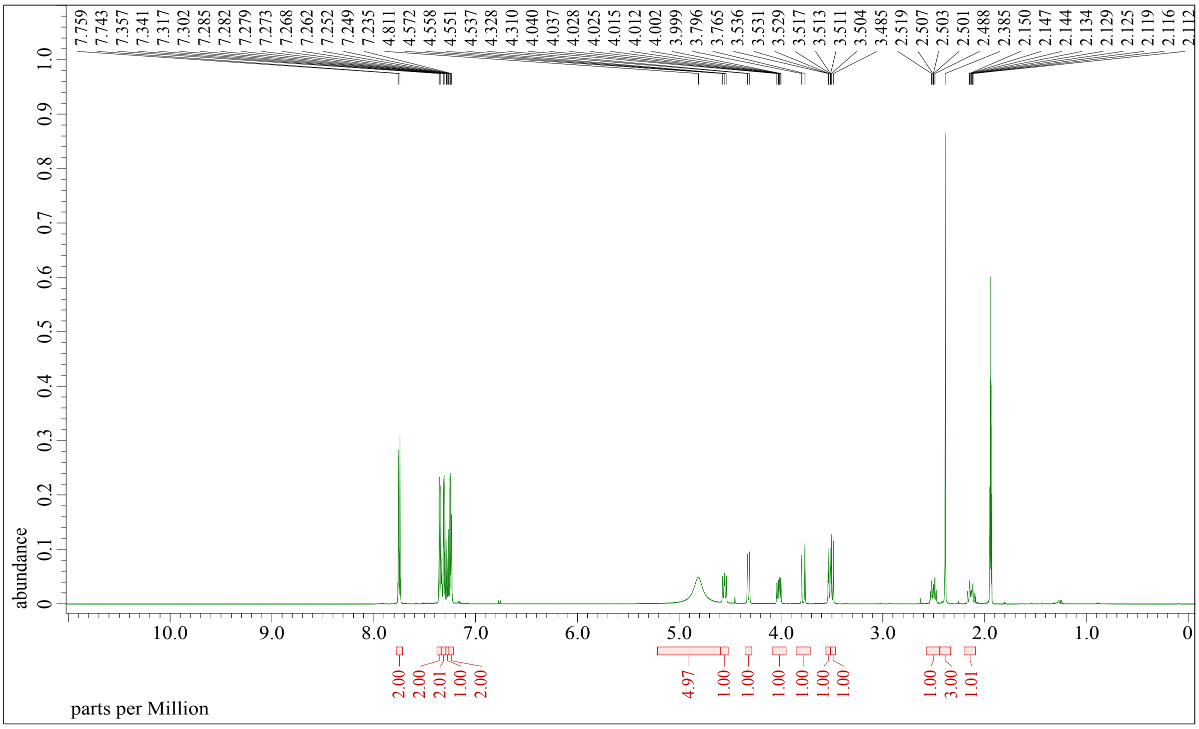

Figure S13. ^{1}H NMR spectrum of **6g** (500 MHz, MeCN-d_3). Note: The broad signal observed at 4.81 ppm represents ammonium ion and residual water.

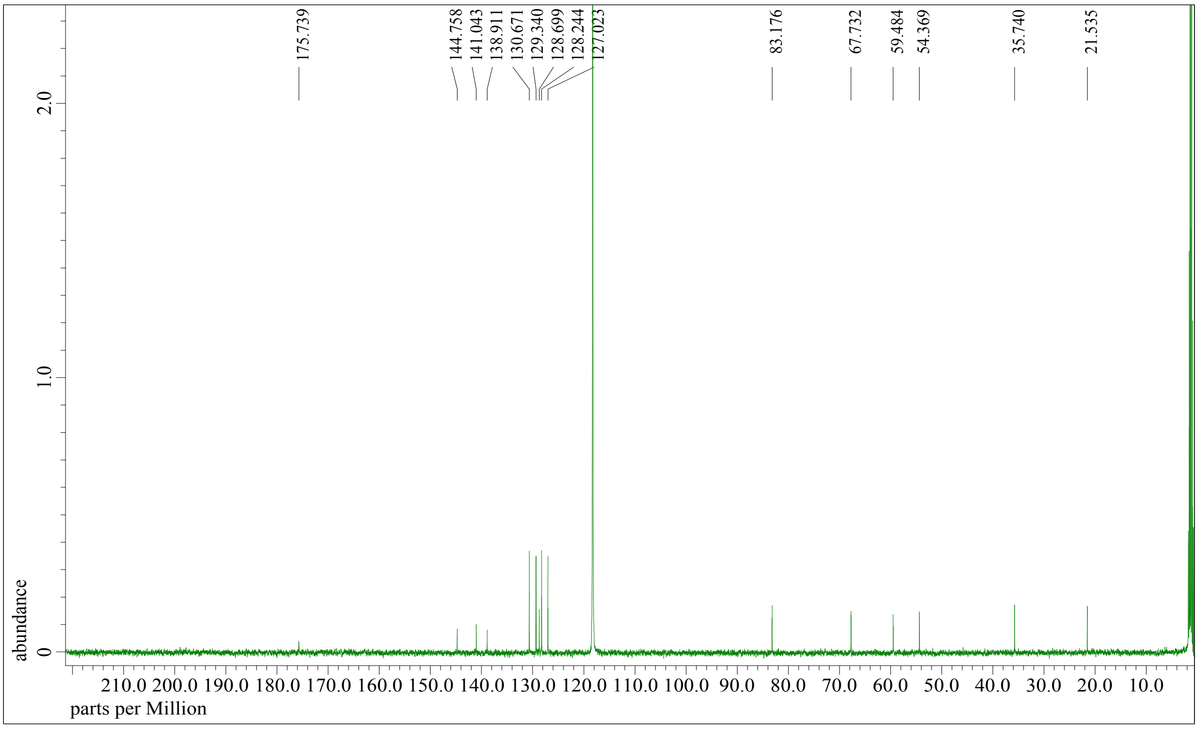

Figure S14. ^{13}C{^{1}H} NMR spectrum of **6g** (126 MHz, MeCN-d_3)

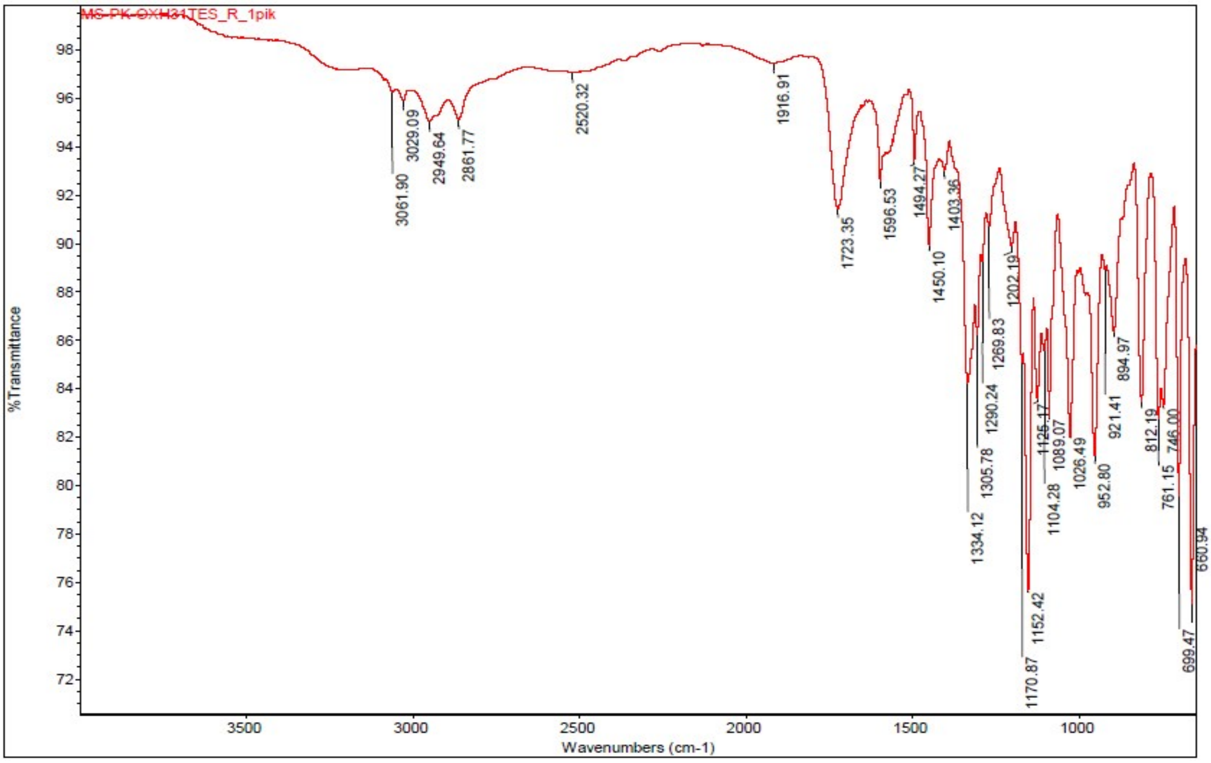

Figure S15. IR spectrum of **6g**

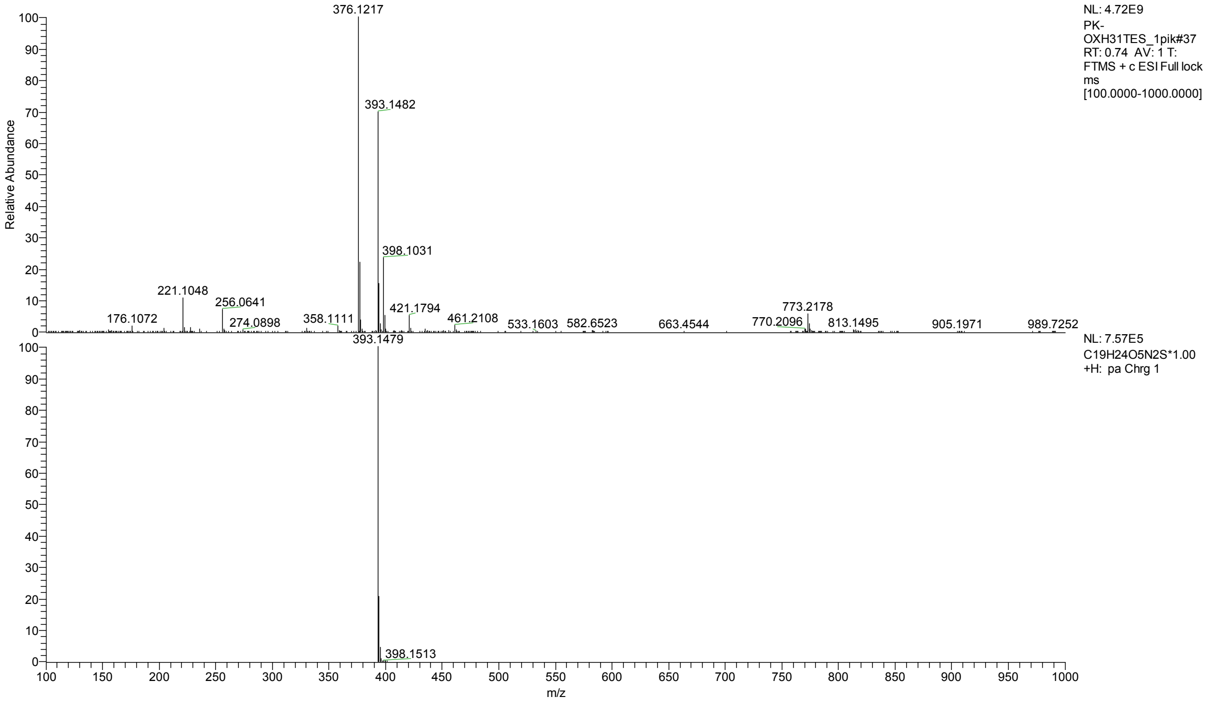

Figure S16. HRMS spectrum of **6g**

(-)-(2R,5S)-4-((2-aminophenyl)sulfonyl)-2-phenyl-1,4-oxazepane-5-carboxylic acid 7a

The analytical data are identical for **7a** prepared from Wang resin or Wang-piperazine resin.

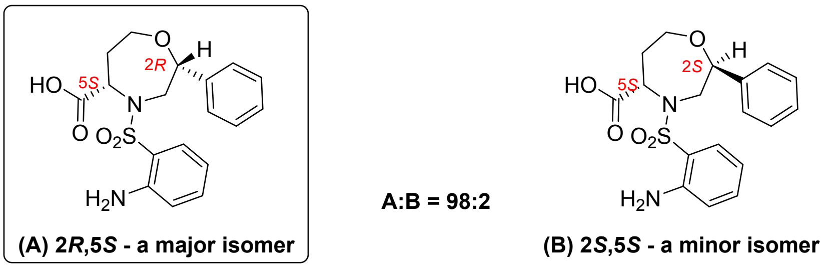

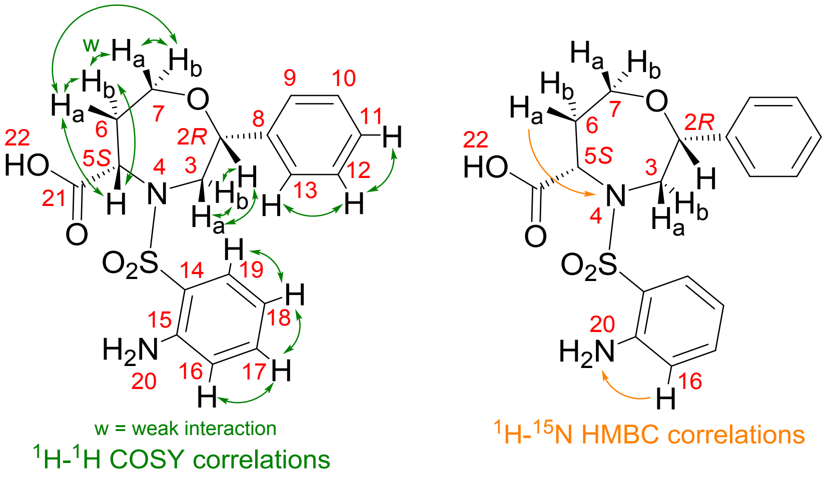

Figure S17. Detailed COSY and ^{1}H-^{15}N HMBC NMR analysis of 1,4-oxazepane **7a**

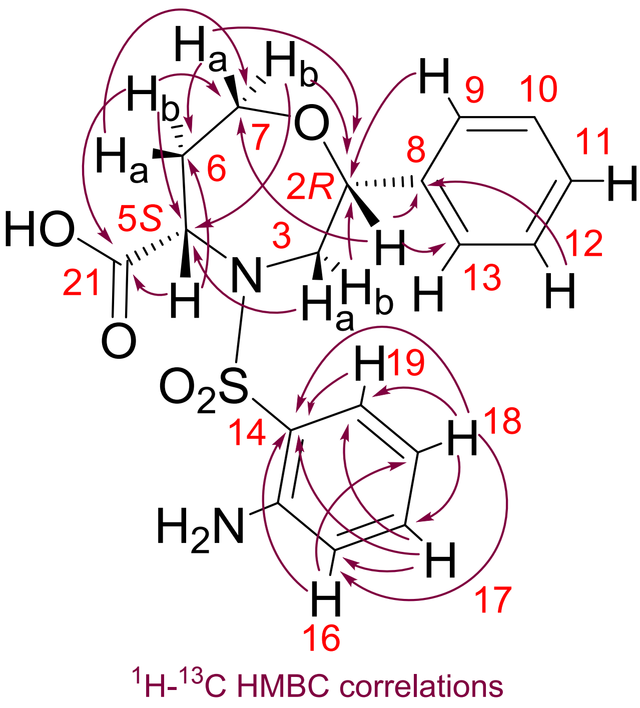

Figure S18. Detailed ^{1}H-^{13}C HMBC NMR analysis of 1,4-oxazepane **7a**

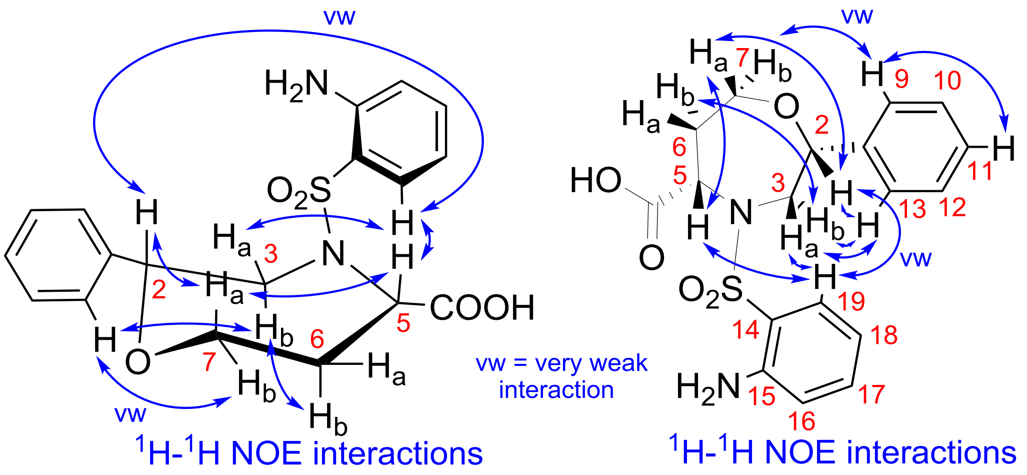

Figure S19. Detailed ^{1}H-^{1}H NOE NMR analysis of 1,4-oxazepane **7a**

Table S2. ^{1}H NMR (500 MHz) and ^{13}C{^{1}H} NMR (126 MHz) data, including detailed COSY, ^{1}H-^{13}C HMBC and NOE correlations for 1,4-oxazepane **7a**^{a}

**position**	**^{1}H NMR δ_H [ppm]^b**	**splitting pattern, *J* [Hz], integration**	**^{13}C{^{1}H} NMR δ_C [ppm]**	**COSY correlations**	**^{1}H-^{13}C HMBC correlations**	**NOE correlations**
**2**	4.16	d, *J* = 9.5 Hz, 1H	82.7	H_b^3	67.5, 127.1, 141.0	H_a^7, $H^{9,13}$, H^{19} (weak int.)
**3**	H_a : 3.70	d, *J* = 16.1 Hz, 1H,	54.1	H_b^3	59.1	$H^{9,13}$, H^{19}
	H_b: 3.50	dd, *J* = 16.1, 9.5 Hz, 1H		H^2, H_a^3	59.1 (weak int.), 82.7	H_b^6, $H^{9,13}$
**5**	4.66	dd, *J* = 10.4, 7.1 Hz, 1H	59.1	H_a^6, H_b^6	35.6, 174.8	H_a^7, H^{19}
**6**	H_a : 2.54	ddd, *J* = 15.4, 7.1, 6.3 Hz, 1H	35.6	H^5, H_b^6, H_b^7	67.5	-
	H_b: 2.16	dddd, *J* = 15.4, 10.4, 8.9, 1.1 Hz, 1H		H^5, H_a^6, H_a^7	59.1, 67.5, 174.8	H_b^3
**7**	H_a : 3.65	dd, *J* = 12.7, 8.9 Hz, 1H	67.5	H_b^6, H_b^7	35.6, 59.1, 82.7	H^2, H^5,
	H_b: 4.02	ddd, *J* = 12.7, 6.3, 1.1 Hz, 1H		H_a^6, H_a^7	59.1, 82.7	$H^{9,13}$ (very weak int.)
**8**	-	-	141.0	-	-	
**9,13**	7.15	m, 2H	127.1	$H^{10,12}$	82.7, 128.7, 129.3	H^2, H_a^3, H_b^3, H_b^7 (very weak int.)
**10,12**	7.27-7.31	m, 2H	129.3	$H^{9,13}$, H^{11}	127.1, 128.7, 141.0	-
**11**	7.23-7.27	m, 1H	128.7	$H^{10,12}$	127.1, 129.3	-
**14**	-	-	121.9	-	-	-
**15**	-	-	147.5	-	-	-
**16**	6.84	dd, *J* = 8.3, 1.0 Hz, 1H	118.6	H^{17}	117.7, 121.9	-
**17**	7.34	ddd, *J* = 8.3, 7.2, 1.5 Hz, 1H	135.4	H^{16}, H^{18},	118.6, 131.0, 147.5	-
**18**	6.75	ddd, *J* = 8.1, 7.2, 1.0 Hz, 1H	117.7	H^{17}, H^{19}	121.9, 131.0, 135.4	-
**19**	7.66	dd, *J* = 8.1, 1.5 Hz, 1H	131.0	H^{18}	135.4, 147.5	H^2 (very weak int.), H_a^3, H^5
**21**	-	-	174.8	-	-	-

^{a}Assignments are based on extensive 1D and 2D NMR analysis (^{1}H-^{1}H COSY, ^{1}H-^{13}C HMQC, ^{1}H-^{13}C HMBC and ^{1}H-^{1}H NOESY); measured in MeCN-d_3; int. = interaction.

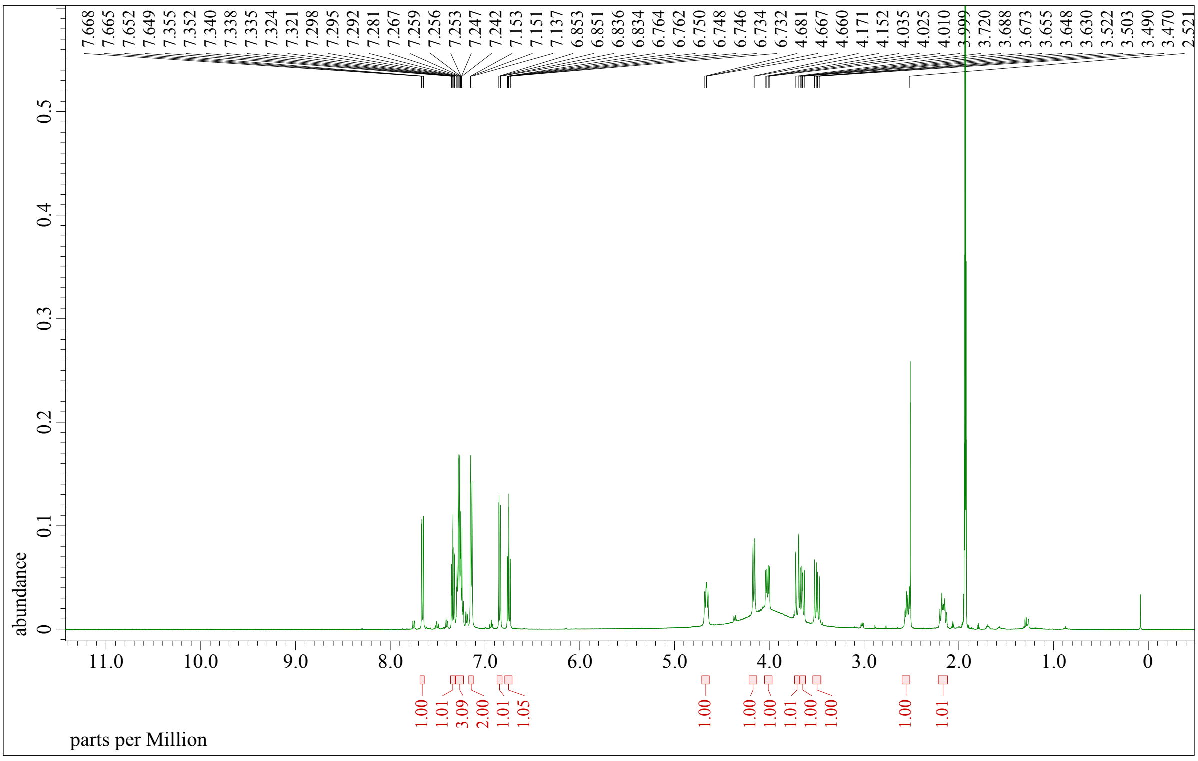

Figure S20. ^{1}H NMR spectrum of **7a** (500 MHz, MeCN-d_3)

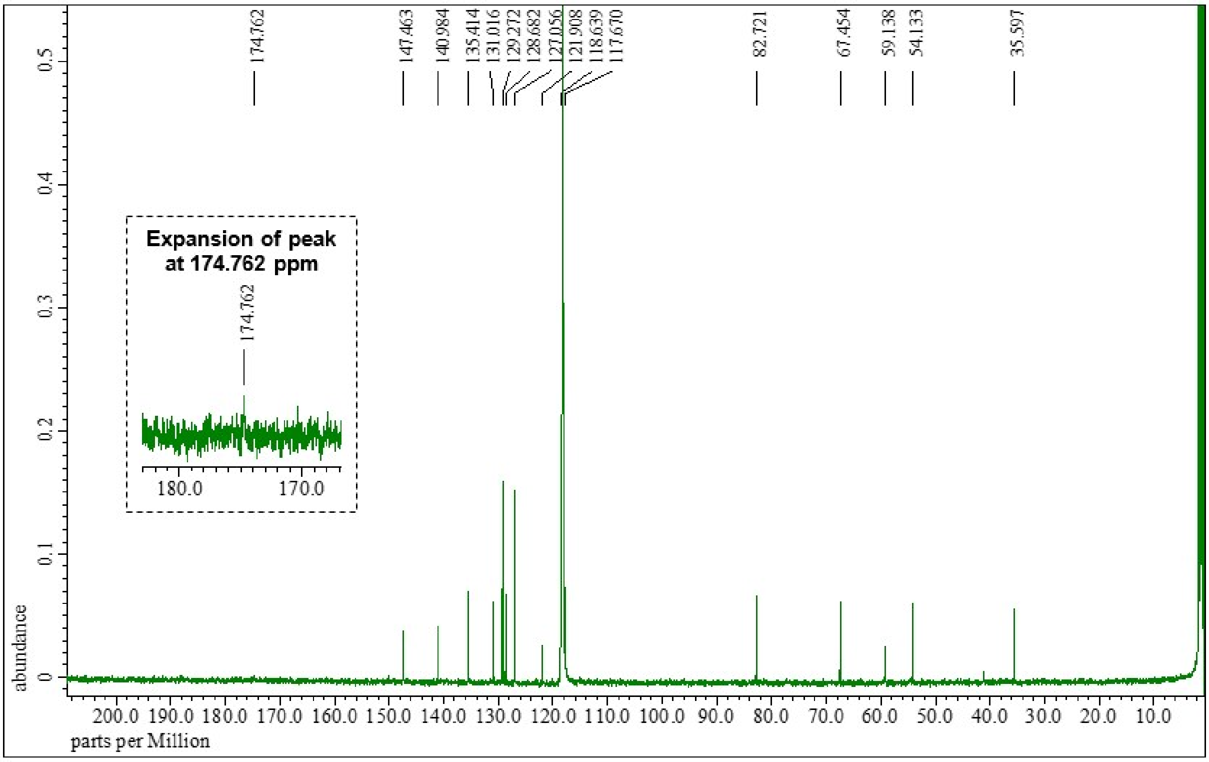

Figure S21. $^{13}C\{^{1}H\}$ NMR spectrum of **7a** (126 MHz, MeCN-d_3)

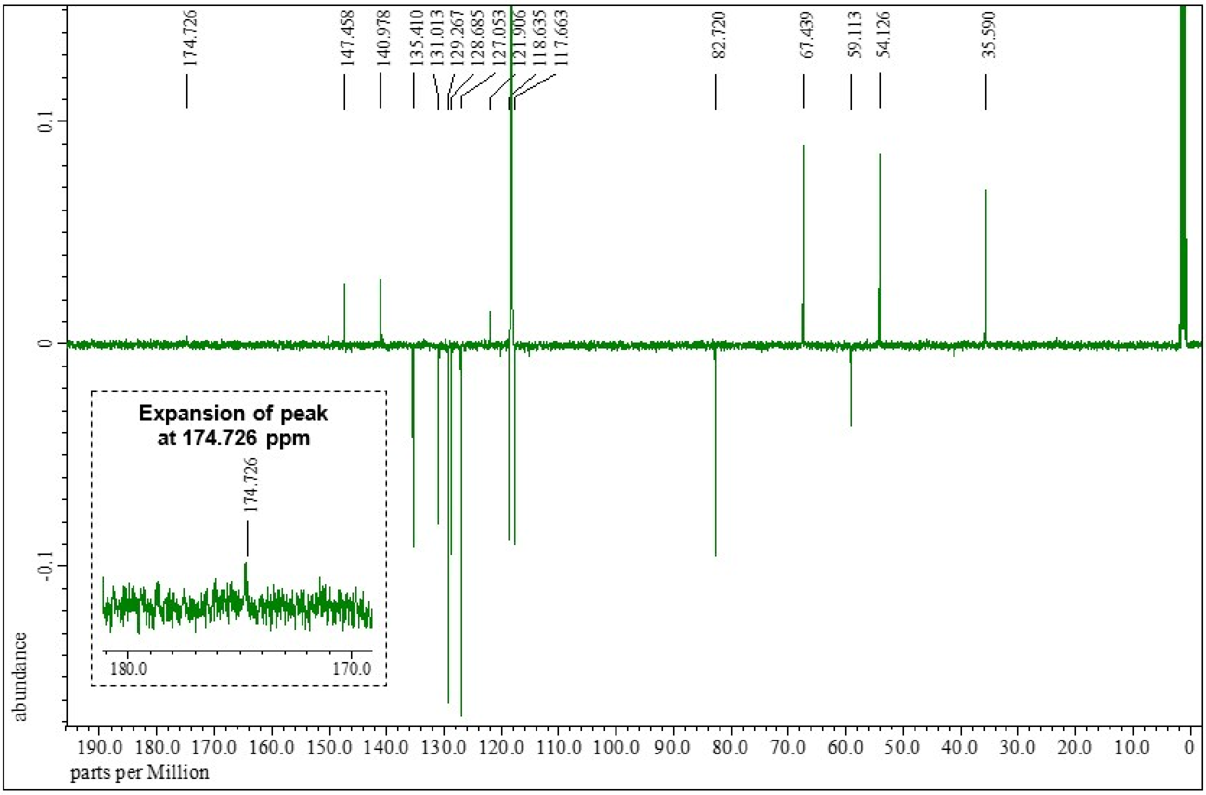

Figure S22. ^{13}C APT NMR spectrum of **7a** (126 MHz, MeCN-d_3)

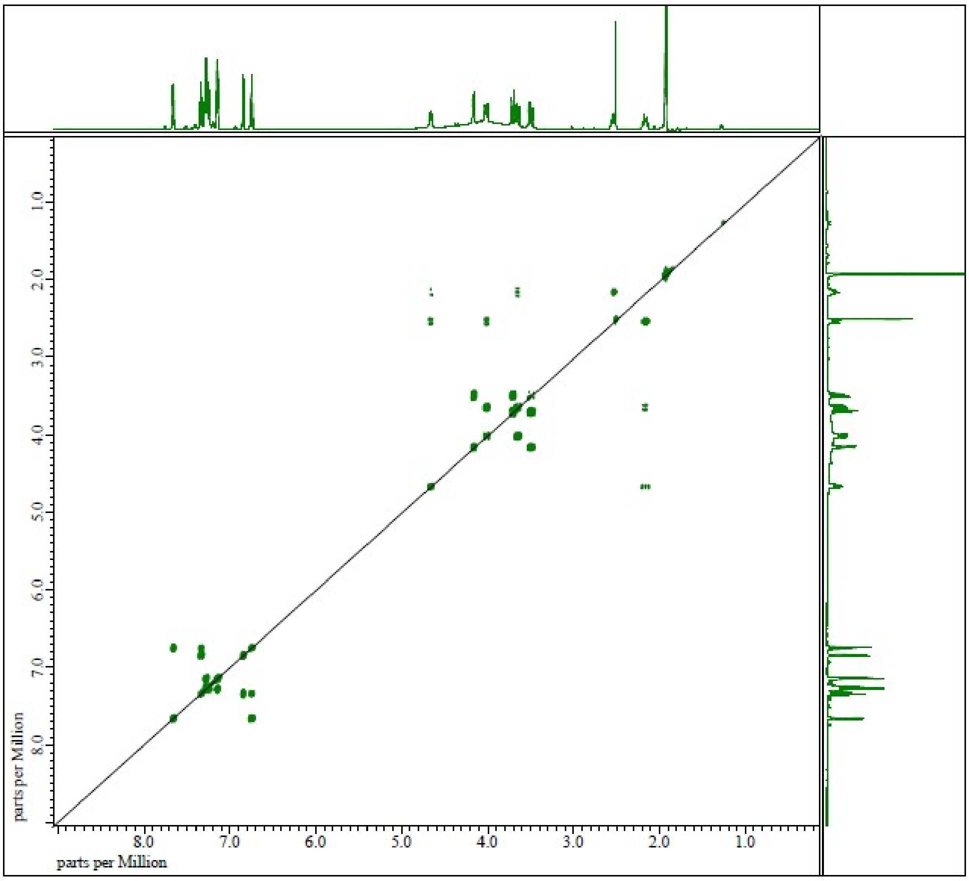

Figure S23. ^{1}H-^{1}H COSY NMR spectrum of **7a** (500 MHz, MeCN-d_3)

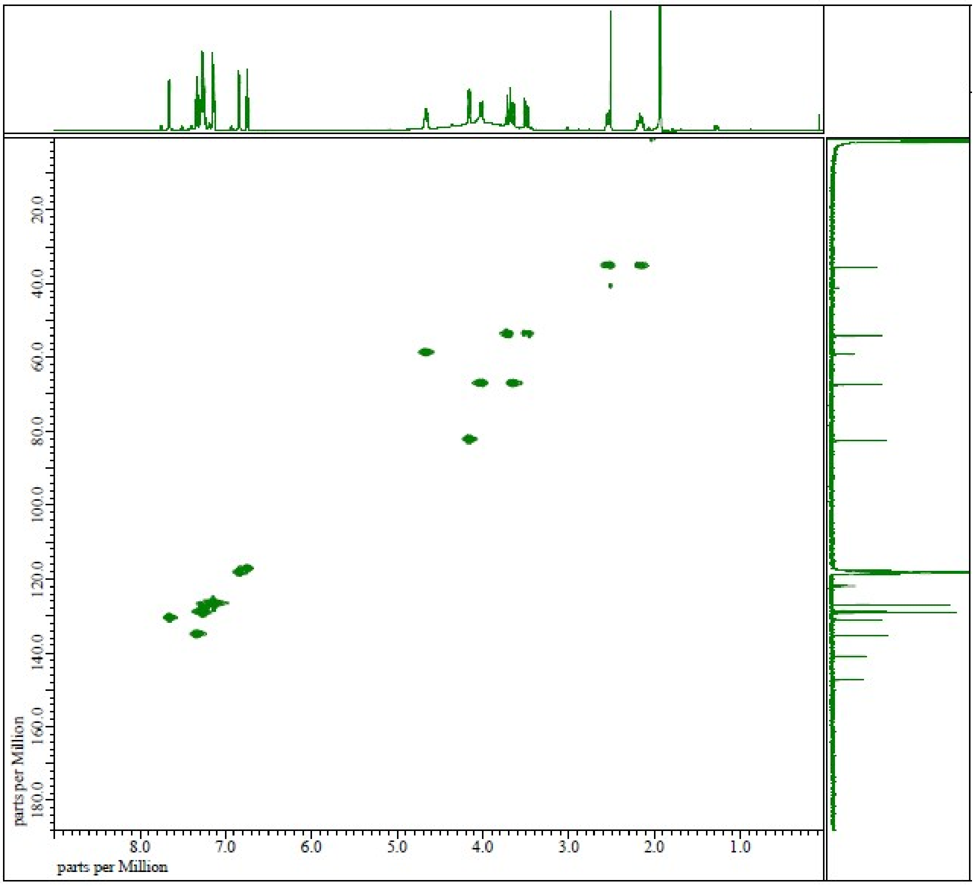

Figure S24. ^1H-^{13}C HMQC NMR spectrum of **7a** (MeCN-d_3)

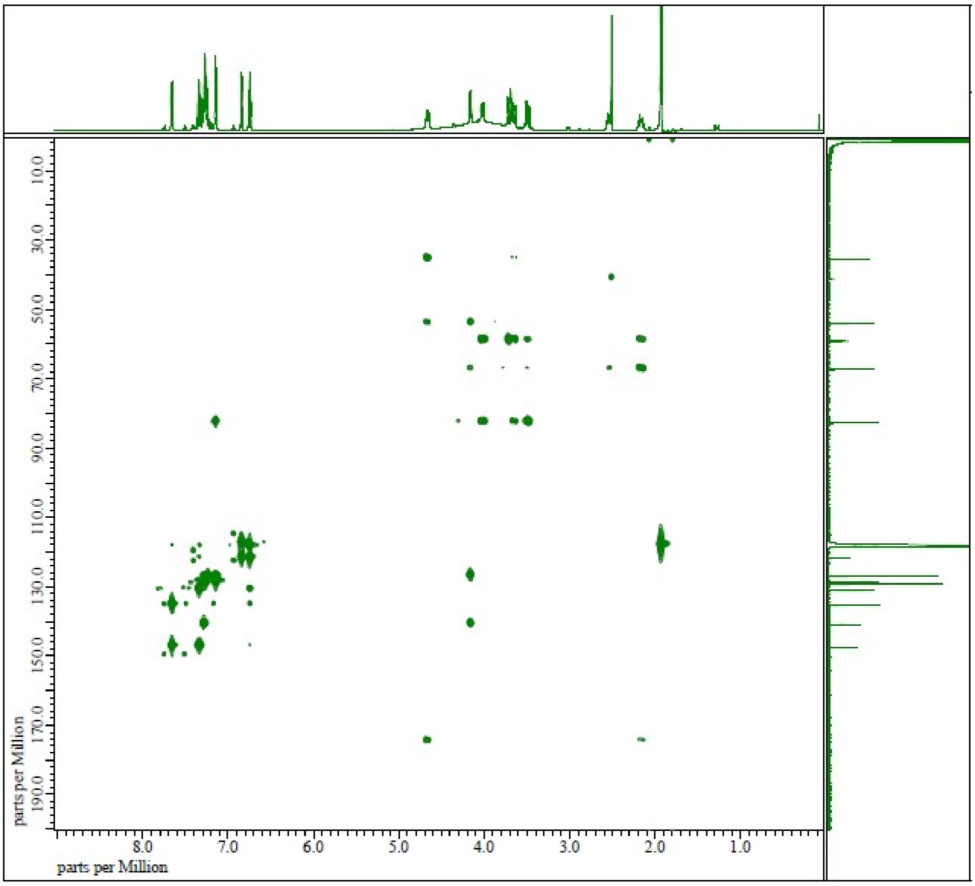

Figure S25. ^1H-^{13}C HMBC NMR spectrum of **7a** (MeCN-d_3)

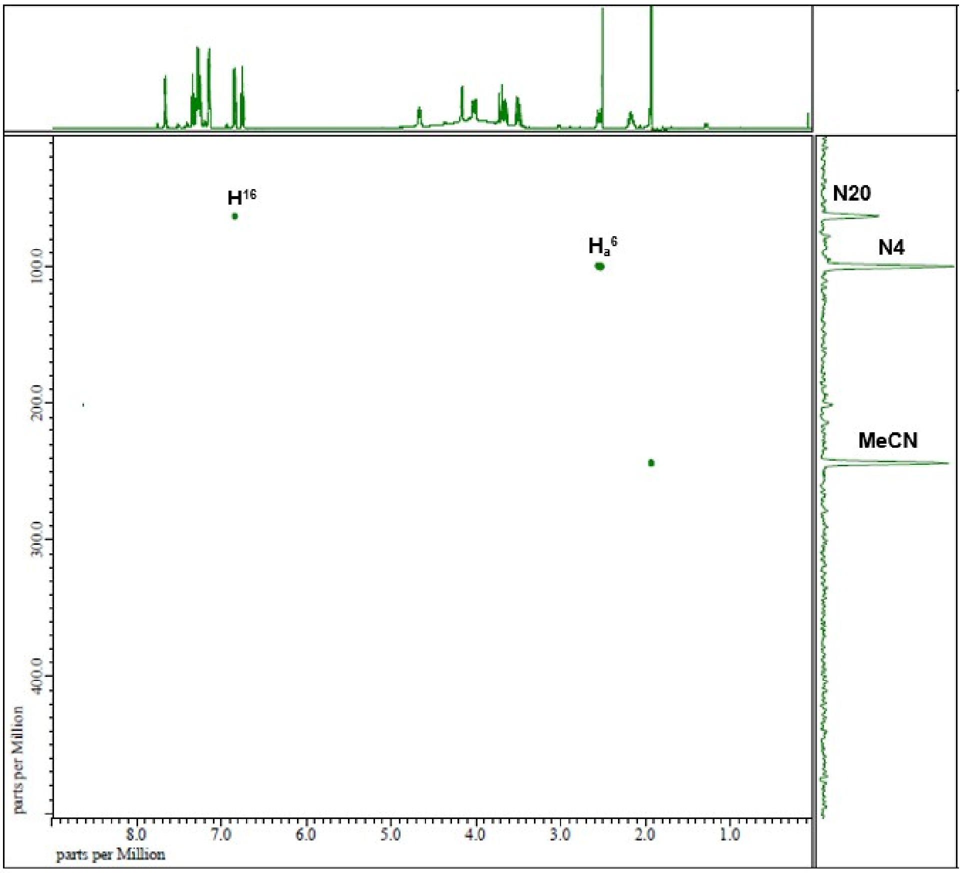

Figure S26. ^{1}H-^{15}N HMBC NMR spectrum of **7a** (MeCN-d_3)

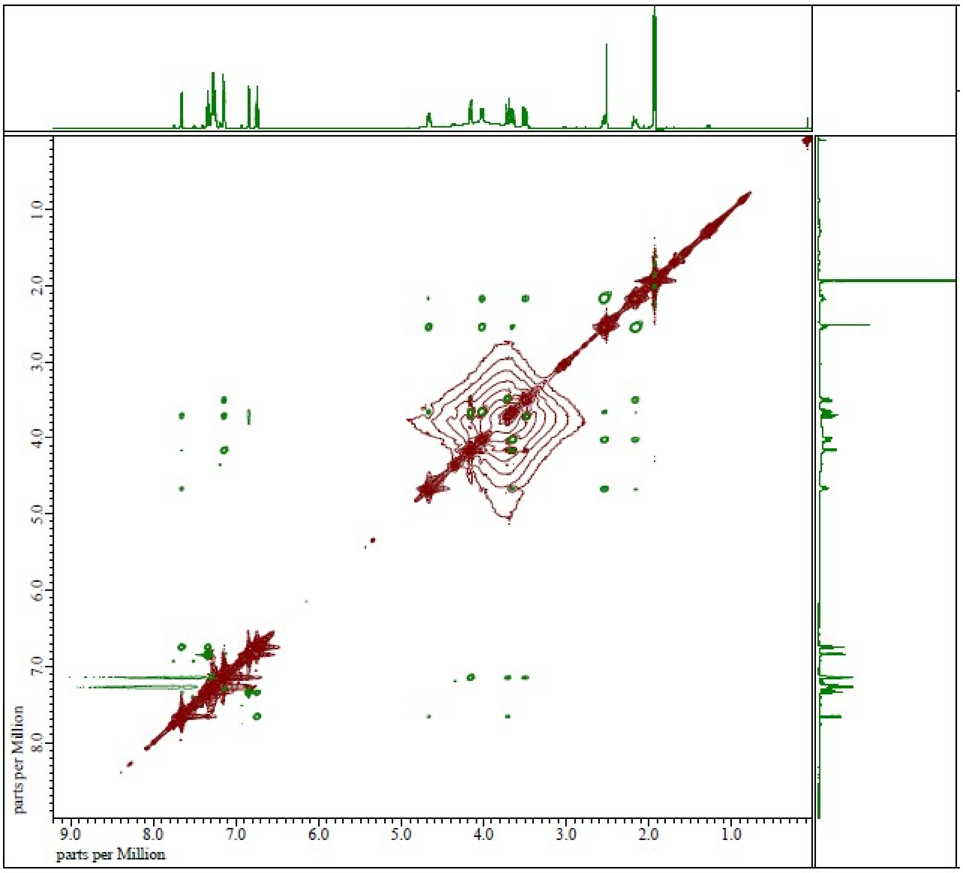

Figure S27. ^{1}H-^{1}H NOESY NMR spectrum of **7a** (MeCN-d_3)

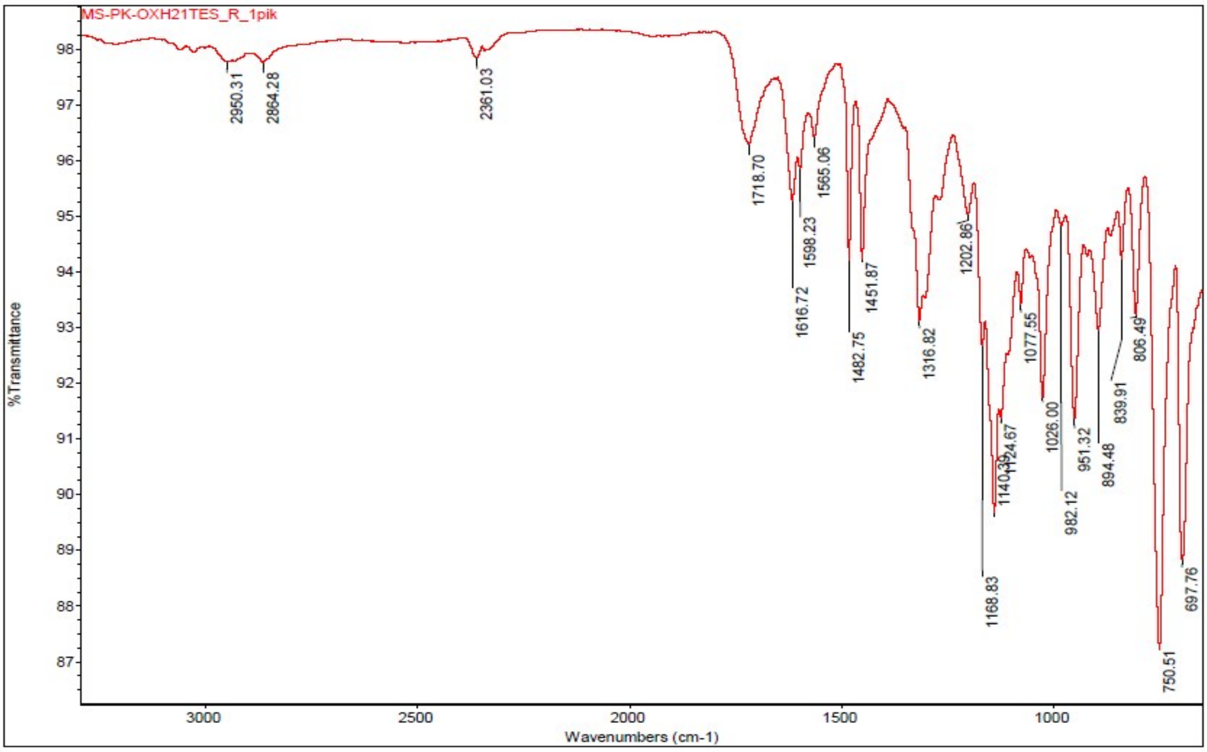

Figure S28. IR spectrum of **7a**

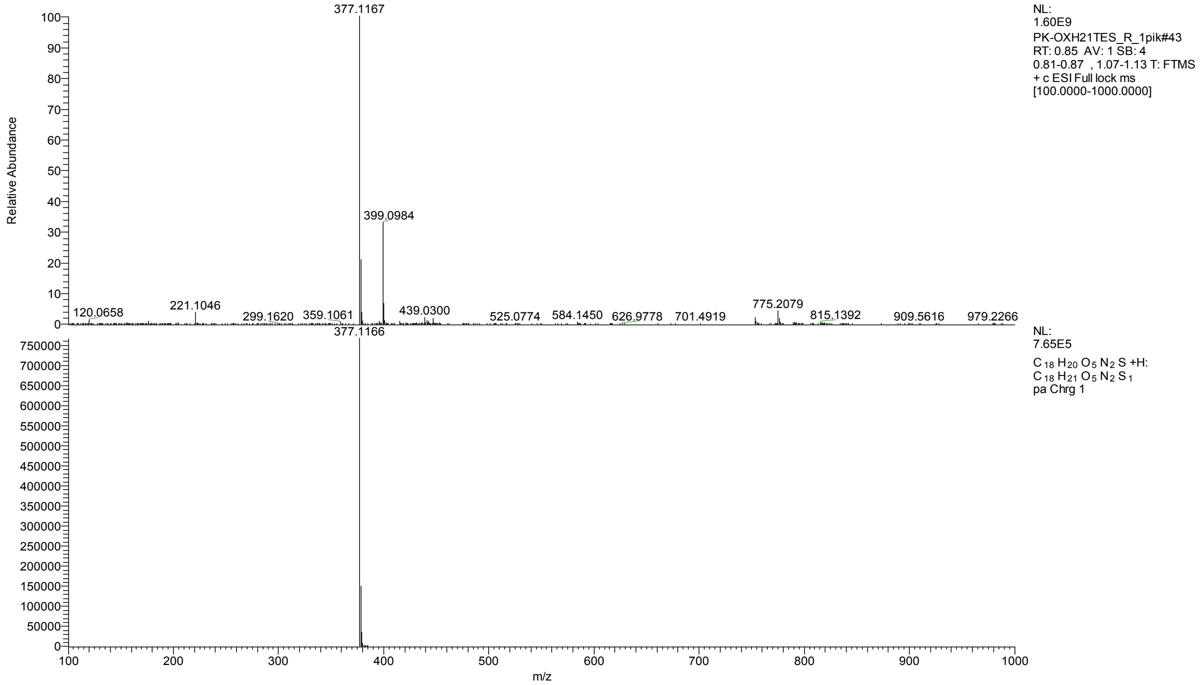

Figure S29. HRMS spectrum of **7a**

(-)-(2*R*,5S)-4-((2-aminophenyl)sulfonyl)-2-phenyl-1,4-oxazepane-5-carboxamide 7b^{2R}

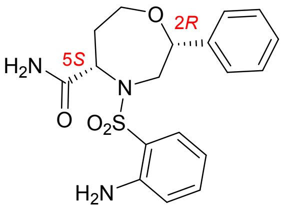

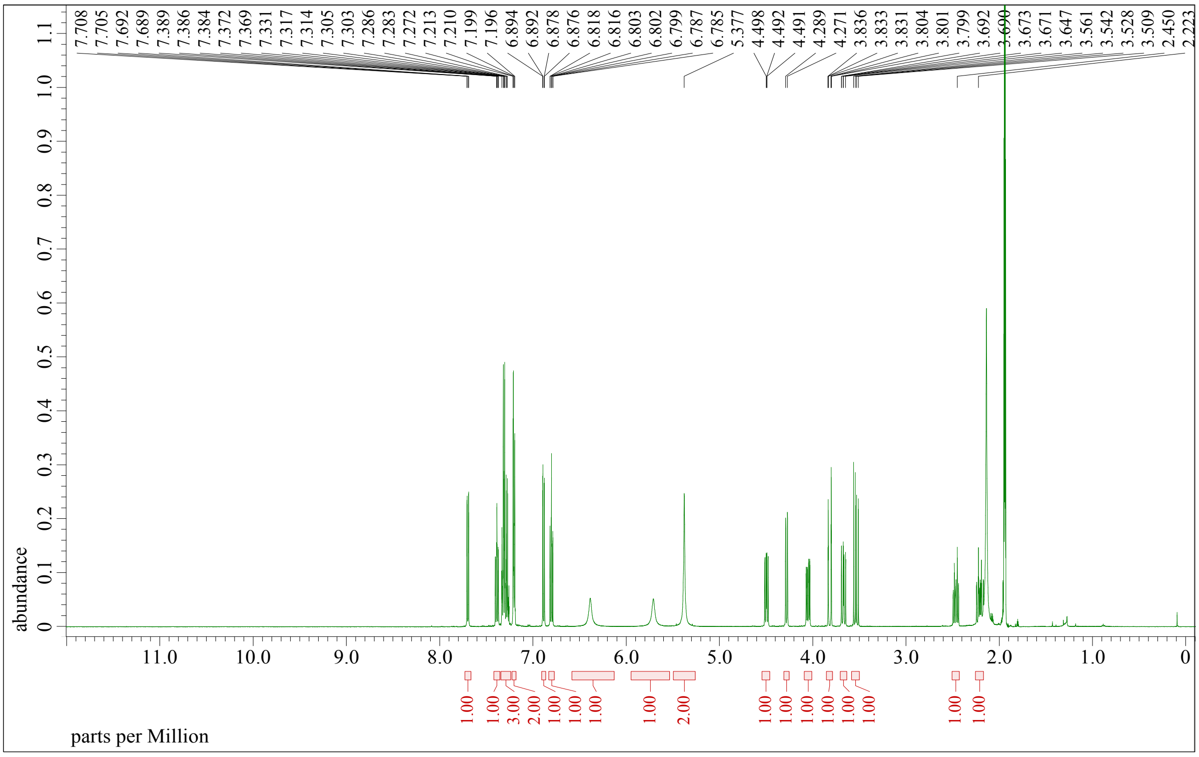

Figure S30. ^{1}H NMR spectrum of **7b^{2R}** (500 MHz, MeCN-d_3)

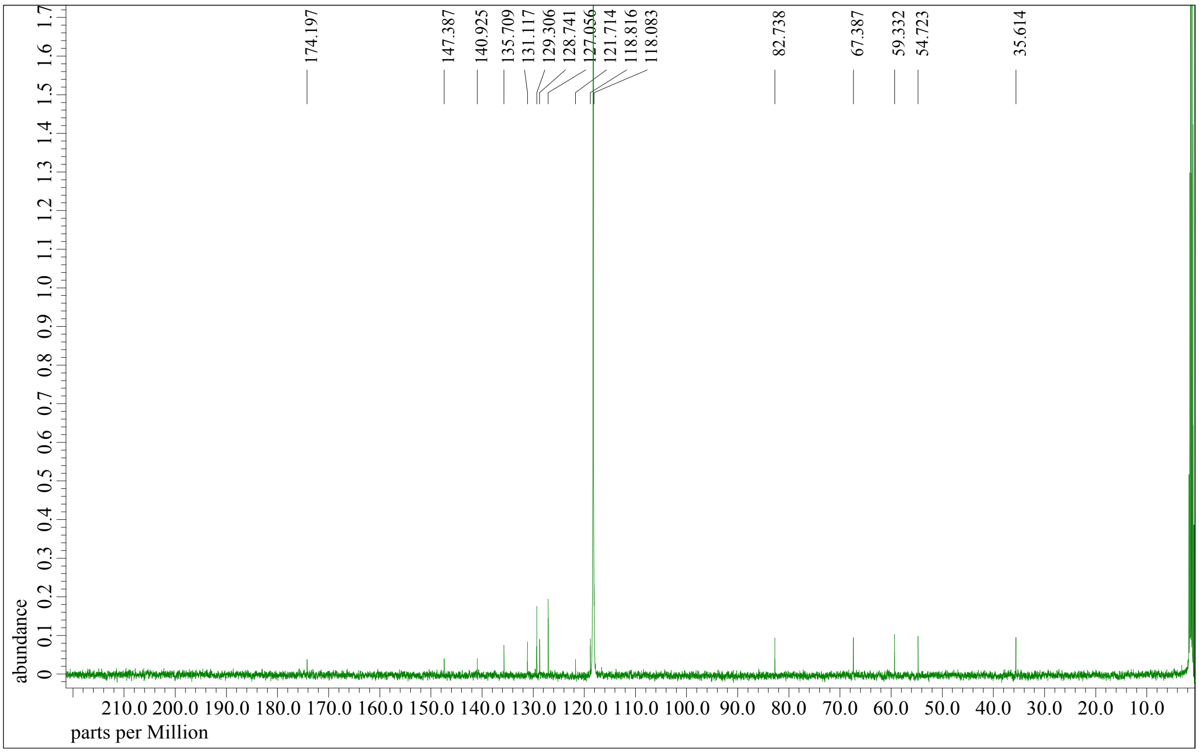

Figure S31. ^{13}C{^{1}H} NMR spectrum of **7b^{2R}** (126 MHz, MeCN-d_3)

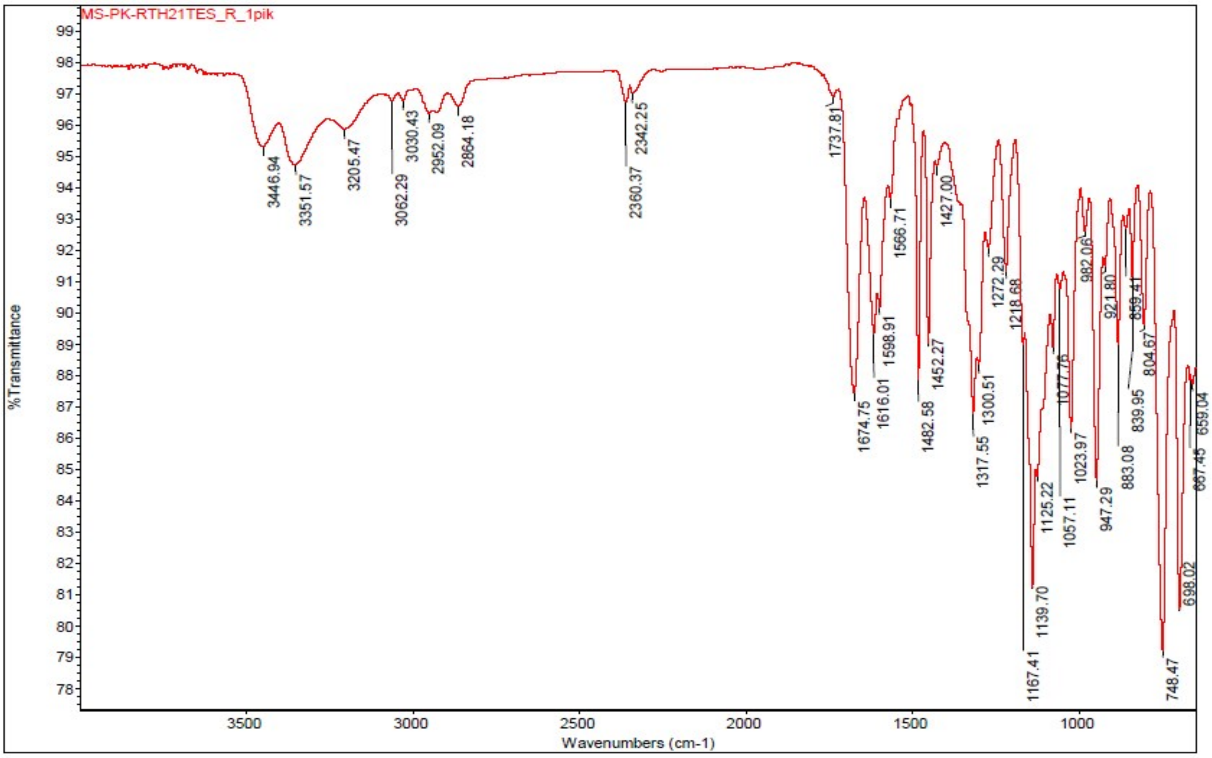

Figure S32. IR spectrum of **7b**$^{\boldsymbol{2R}}$

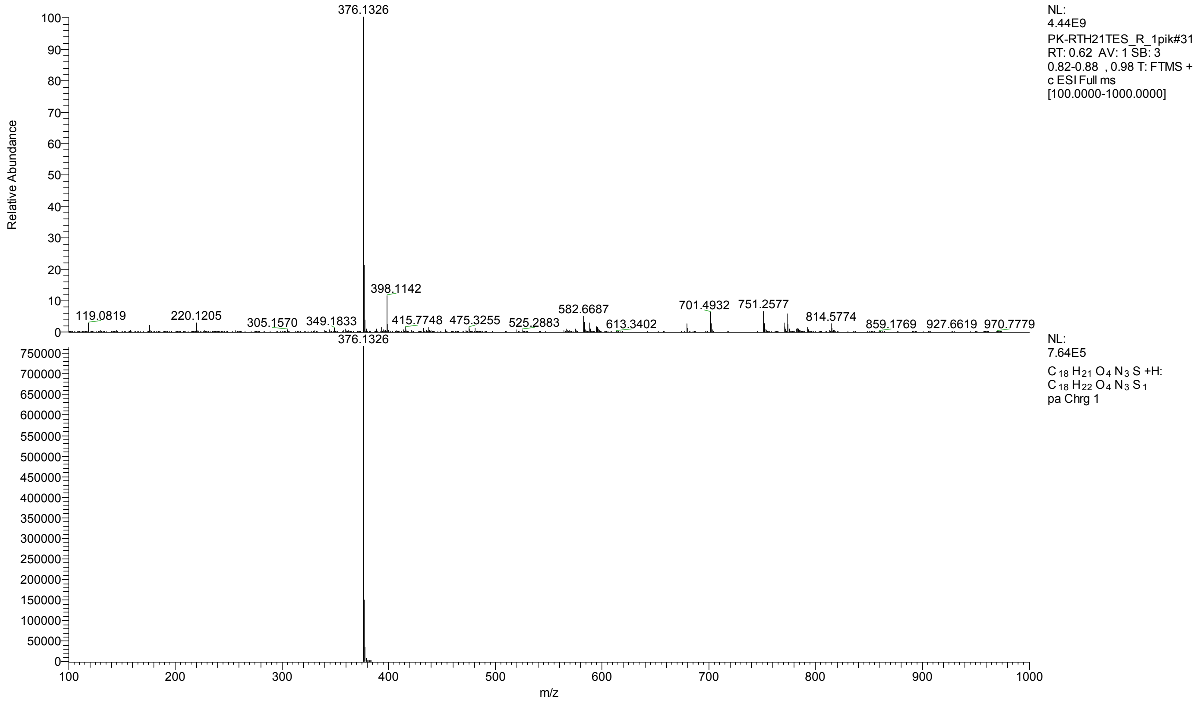

Figure S33. HRMS spectrum of **7b**$^{\boldsymbol{2R}}$

(+)-(2*S*,5S)-4-((2-aminophenyl)sulfonyl)-2-phenyl-1,4-oxazepane-5-carboxamide 7b*2S*

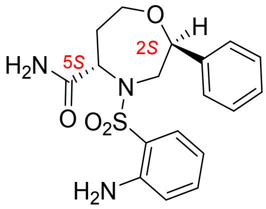

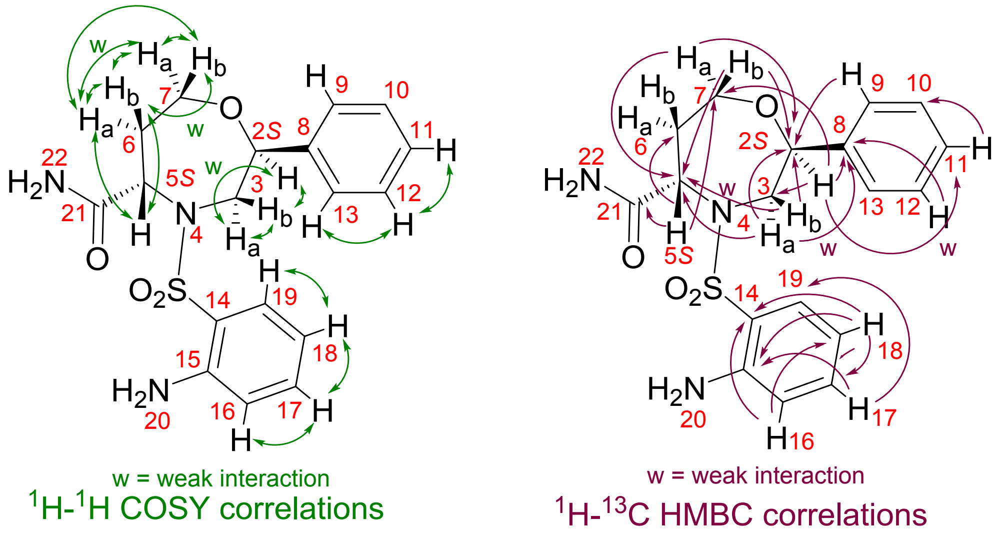

Figure S34. Detailed COSY and ^{1}H-^{13}C HMBC NMR analysis of 1,4-oxazepane **7b*2S***

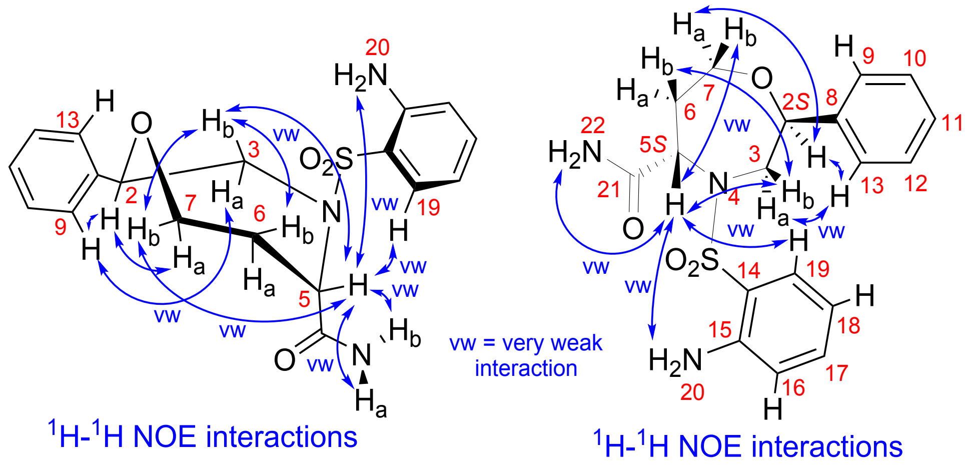

Figure S35. Detailed ^{1}H-^{1}H NOE NMR analysis of 1,4-oxazepane **7b*2S***

Table S3. ^{1}H NMR (500 MHz) and ^{13}C{^{1}H} NMR (126 MHz) spectral data, including detailed COSY, ^{1}H-^{13}C HMBC and NOE correlations for 1,4-oxazepane **7b**$^{2S\,a}$

position	^{1}H NMR δ_H [ppm]^b	splitting pattern, *J* [Hz], integration	^{13}C{^{1}H} NMR δ_C [ppm]	COSY correlations	^{1}H-^{13}C HMBC correlations	NOE correlations
**2**	4.64	dd, *J* = 8.7, 1.6 Hz, 1H	81.4	H_a^3, H_b^3	56.8, 126.6, 128.6 (weak int.), 141.9	H_a^7, $H^{9,13}$
**3**	H_a : 3.63	dd, *J* = 14.2, 1.6 Hz, 1H,	56.8	H^2, H_b^3	59.4, 81.4	$H^{9,13}$ (very weak int.)
	H_b: 3.43	dd, *J* = 14.2, 8.7 Hz, 1H		H^2, H_a^3	59.4 (weak int.), 81.4, 141.9 (weak int.)	H^5, H_b^6
**5**	4.59	dd, *J* = 4.5, 4.5 Hz, 1H	59.4	H_a^6, H_b^6	33.0 (weak int.), 66.9, 174.3	H_b^3, H_b^7 (very weak int.), H^{19} (very weak int.), H^{20}, H^{22} (very weak int.)
**6**	H_a : 2.30	dddd, *J* = 15.8, 4.9, 4.5, 1.6 Hz, 1H	33.0	H^5, H_b^6, H_a^7 (weak int.), H_b^7	-	-
	H_b: 2.10	dddd, *J* = 15.8, 11.0, 4.5, 3.0 Hz, 1H		H^5, H_a^6, H_a^7, H_b^7 (weak int.)	-	H_b^3
**7**	H_a : 3.78	ddd, *J* = 12.8, 11.0, 1.6 Hz, 1H	67.0	H_a^6 (weak int.), H_b^6, H_b^7	59.4, 81.4	H^2
	H_b: 3.94	ddd, *J* = 12.8, 4.5, 3.0 Hz, 1H		H_a^6, H_b^6 (weak int.), H_a^7	59.4, 81.4	H^5 (very weak int.)
**8**	-	-	141.9	-	-	-
**9,13**	7.18-7.19	m, 2H	126.6	$H^{10,12}$	81.4, 128.6	H^2, H_a^3 (weak int.)
**10,12**	7.27-7.30	m, 2H	129.3	$H^{9,13}$, H^{11}	141.9	-
**11**	7.24-7.27	m, 1H	128.6	$H^{10,12}$	126.6, 129.3	-
**14**	-	-	119.1	-	-	-
**15**	-	-	148.0	-	-	-
**16**	6.85	dd, *J* = 8.5, 1.1 Hz, 1H	118.7	H^{17}	117.5, 119.1	-
**17**	7.35	ddd, *J* = 8.5, 7.2, 1.6 Hz, 1H	135.7	H^{16}, H^{18},	131.3, 148.0	-
**18**	6.70	ddd, *J* = 8.2, 7.2, 1.1 Hz, 1H	117.5	H^{17}, H^{19}	118.7, 119.1, 148.0	-
**19**	7.58	dd, *J* = 8.1, 1.6 Hz, 1H	131.3	H^{18}	135.7	H^5 (very weak int.)
**20**	5.62	br. s, 2H	-	-	-	H^5
**21**	-	-	174.4	-	-	-
**22**	H_a : 5.99	br. s, 1H	-	-	-	H^5 (very weak int.)
	H_b: 6.75	br.s, 1H				H^5 (very weak int.)

^{a}Assignments are based on extensive 1D and 2D NMR analysis (^{1}H-^{1}H COSY, ^{1}H-^{13}C HMQC, ^{1}H-^{13}C HMBC and ^{1}H-^{1}H NOESY); measured in MeCN-d_3; int. = interaction.

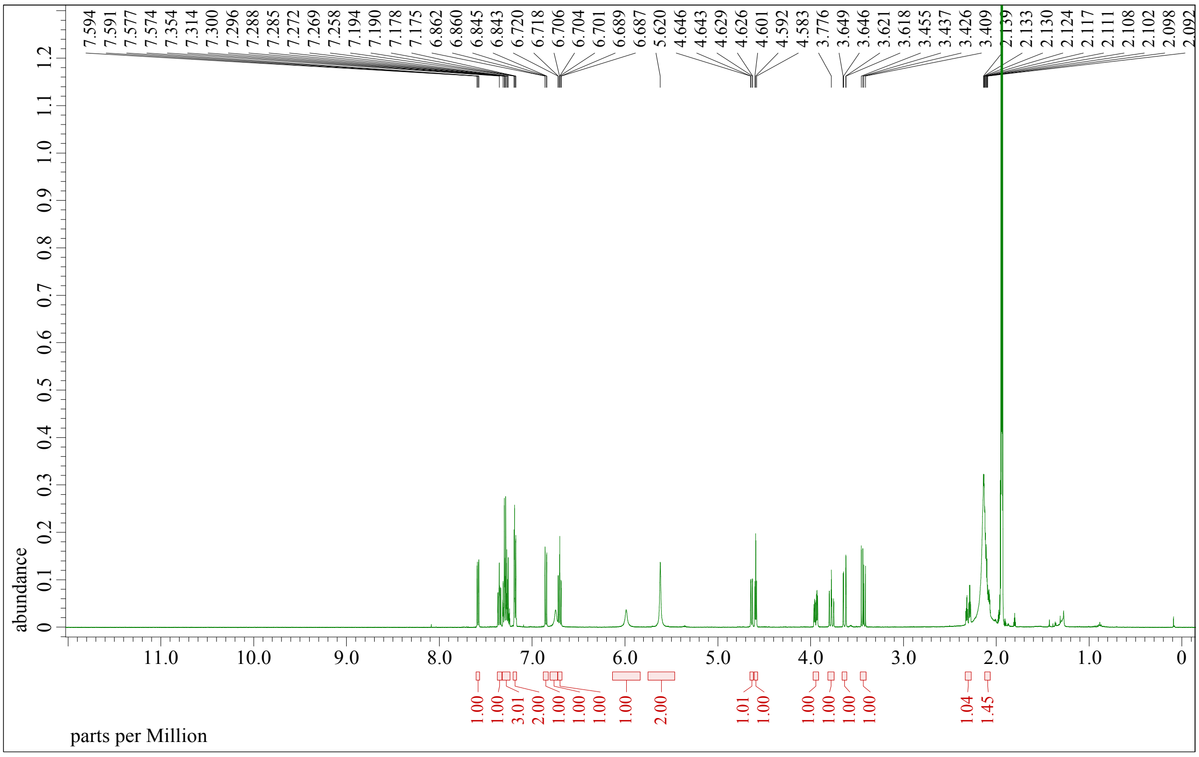

Figure S36. ^{1}H NMR spectrum of **7b^{2S}** (500 MHz, MeCN-d_3)

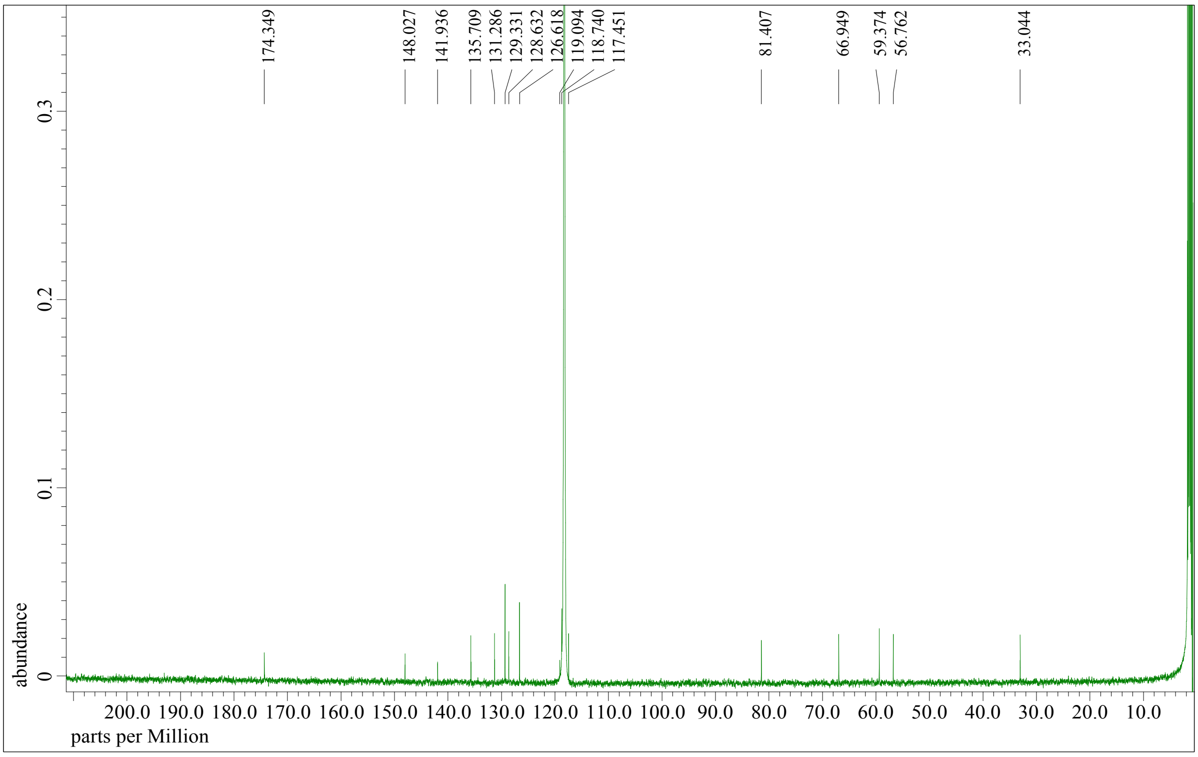

Figure S37. $^{13}C\{^{1}H\}$ NMR spectrum of **7b^{2S}** (126 MHz, MeCN-d_3)

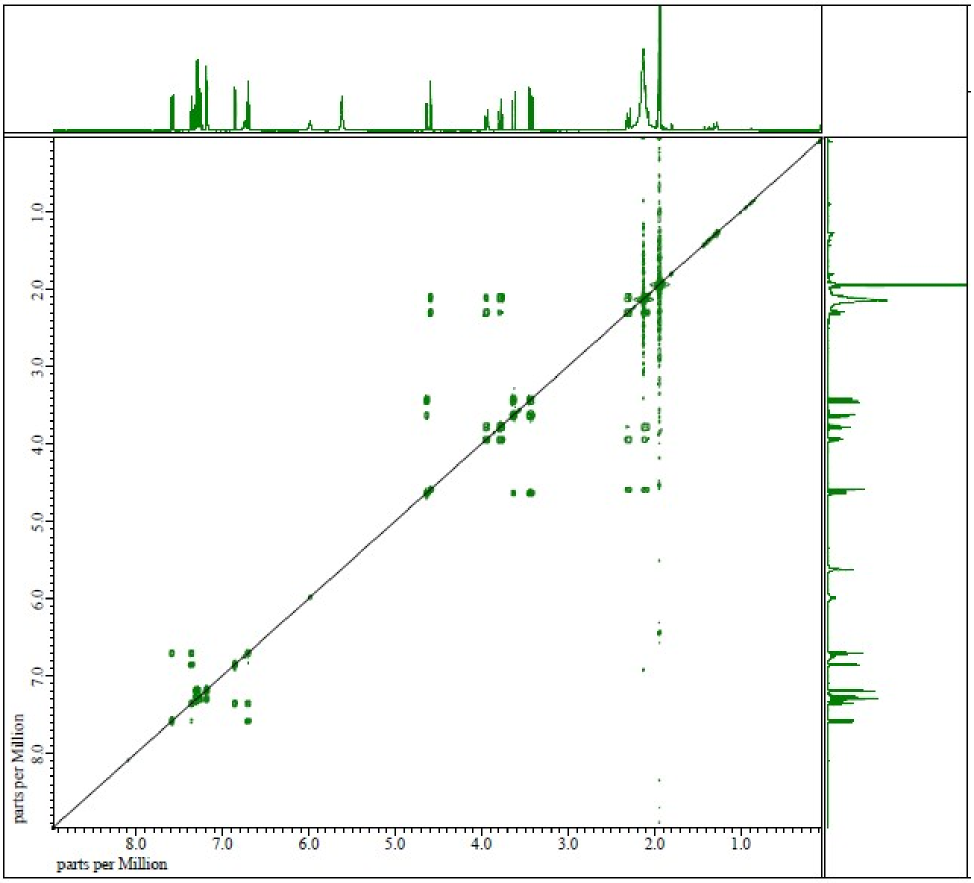

Figure S38. ^{1}H-^{1}H COSY NMR spectrum of **7b^{2S}** (500 MHz, MeCN-d_3)

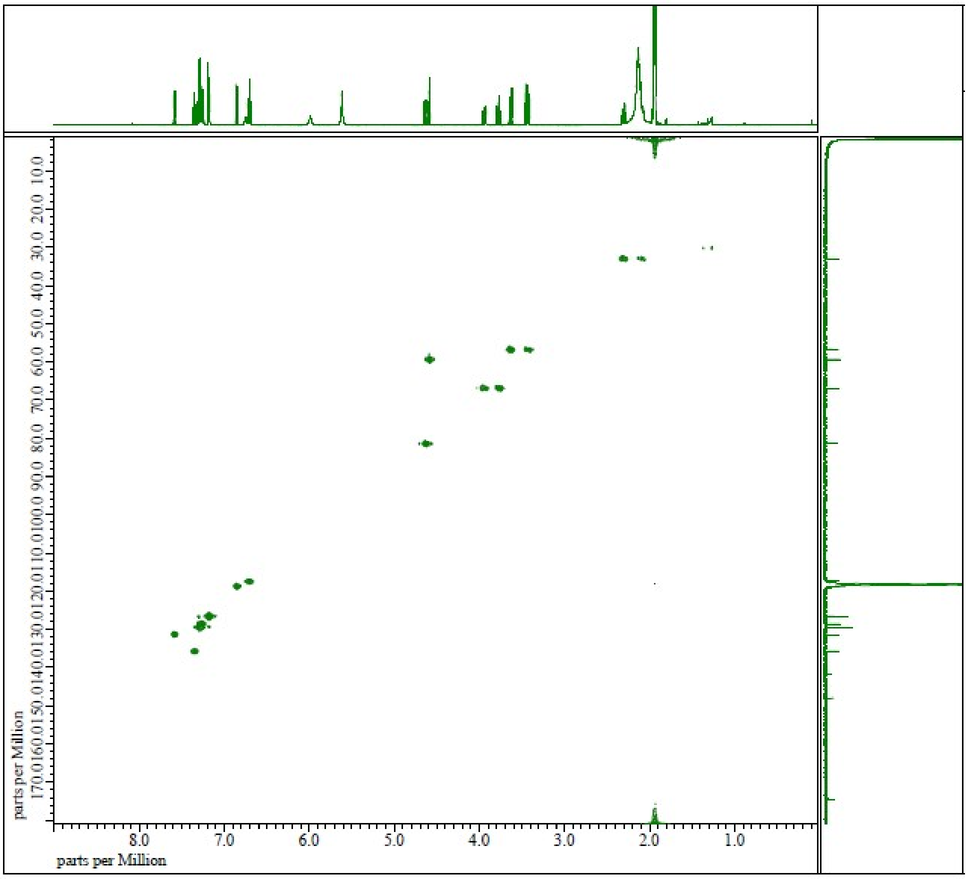

Figure S39. ^{1}H-^{13}C HMQC NMR spectrum of **7b^{2S}** (MeCN-d_3)

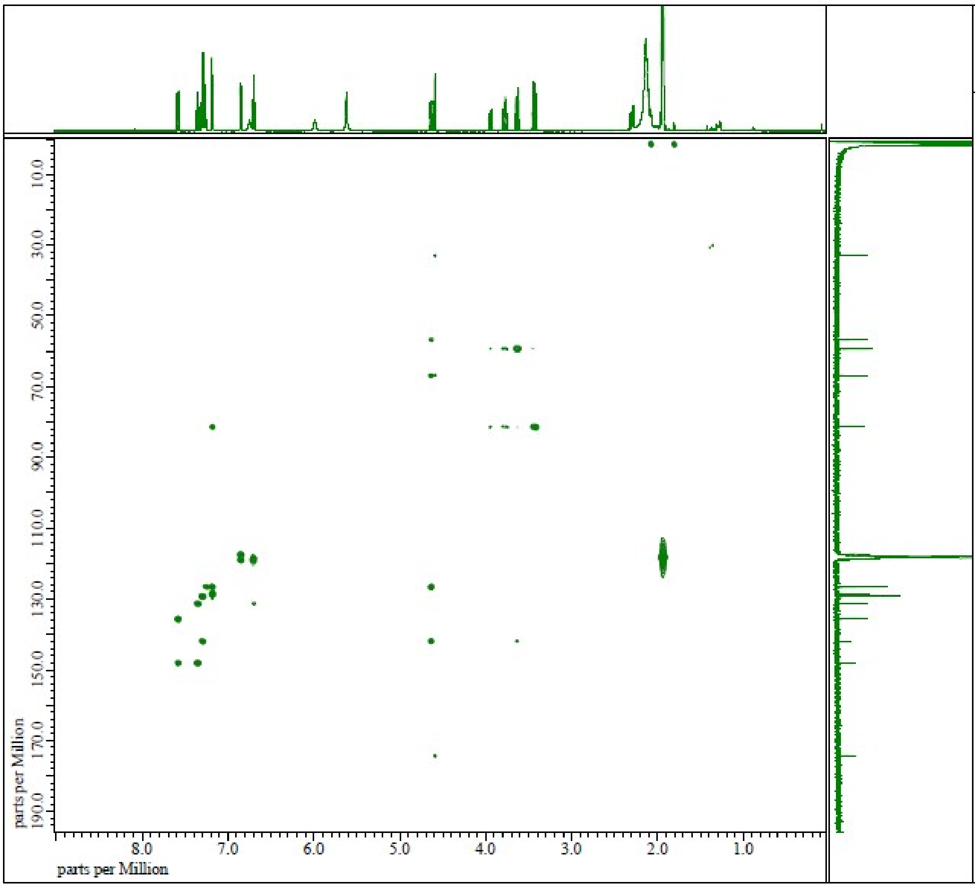

Figure S40. ^{1}H-^{13}C HMBC NMR spectrum of **7b^{2S}** (MeCN-d_3)

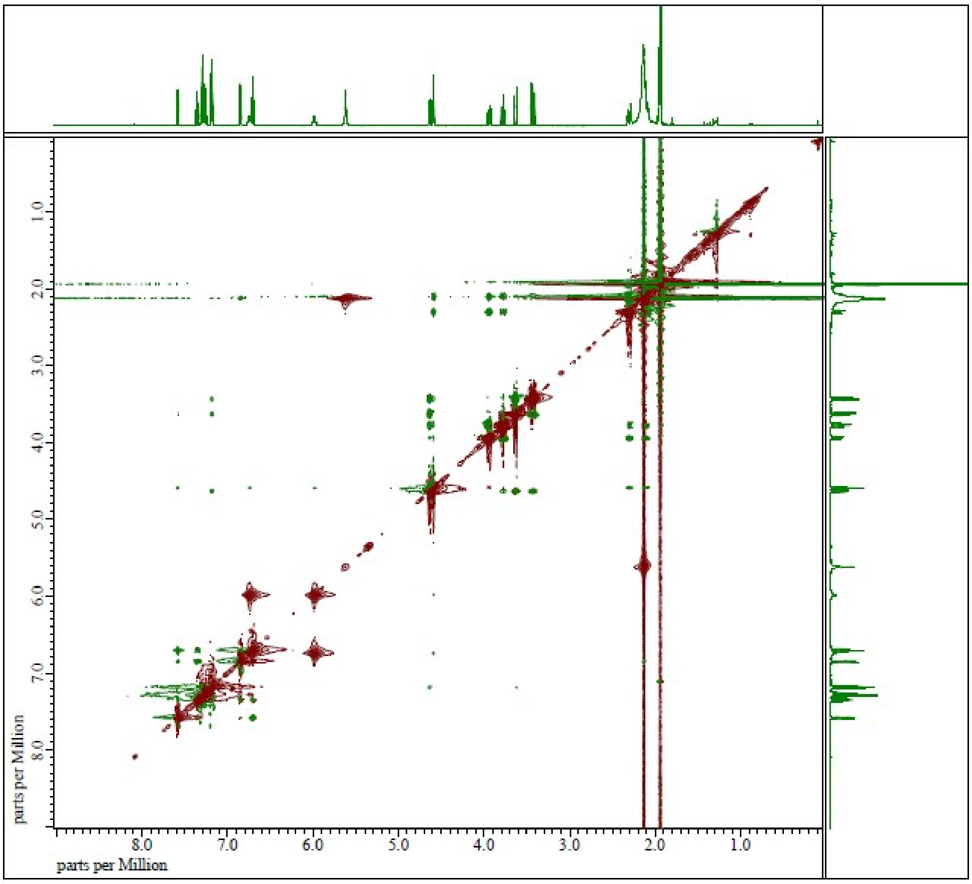

Figure S41. ^{1}H-^{1}H NOESY NMR spectrum of **7b^{2S}** (MeCN-d_3)

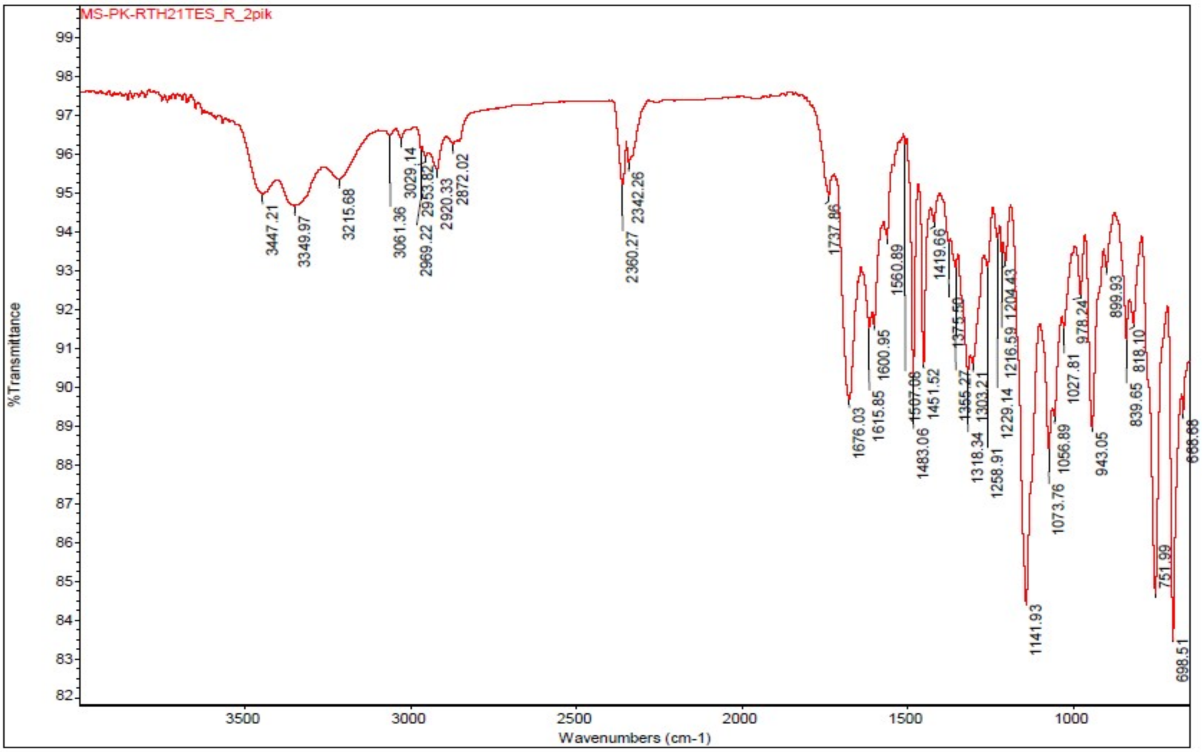

Figure S42. IR spectrum of **7b**^{2S}

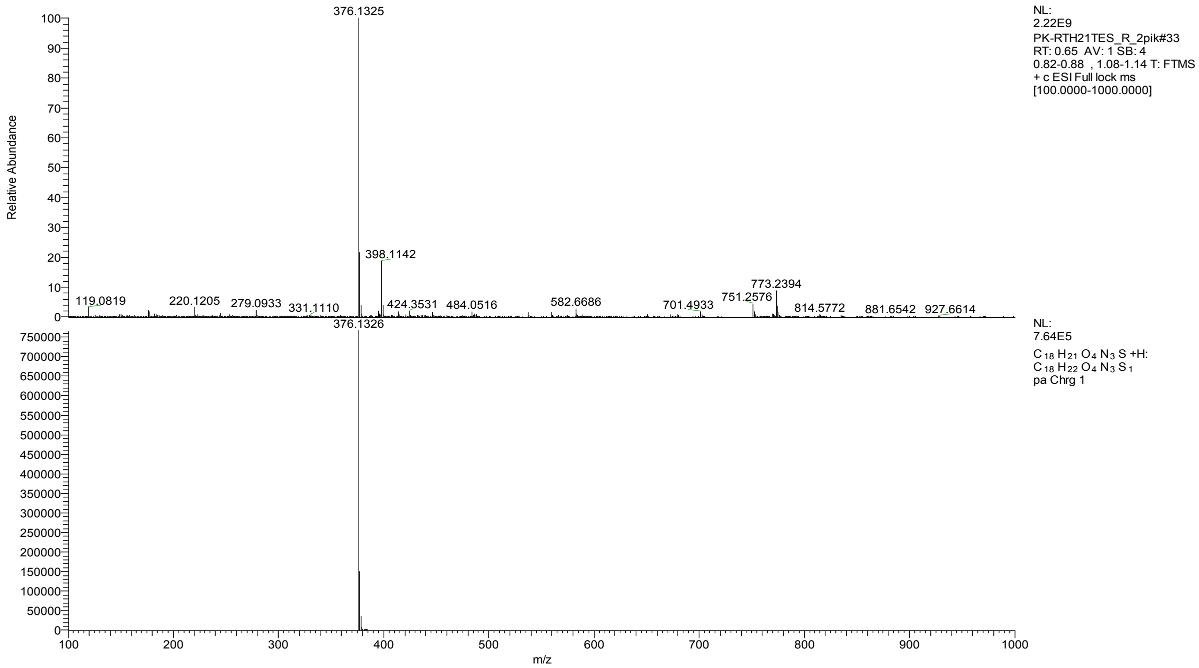

Figure S43. HRMS spectrum of **7b**^{2S}

(-)-(2*R*,5S)-4-((2-amino-4-methoxyphenyl)sulfonyl)-2-phenyl-1,4-oxazepane-5-carboxylic acid 7e

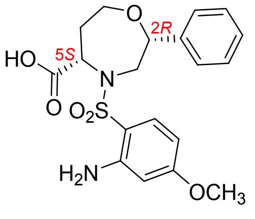

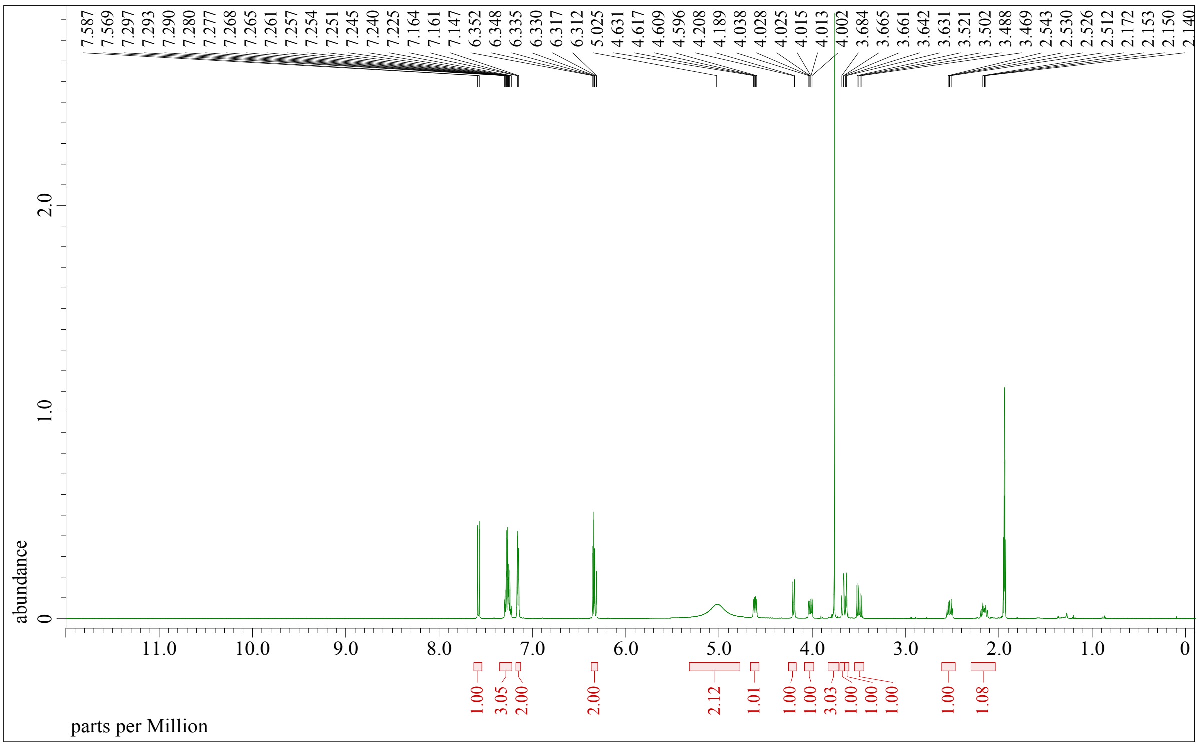

Figure S44. ^{1}H NMR spectrum of **7e** (500 MHz, MeCN-d_3)

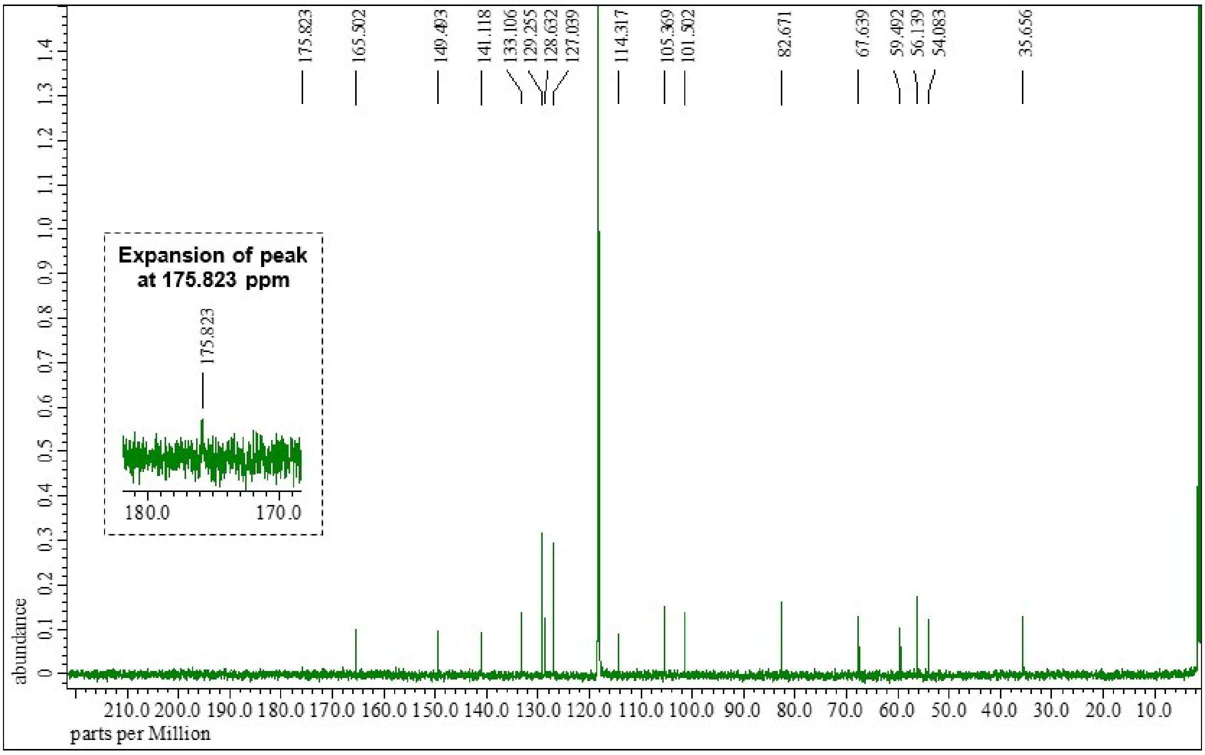

Figure S45. $^{13}C\{^{1}H\}$ NMR spectrum of **7e** (126 MHz, MeCN-d_3)

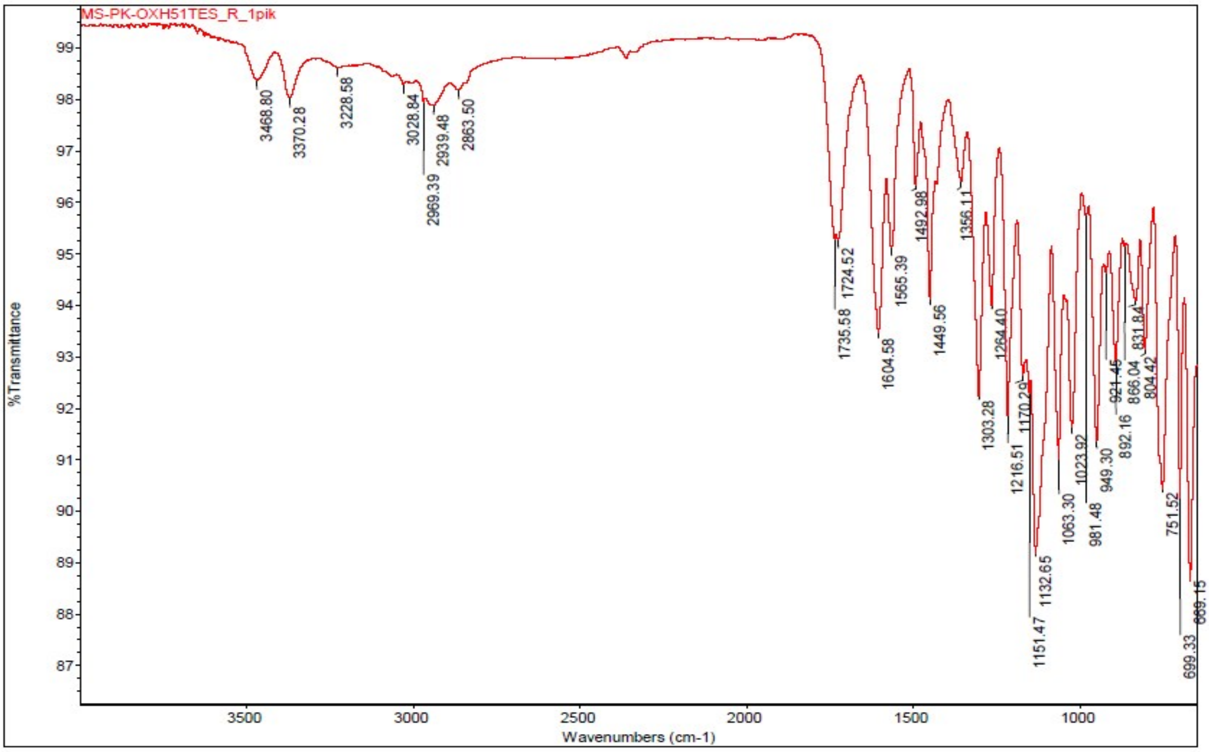

Figure S46. IR spectrum of **7e**

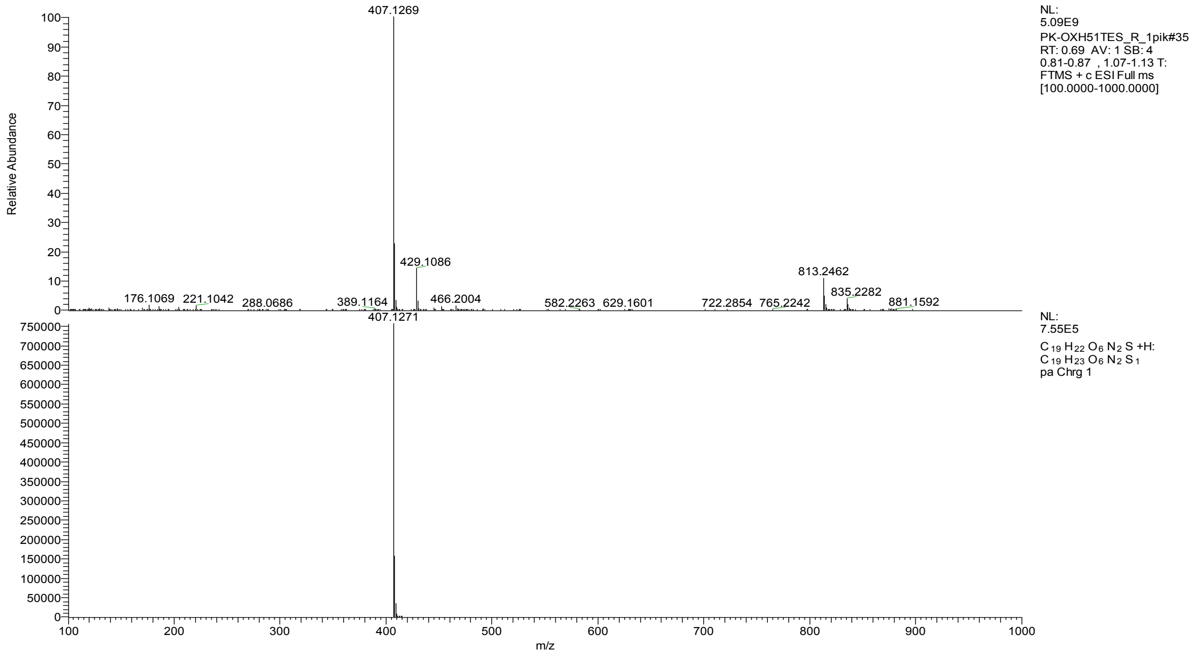

Figure S47. HRMS spectrum of **7e**

(-)-(2*RS*,5S)-4-((2-amino-4-chlorophenyl)sulfonyl)-2-phenyl-1,4-oxazepane-5-carboxylic acid 7f[2RS]

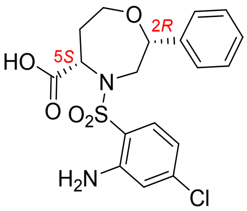

(A) 5*S*, 2*R* isomer

A:B = 78:22

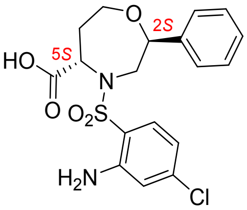

(B) 5*S*, 2*S* isomer

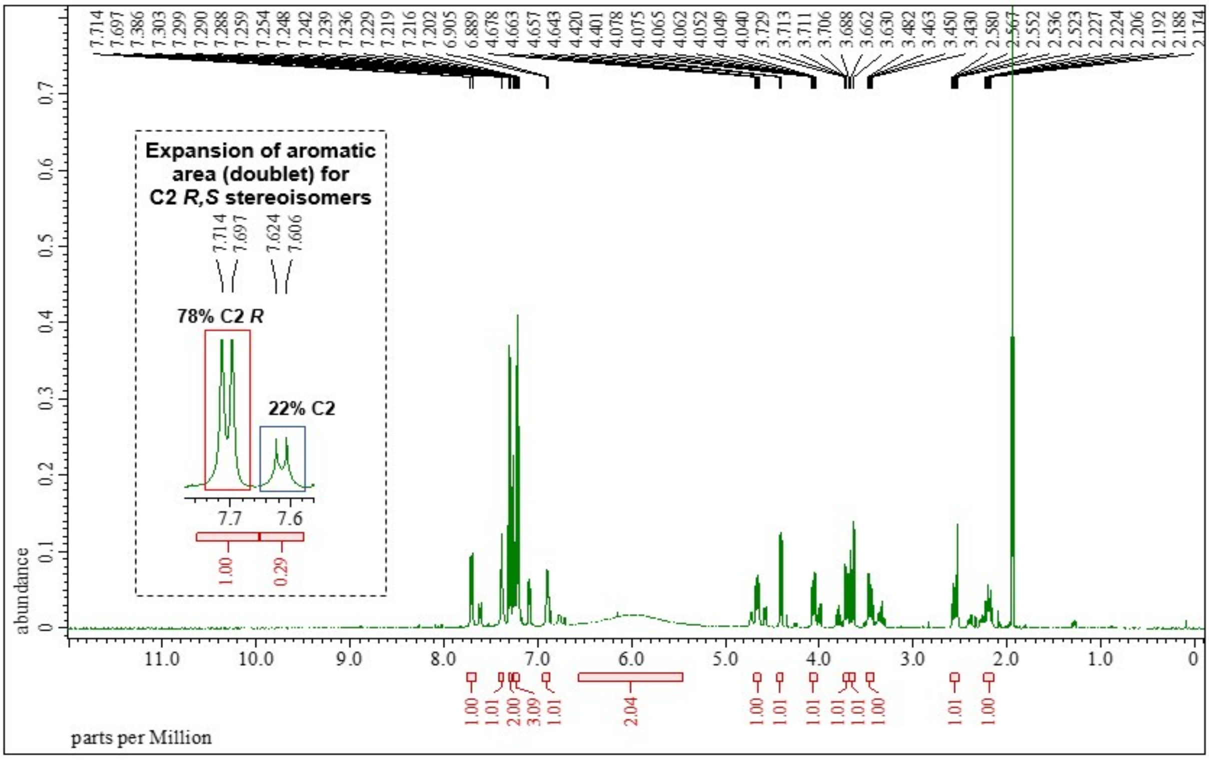

Figure S48. ^{1}H NMR spectrum of **7f^{2RS}** (500 MHz, MeCN-d_3). Note: Only the signals belonging to the major diastereomer were analyzed.

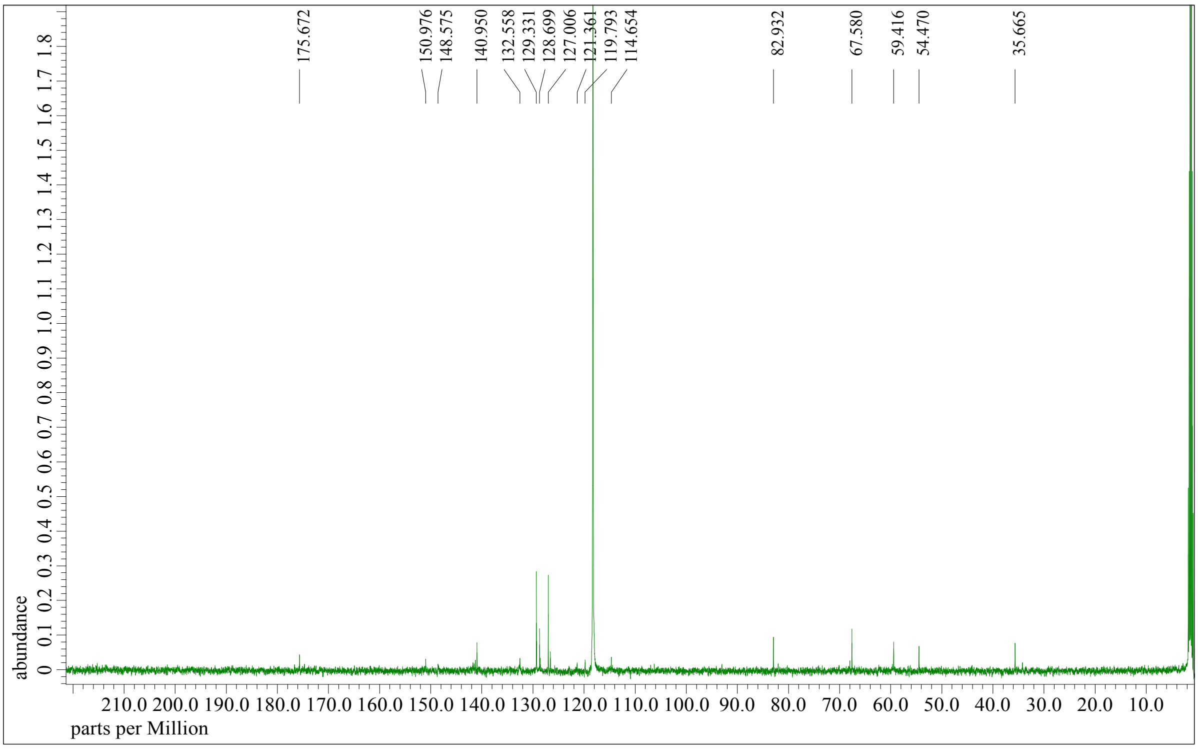

Figure S49. ^{13}C{^{1}H} NMR spectrum of **7f^{2RS}** (126 MHz, MeCN-d_3). Note: Only the signals belonging to the major diastereomer were analyzed.

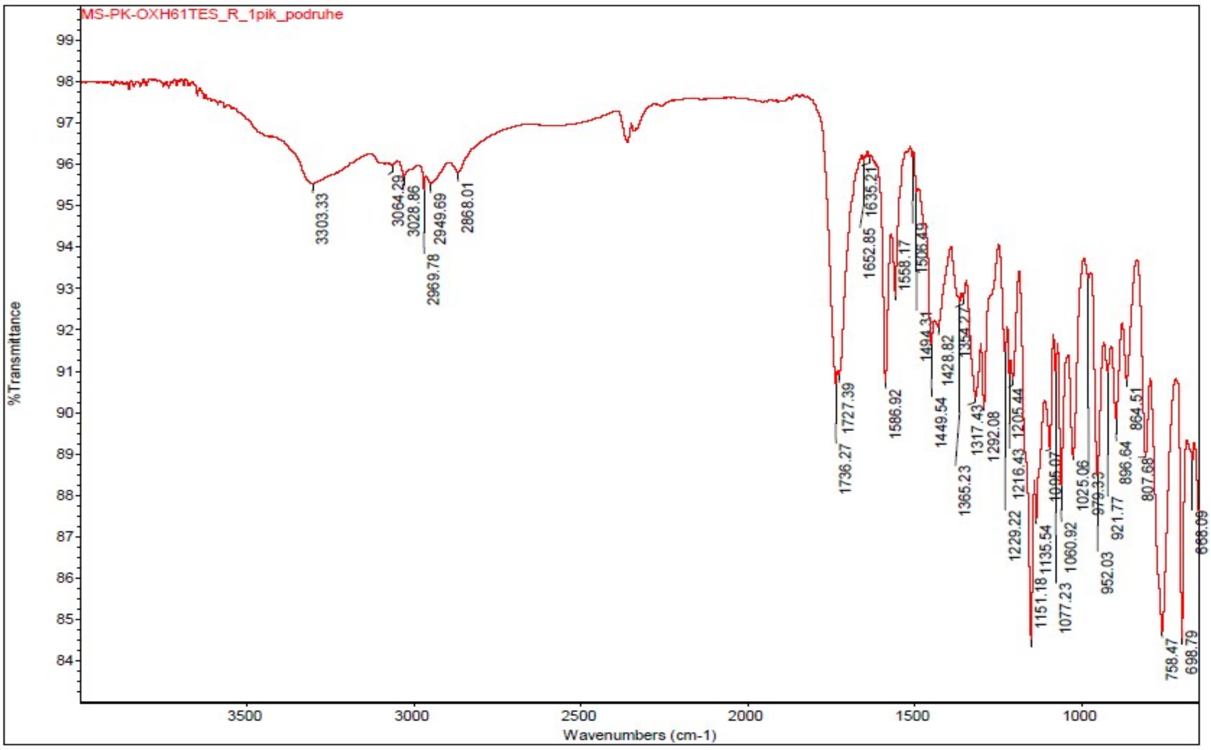

Figure S50. IR spectrum of **7f**^{2RS}

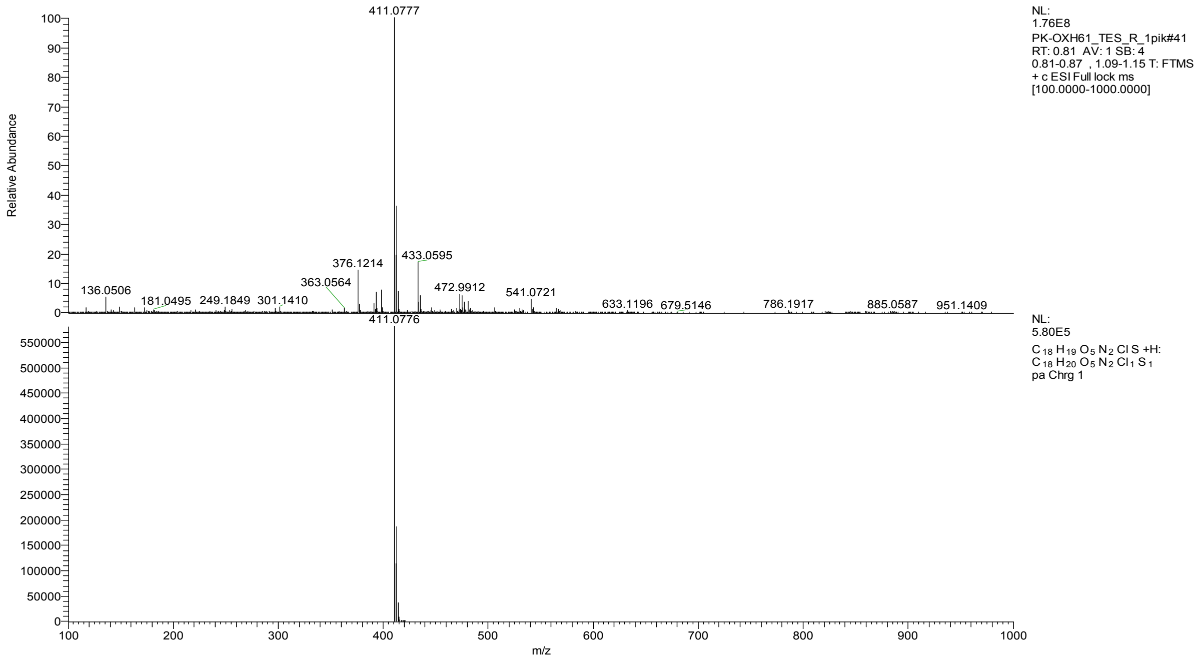

Figure S51. HRMS spectrum of **7f**^{2RS}

(-)-(2*R*,5S)-4-((2-aminophenyl)sulfonyl)-2-(2-fluorophenyl)-1,4-oxazepane-5-carboxylic acid 7i^{2R}

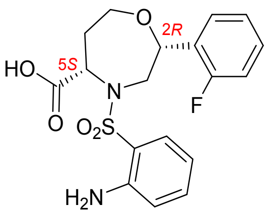

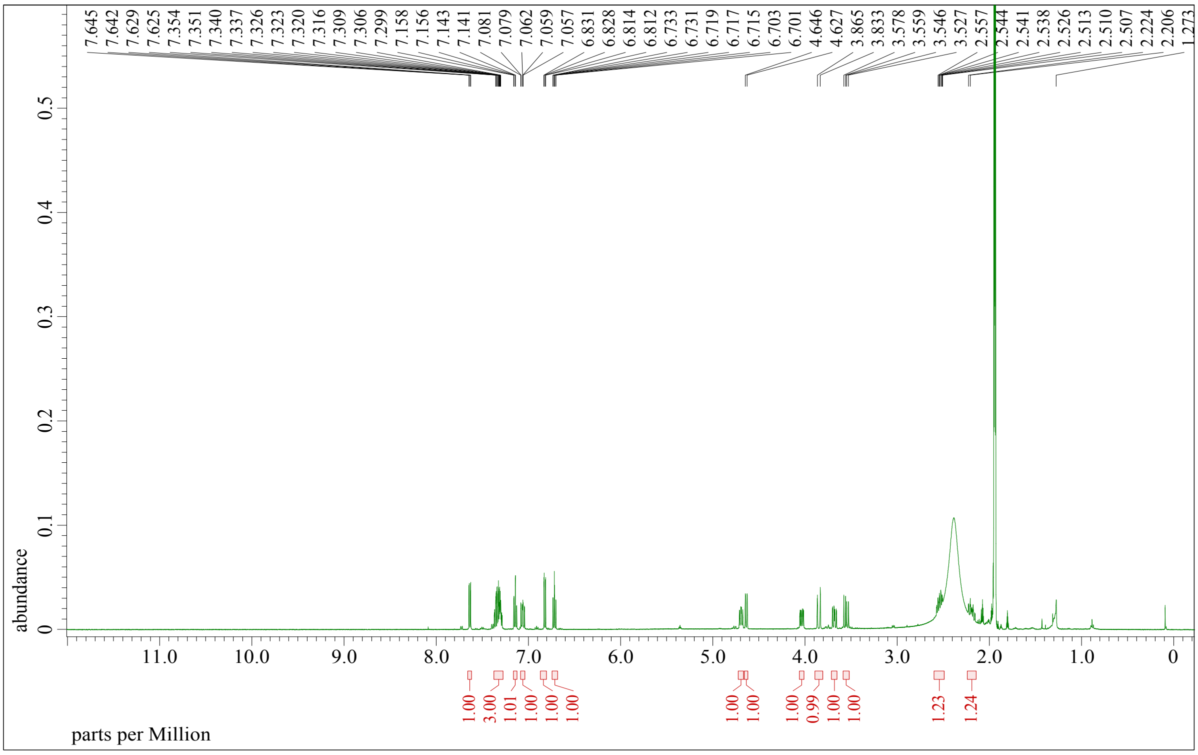

Figure S52. ^{1}H NMR spectrum of **7i**$^{\boldsymbol{2R}}$ (500 MHz, MeCN-d_3)

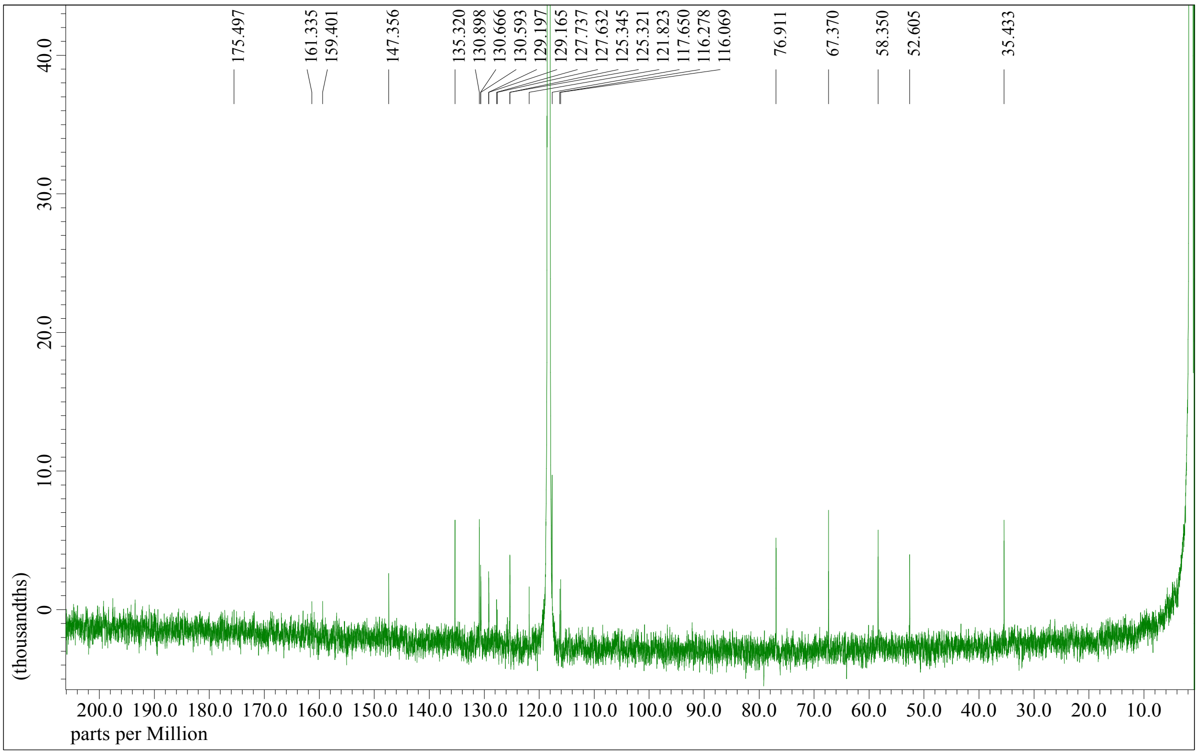

Figure S53. $^{13}C\{^{1}H\}$ NMR spectrum of **7i**$^{\boldsymbol{2R}}$ (126 MHz, MeCN-d_3)

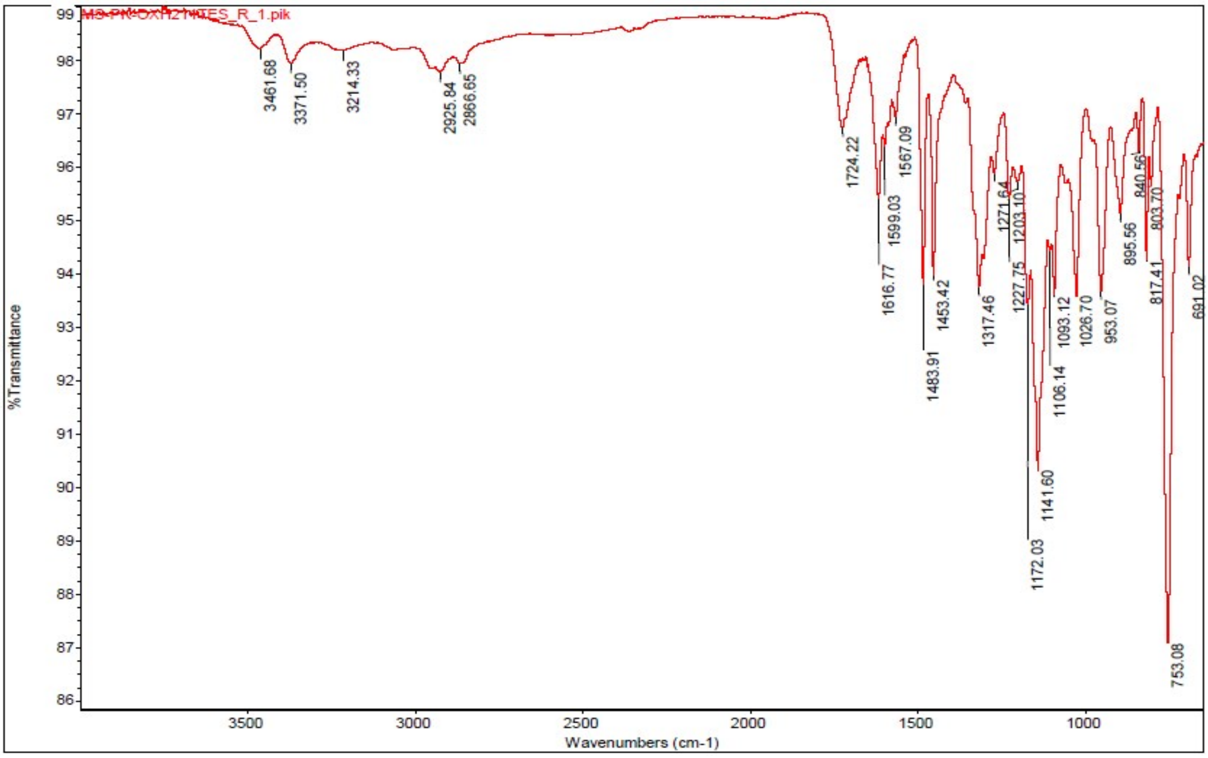

Figure S54. IR spectrum of **7i**^{2R}

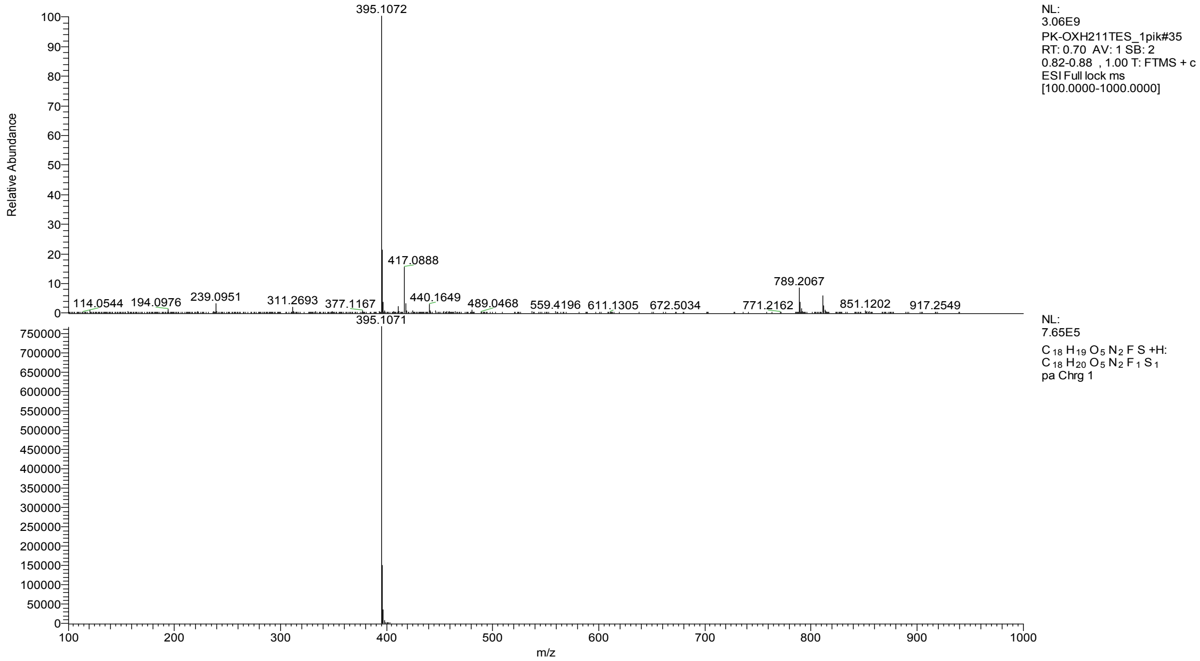

Figure S55. HRMS spectrum of **7i**^{2R}

(-)-(2*S*,5S)-4-((2-aminophenyl)sulfonyl)-2-(2-fluorophenyl)-1,4-oxazepane-5-carboxylic acid 7i^{2S}

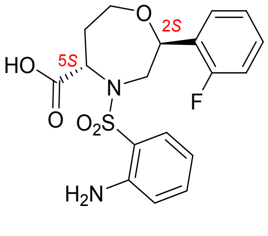

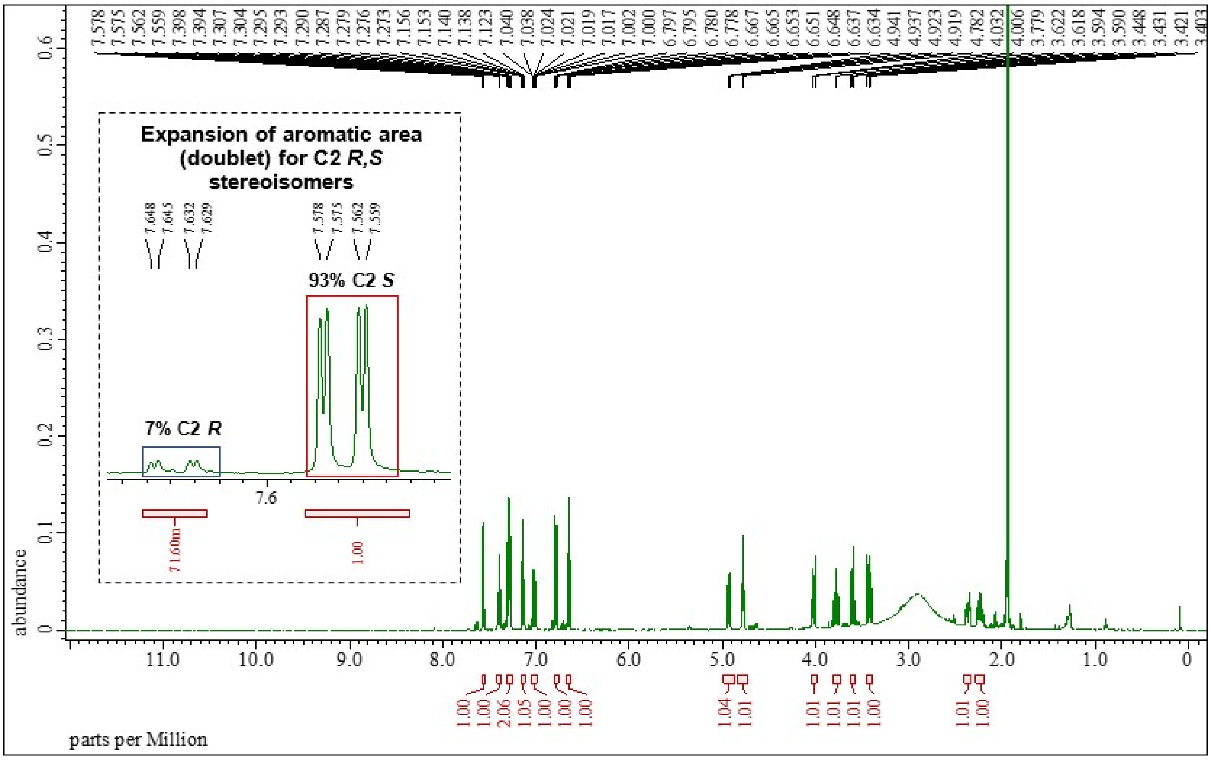

Figure S56. ^{1}H NMR spectrum of **7i^{2S}** (500 MHz, MeCN-d_3). Note: Only the signals belonging to the major diastereomer were analyzed.

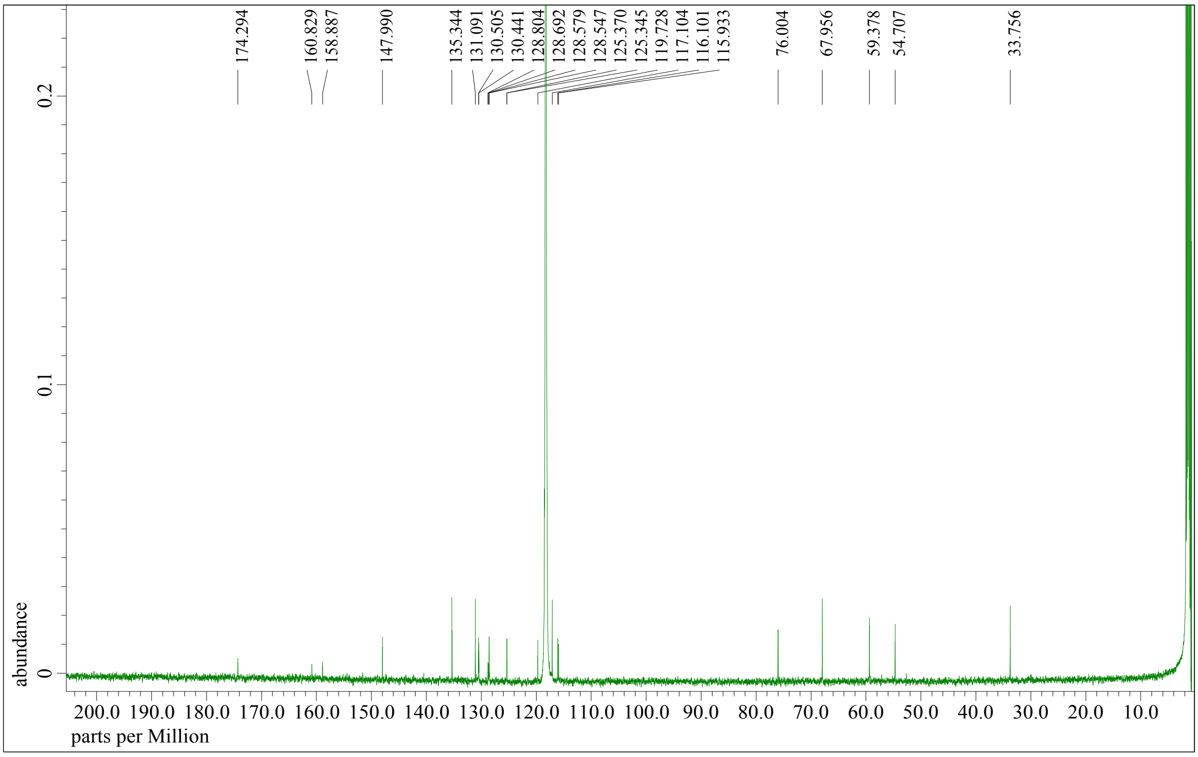

Figure S57. ^{13}C{^{1}H} NMR spectrum of **7i^{2S}** (126 MHz, MeCN-d_3). Note: Only the signals belonging to the major diastereomer were analyzed.

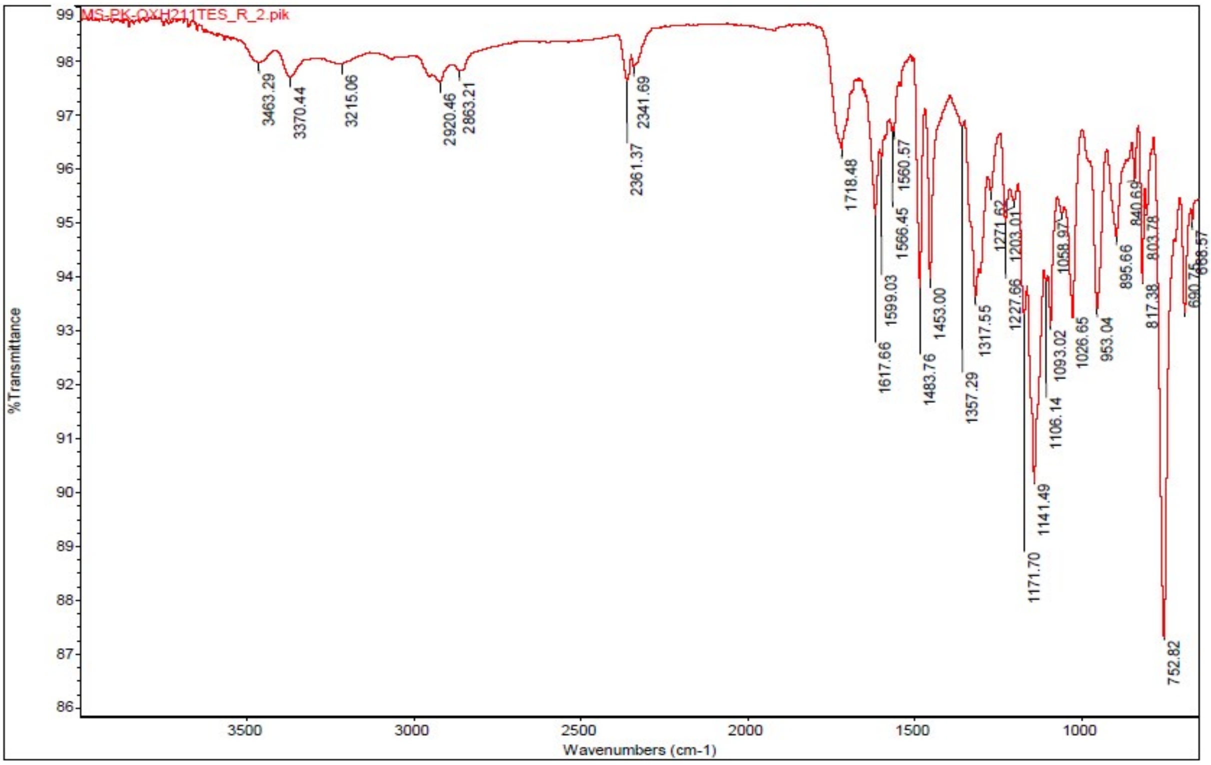

Figure S58. IR spectrum of **7i**[2S]

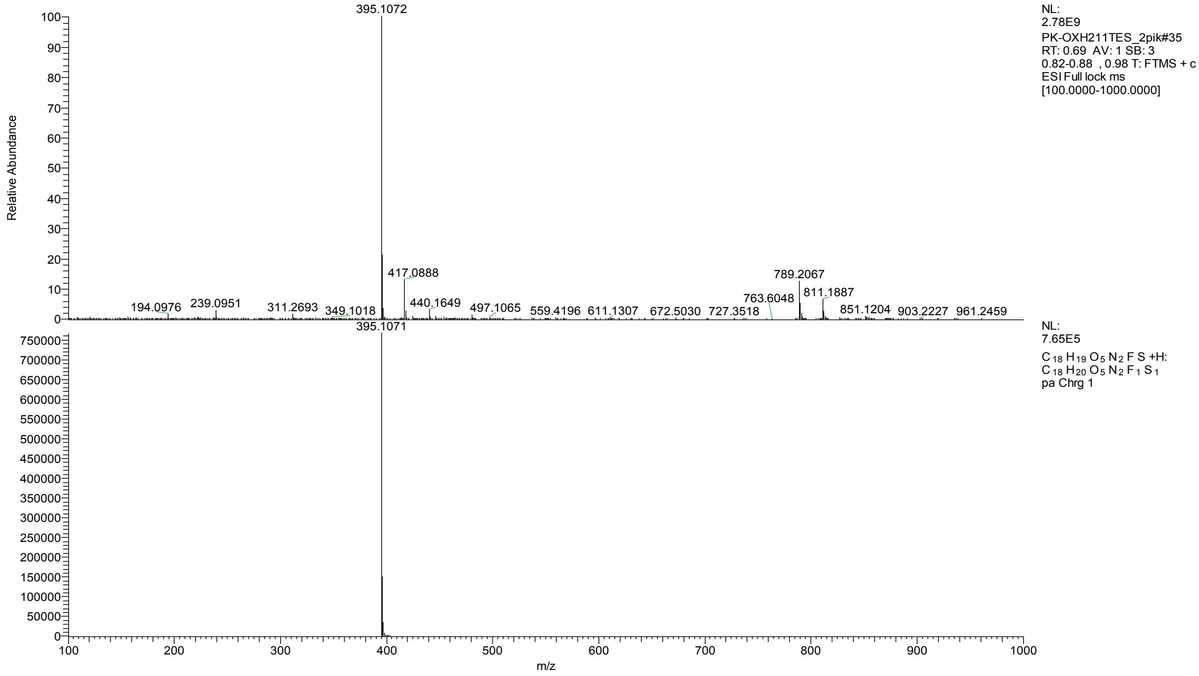

Figure S59. HRMS spectrum of **7i**[2S]

(-)-(2*R*,5S)-4-((2-aminophenyl)sulfonyl)-2-(3-fluorophenyl)-1,4-oxazepane-5-carboxylic acid 7k

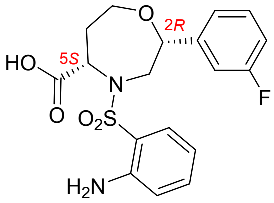

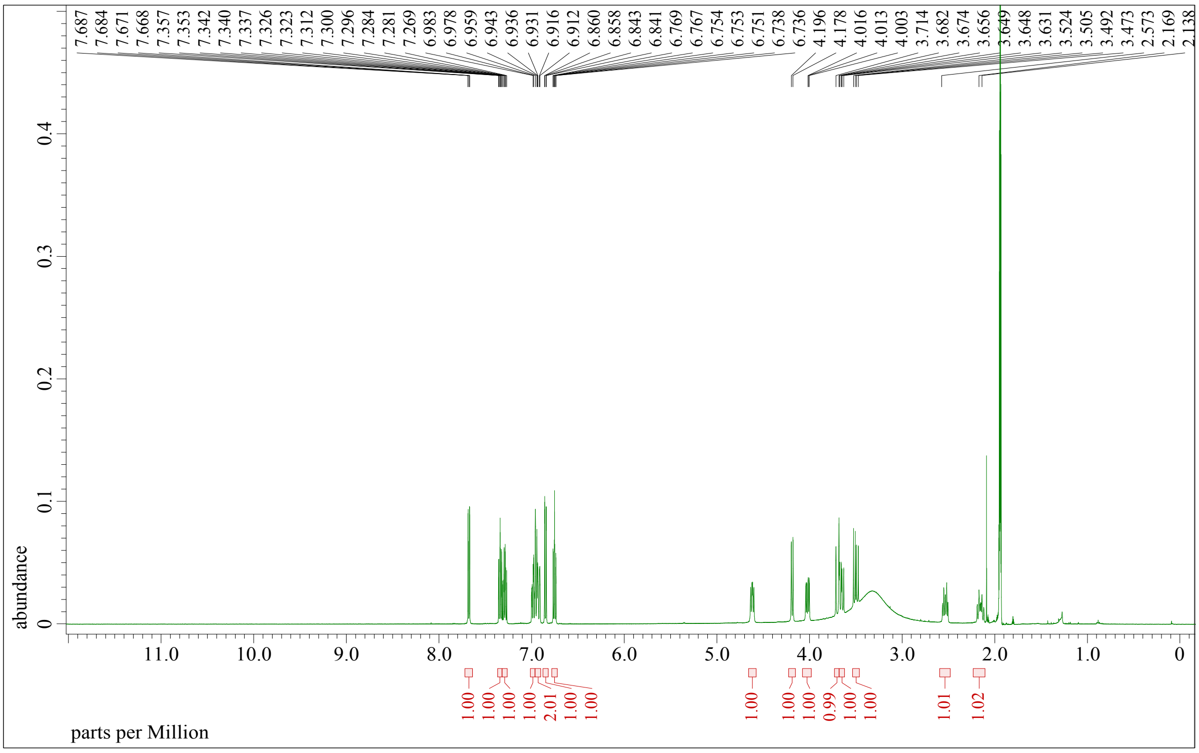

Figure S60. ^{1}H NMR spectrum of **7k** (500 MHz, MeCN-d_3)

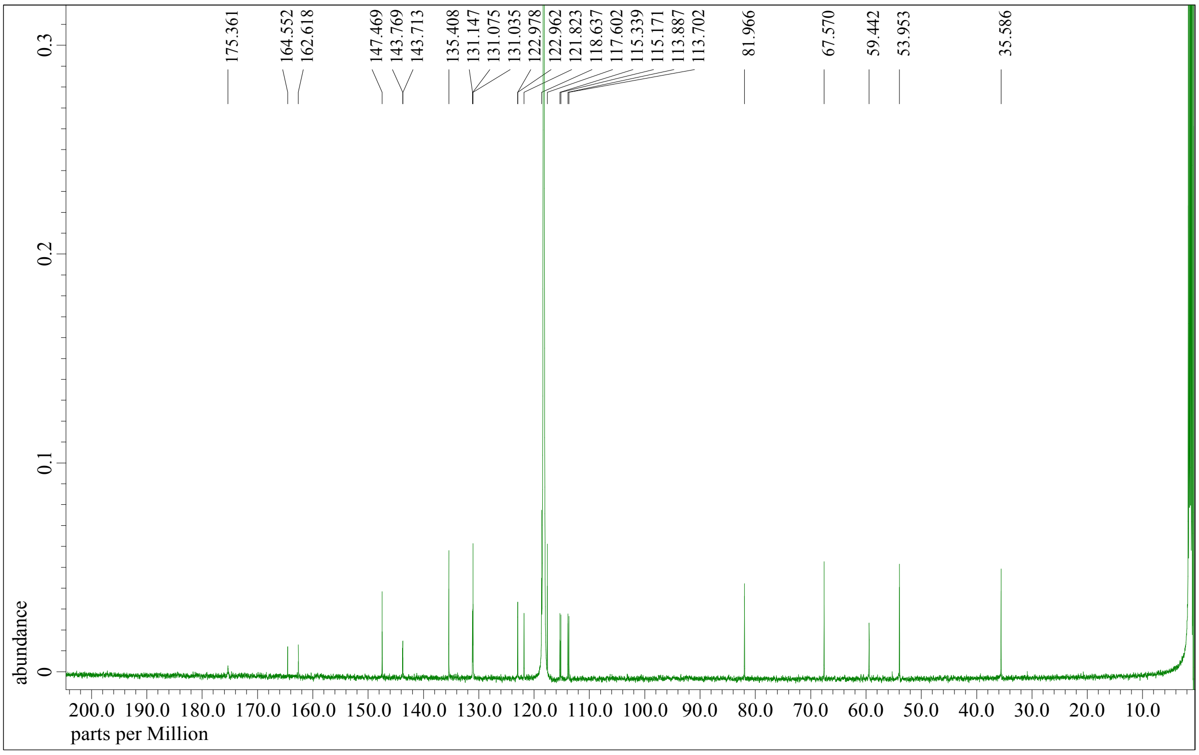

Figure S61. $^{13}C\{^{1}H\}$ NMR spectrum of **7k** (126 MHz, MeCN-d_3)

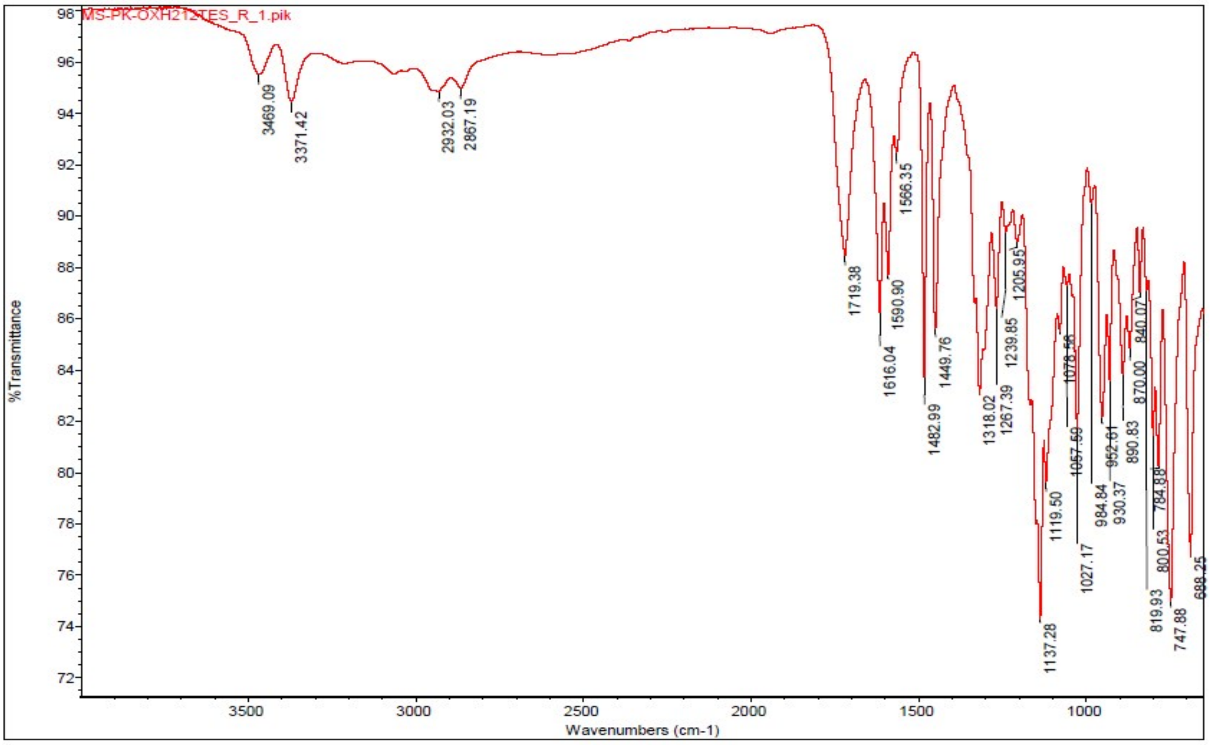

Figure S62. IR spectrum of **7k**

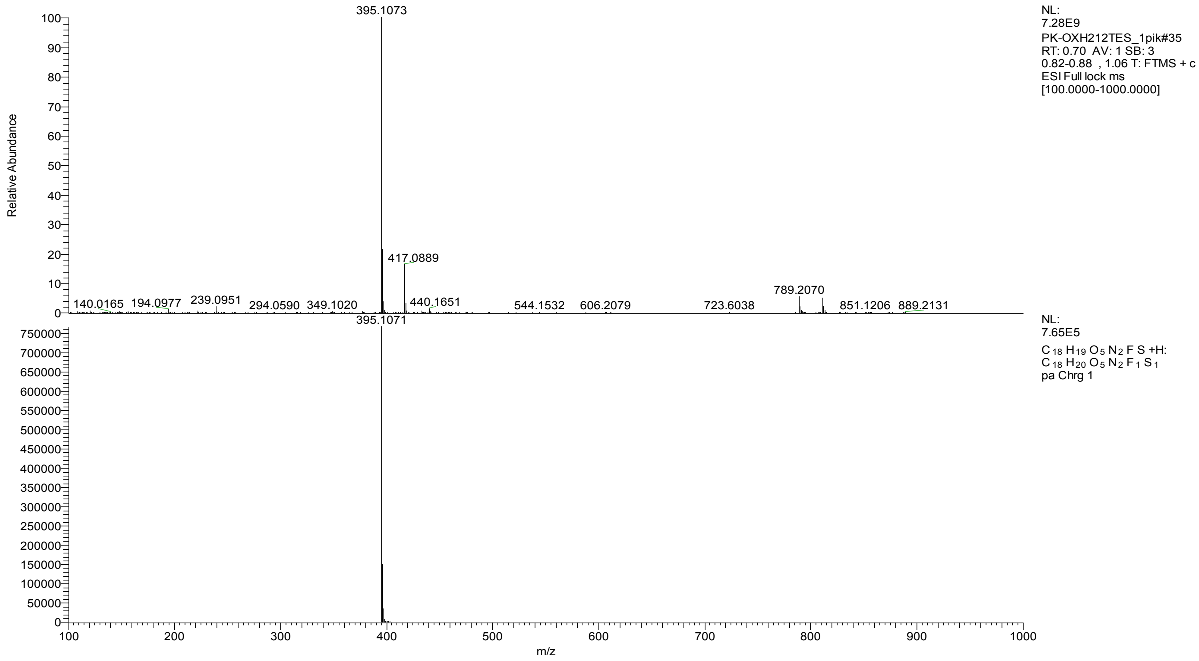

Figure S63. HRMS spectrum of **7k**

(+)-(2*R*,5S)-4-((2-aminophenyl)sulfonyl)-2-(*p*-tolyl)-1,4-oxazepane-5-carboxylic acid 7m

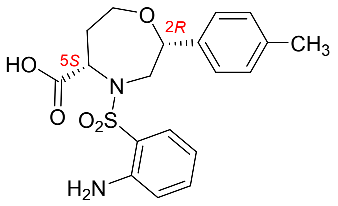

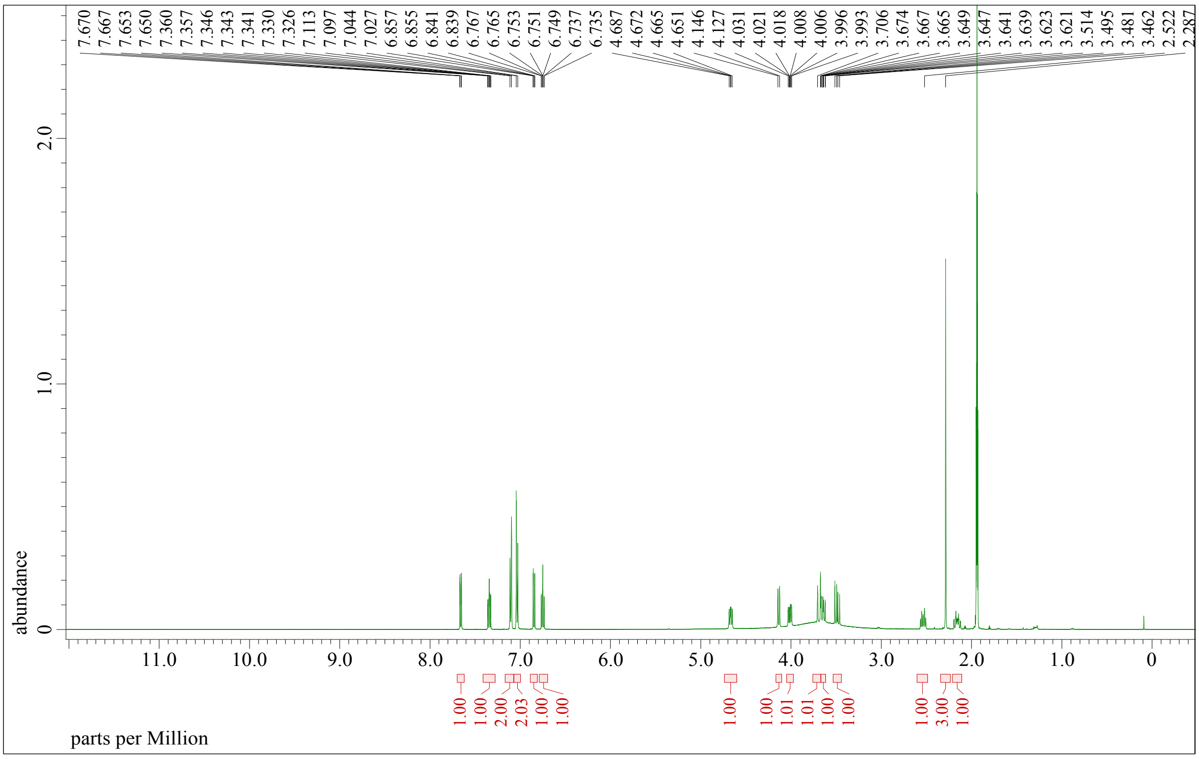

Figure S64. ^{1}H NMR spectrum of **7m** (500 MHz, MeCN-d_3)

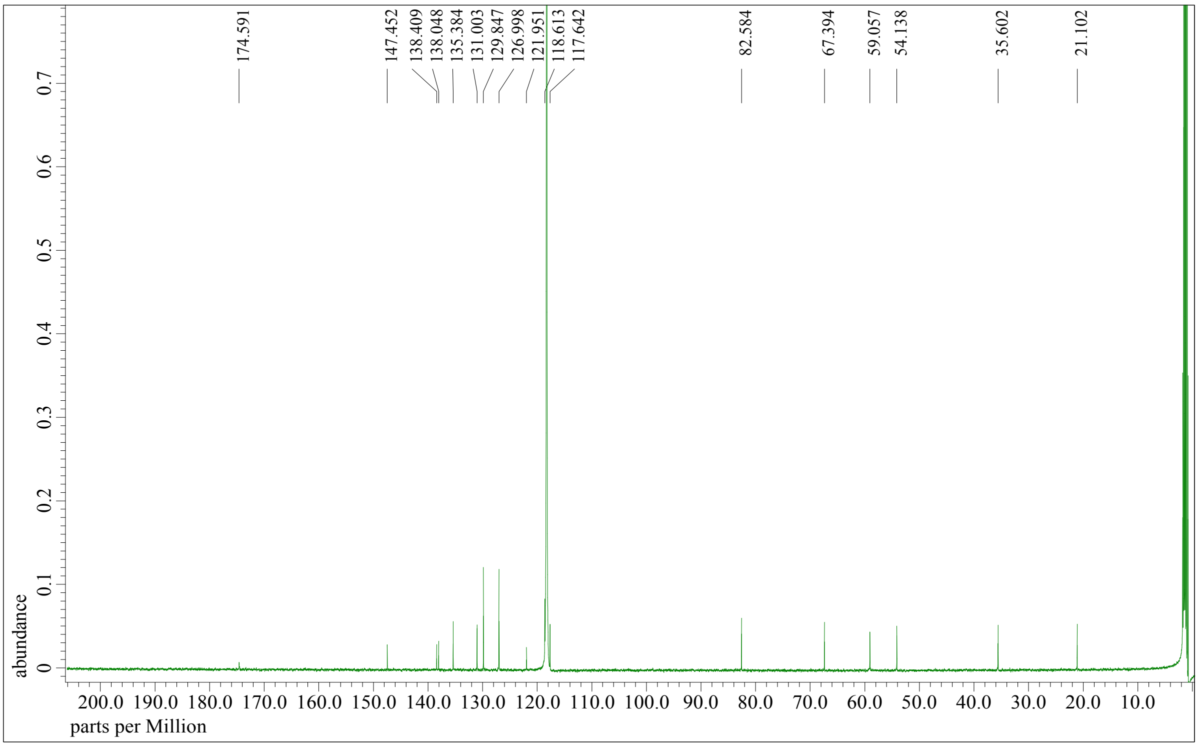

Figure S65. ^{13}C{^{1}H} NMR spectrum of **7m** (126 MHz, MeCN-d_3)

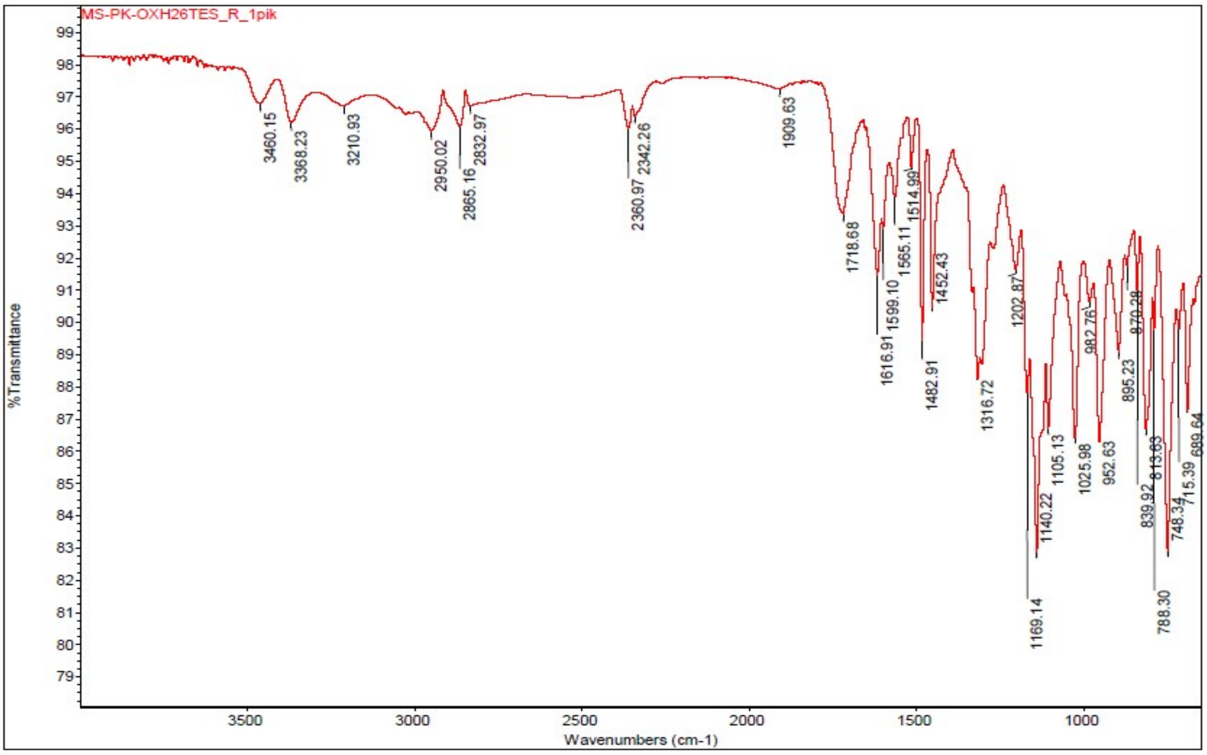

Figure S66. IR spectrum of **7m**

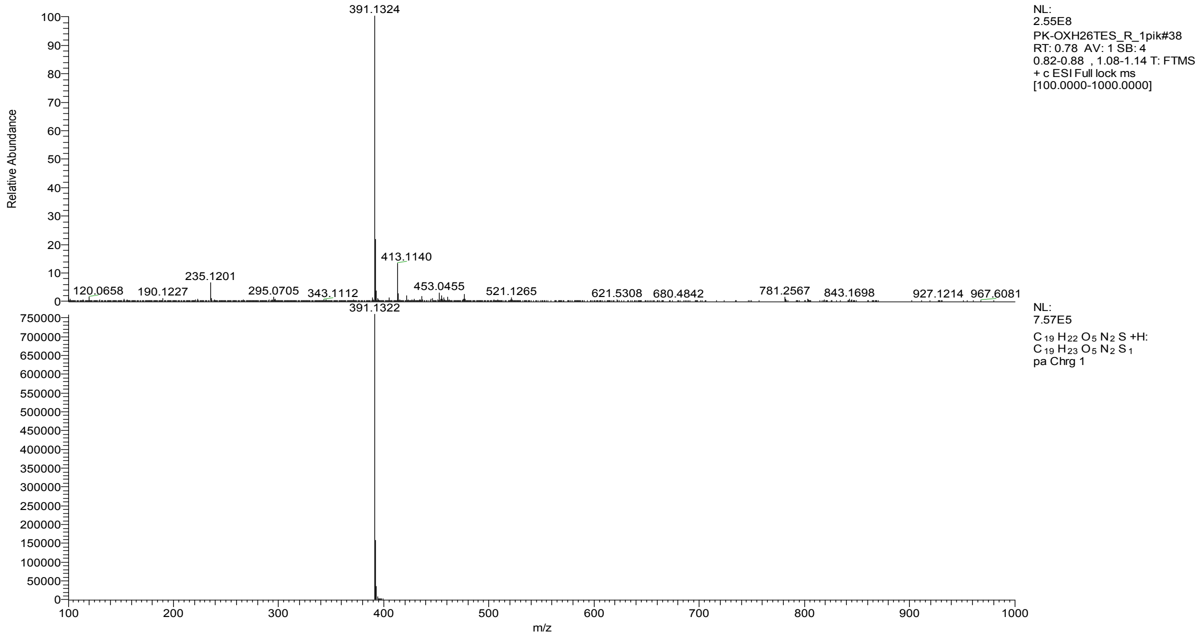

Figure S67. HRMS spectrum of **7m**

(+)-(2*R*,5S)-4-((2-aminophenyl)sulfonyl)-2-(4-fluorophenyl)-1,4-oxazepane-5-carboxylic acid 7o

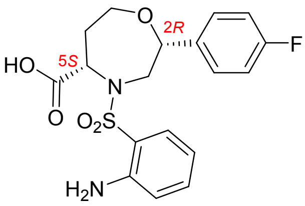

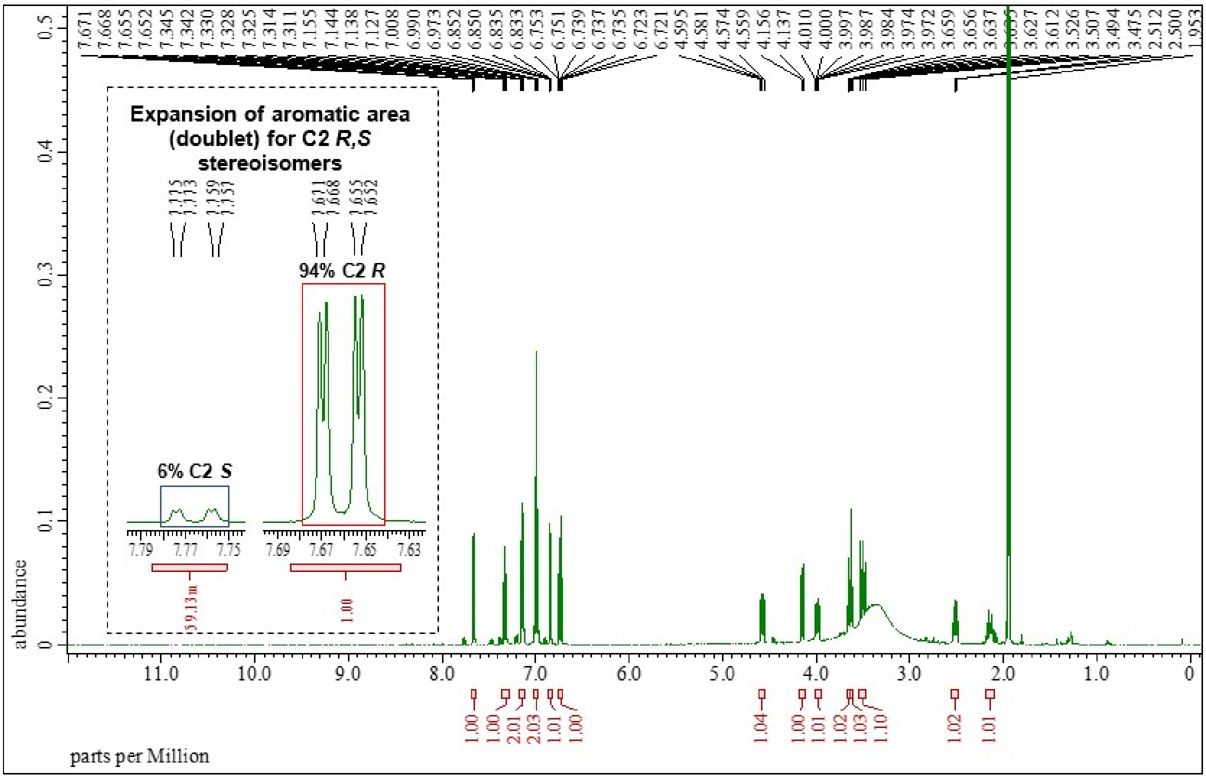

Figure S68. ^1H NMR spectrum of **7o** (500 MHz, MeCN-d_3). Note: Only the signals belonging to the major diastereomer were analyzed.

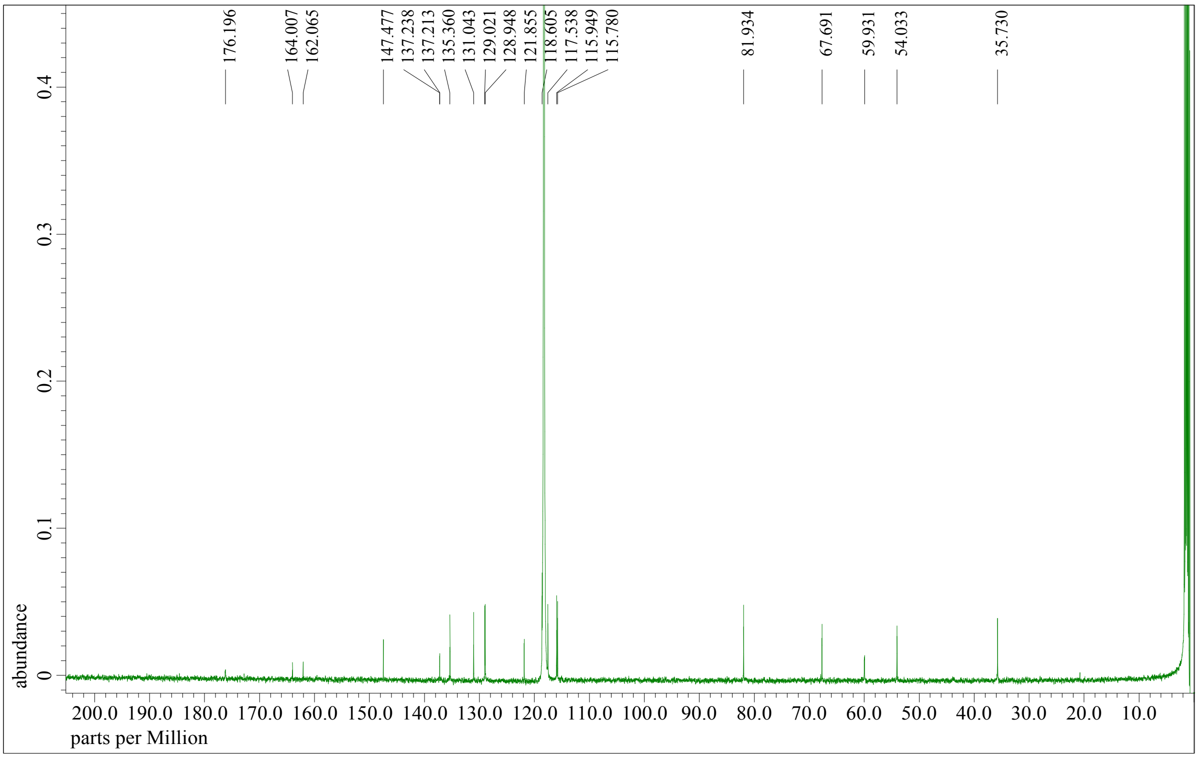

Figure S69. $^{13}C\{^1H\}$ NMR spectrum of **7o** (126 MHz, MeCN-d_3). Note: Only the signals belonging to the major diastereomer were analyzed.

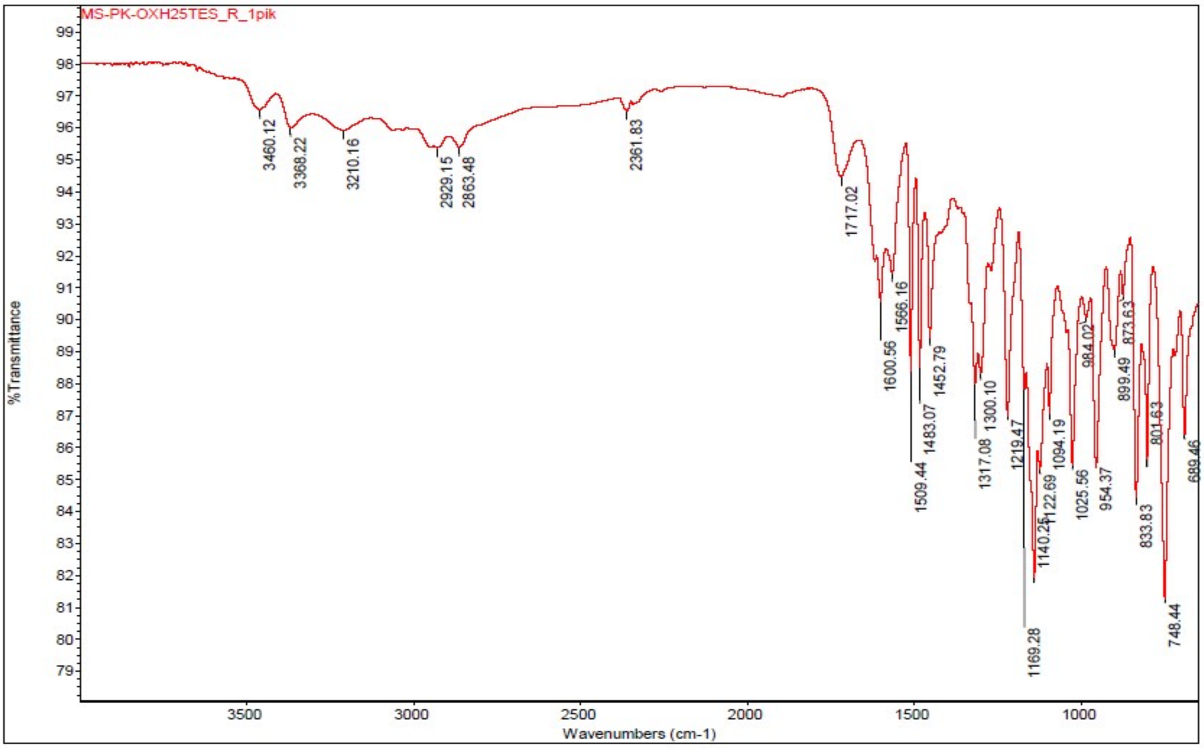

Figure S70. IR spectrum of **7o**

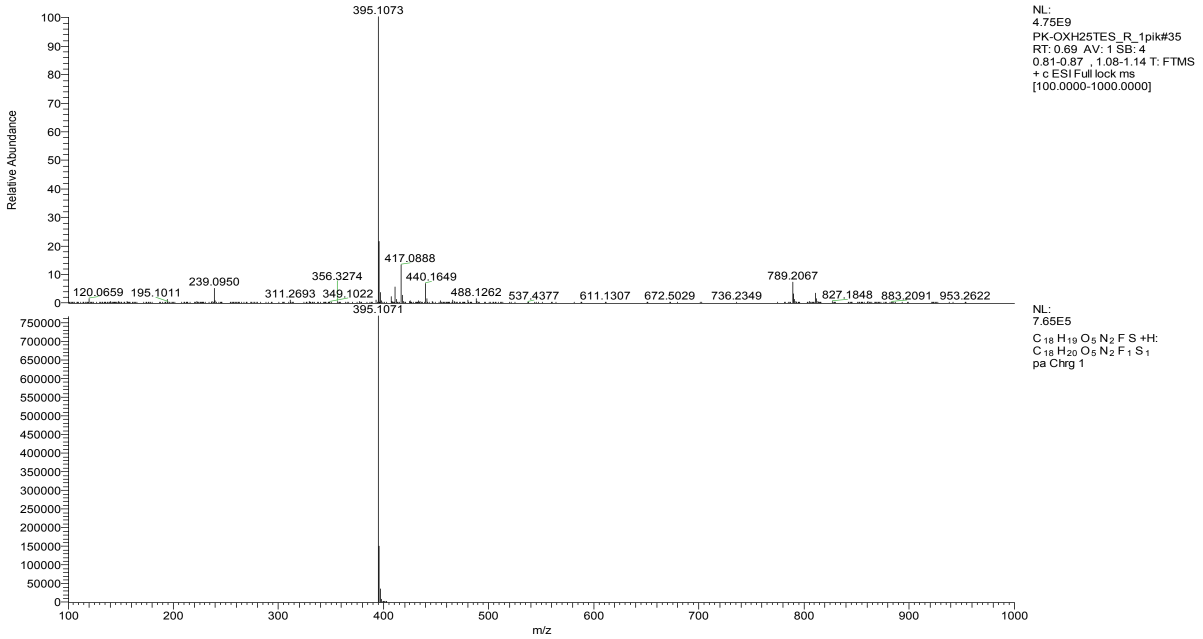

Figure S71. HRMS spectrum of **7o**

(-)-(2*R*,5S)-4-((2-aminophenyl)sulfonyl)-2-(4-(trifluoromethyl)phenyl)-1,4-oxazepane-5-carboxylic acid 7q

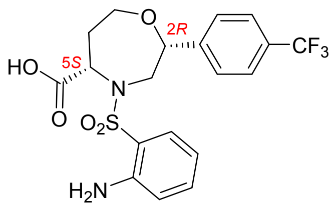

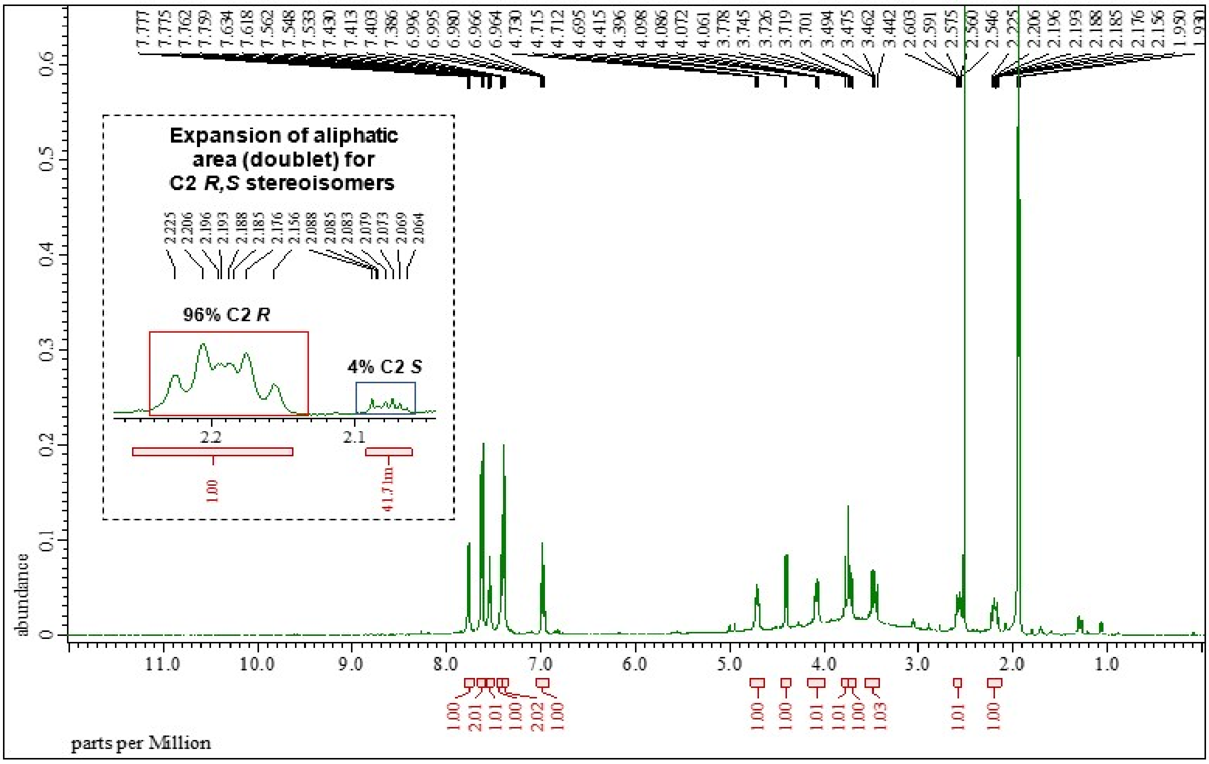

Figure S72. ^{1}H NMR spectrum of **7q** (500 MHz, MeCN-d_3). Note: Only the signals belonging to the major diastereomer were analyzed.

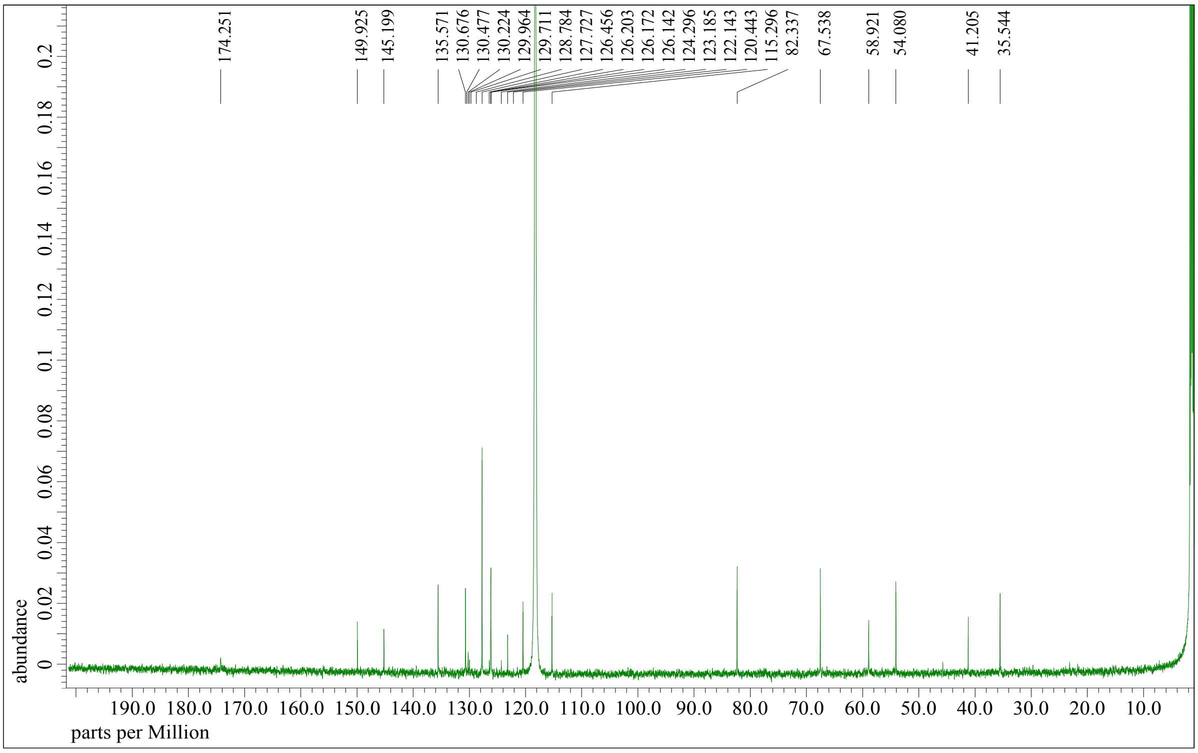

Figure S73. ^{13}C{^{1}H} NMR spectrum of **7q** (126 MHz, MeCN-d_3). Note: Only the signals belonging to the major diastereomer were analyzed.

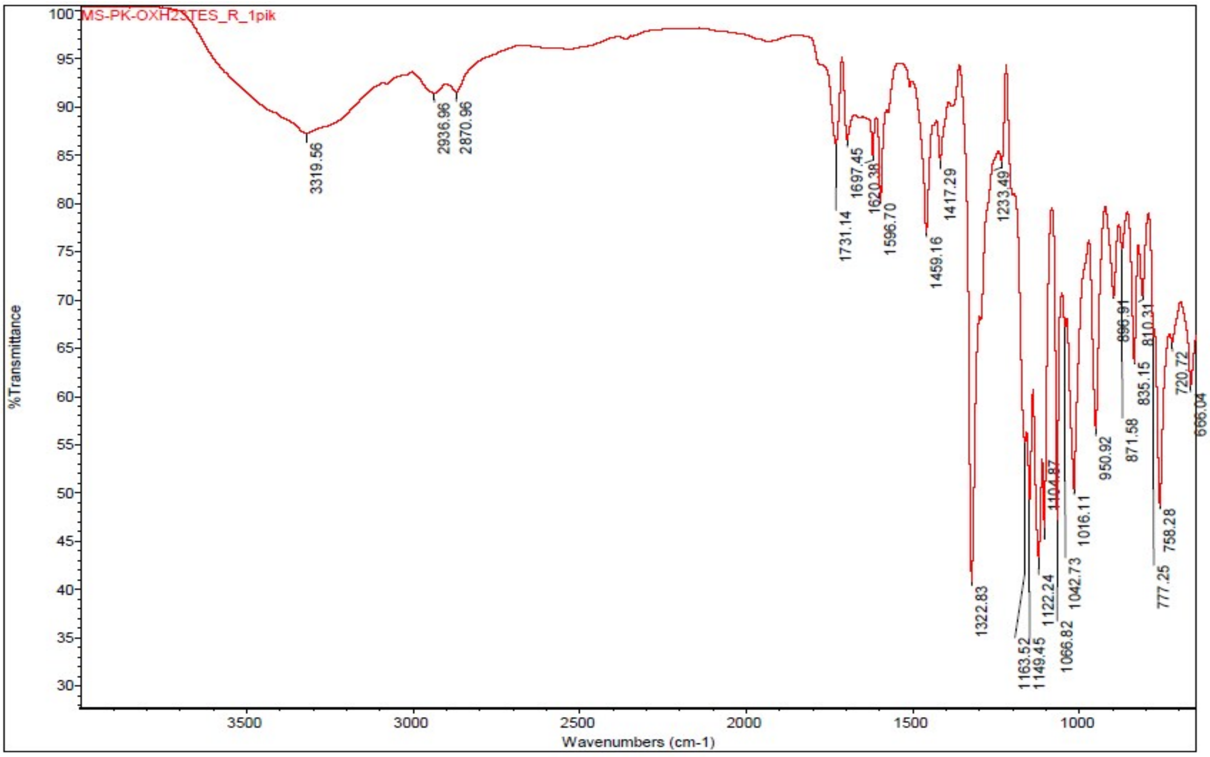

Figure S74. IR spectrum of **7q**

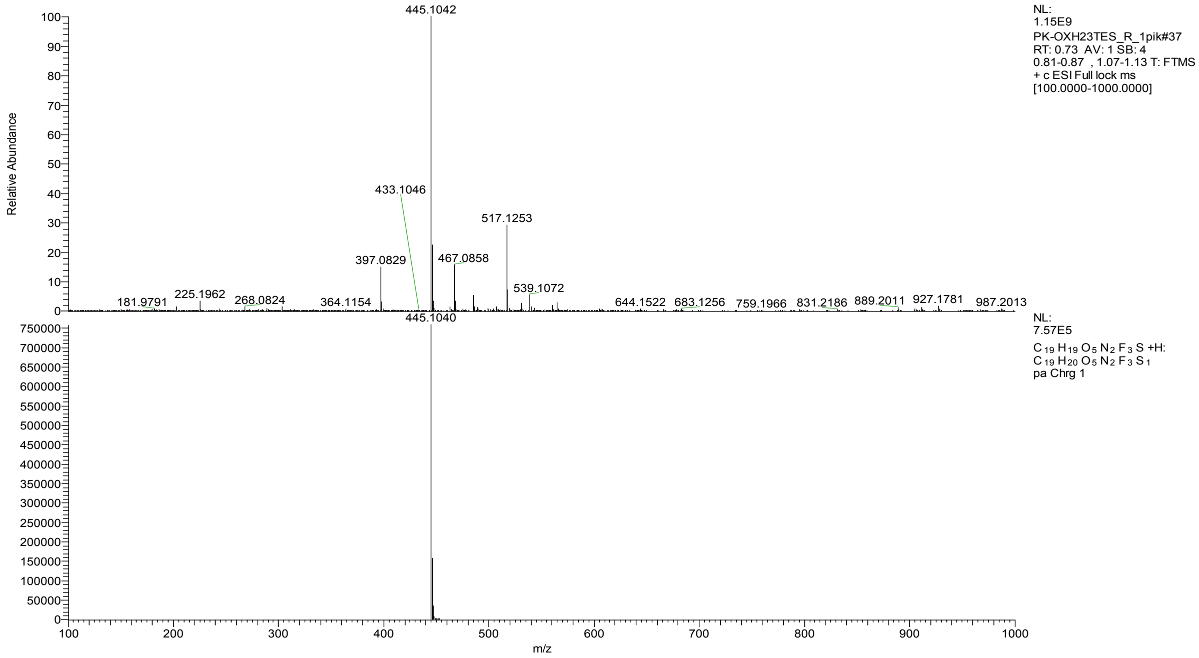

Figure S75. HRMS spectrum of **7q**

(-)-(2*R*,5S)-4-((2-aminophenyl)sulfonyl)-2-(thiophen-3-yl)-1,4-oxazepane-5-carboxylic acid 7t

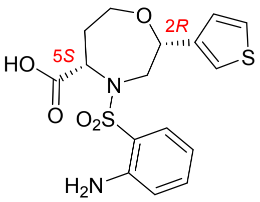

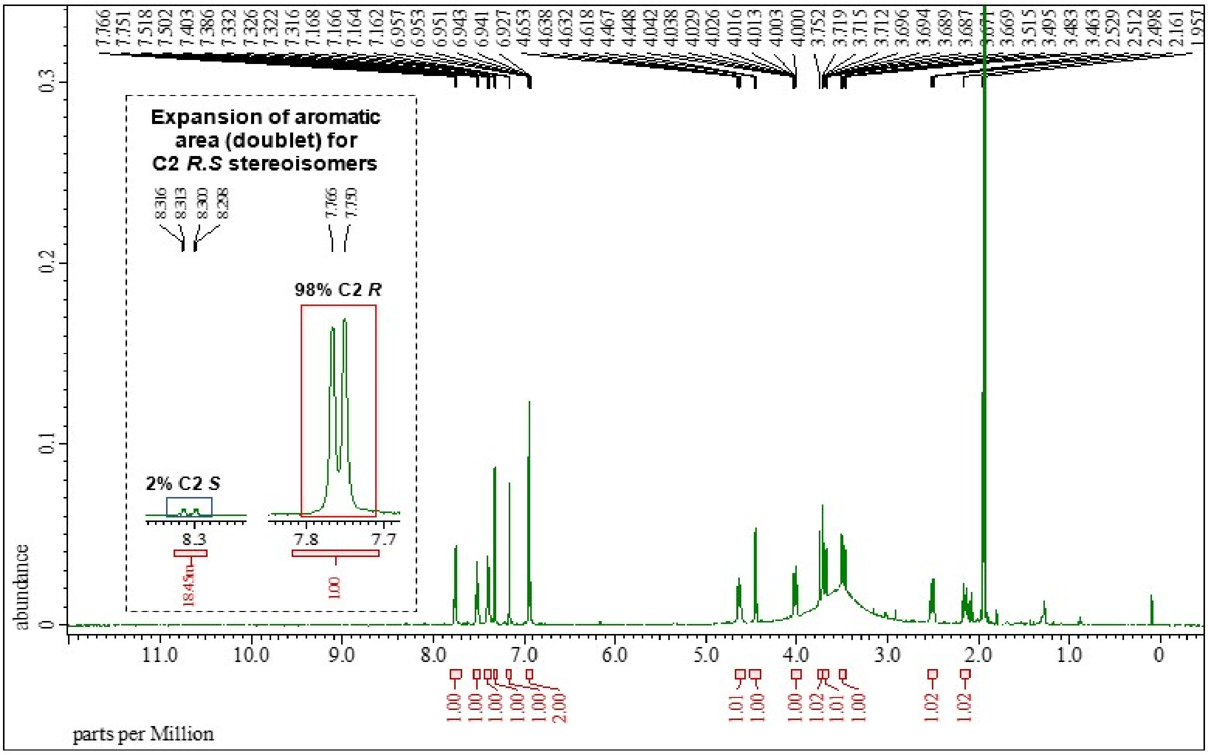

Figure S76. ^{1}H NMR spectrum of **7t** (500 MHz, MeCN-d_3)

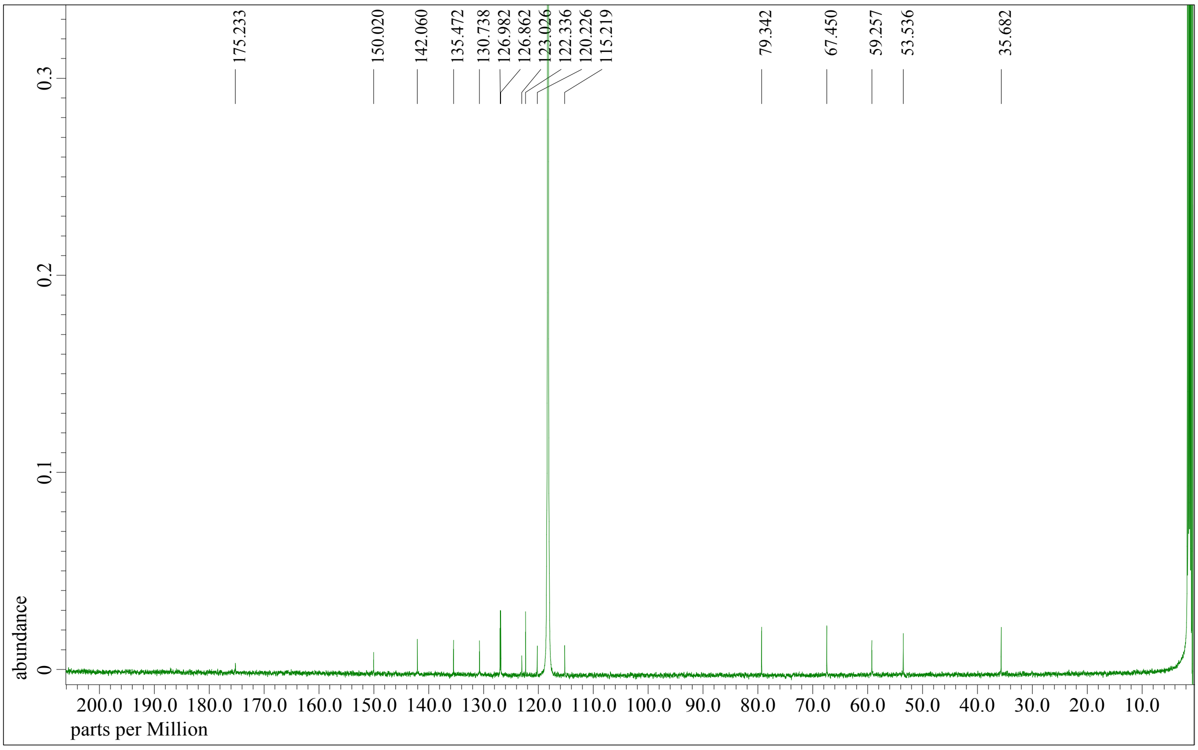

Figure S77. $^{13}C\{^{1}H\}$ NMR spectrum of **7t** (126 MHz, MeCN-d_3)

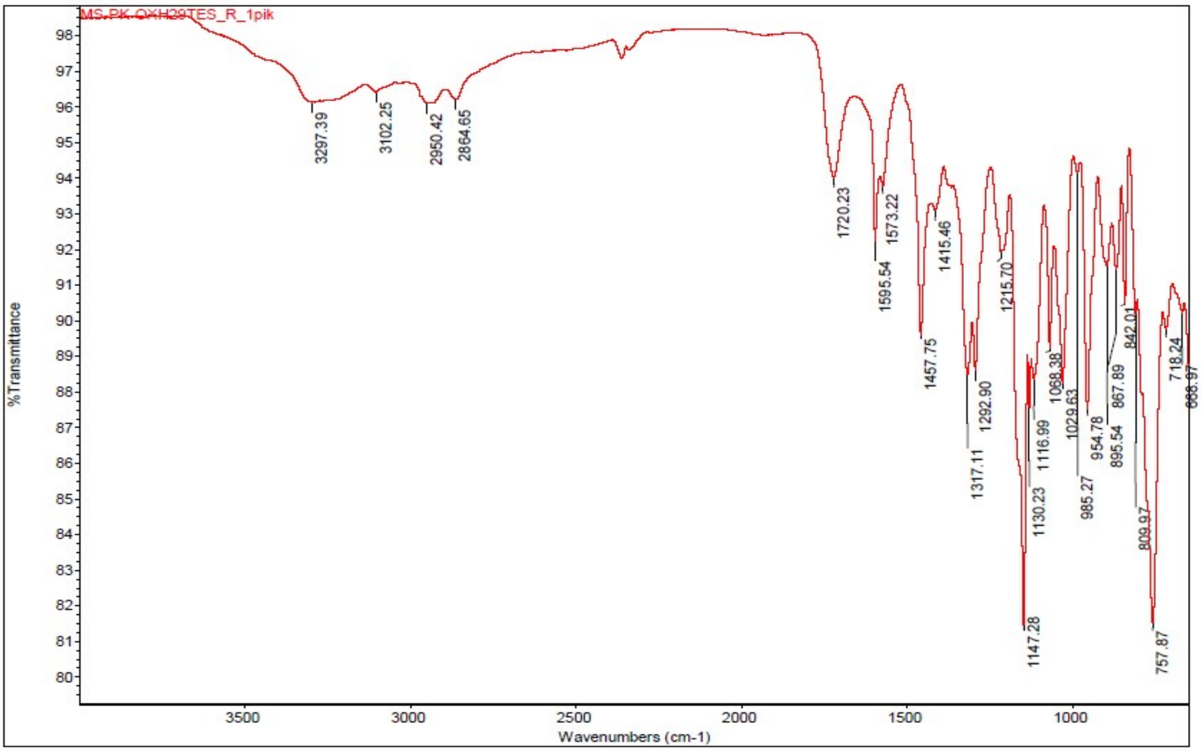

Figure S78. IR spectrum of **7t**

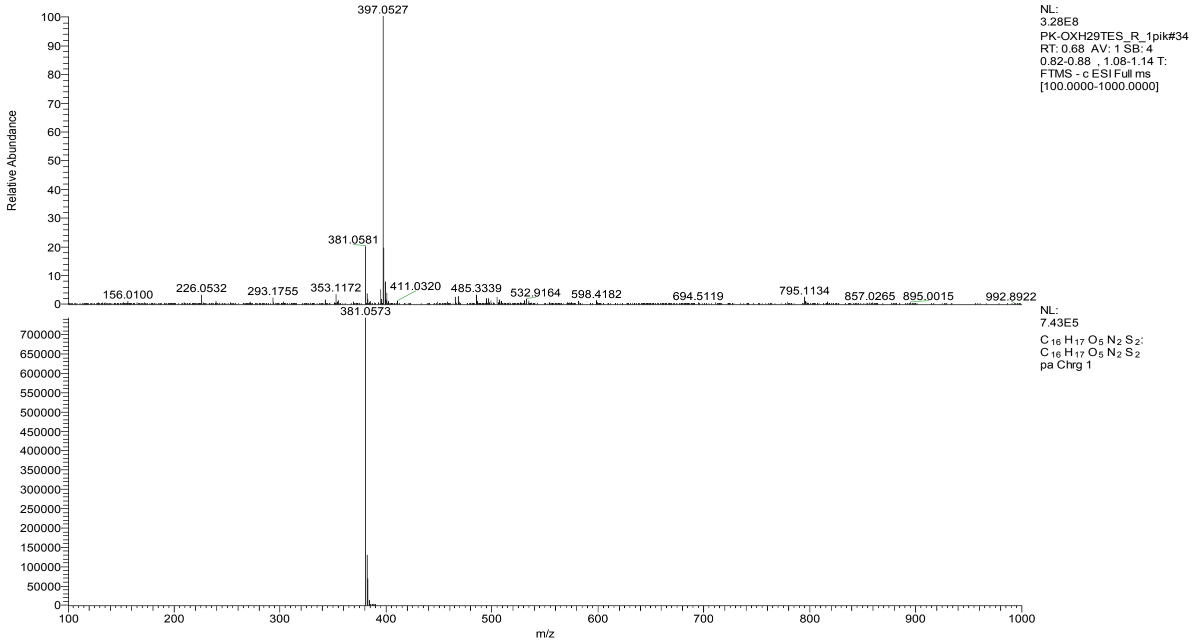

Figure S79. HRMS spectrum of **7t**. Note: ion 397 belongs to the ammonium adduct originating from mobile phase.

(-)-(*S*)-3-(1,1-dioxo-4-(*o*-tolyl)benzo[*f*][1,2,5]thiadiazepin-2(5*H*)-yl)dihydrofuran-2(3*H*)-one 9h

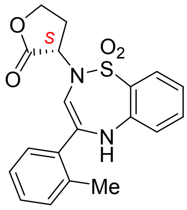

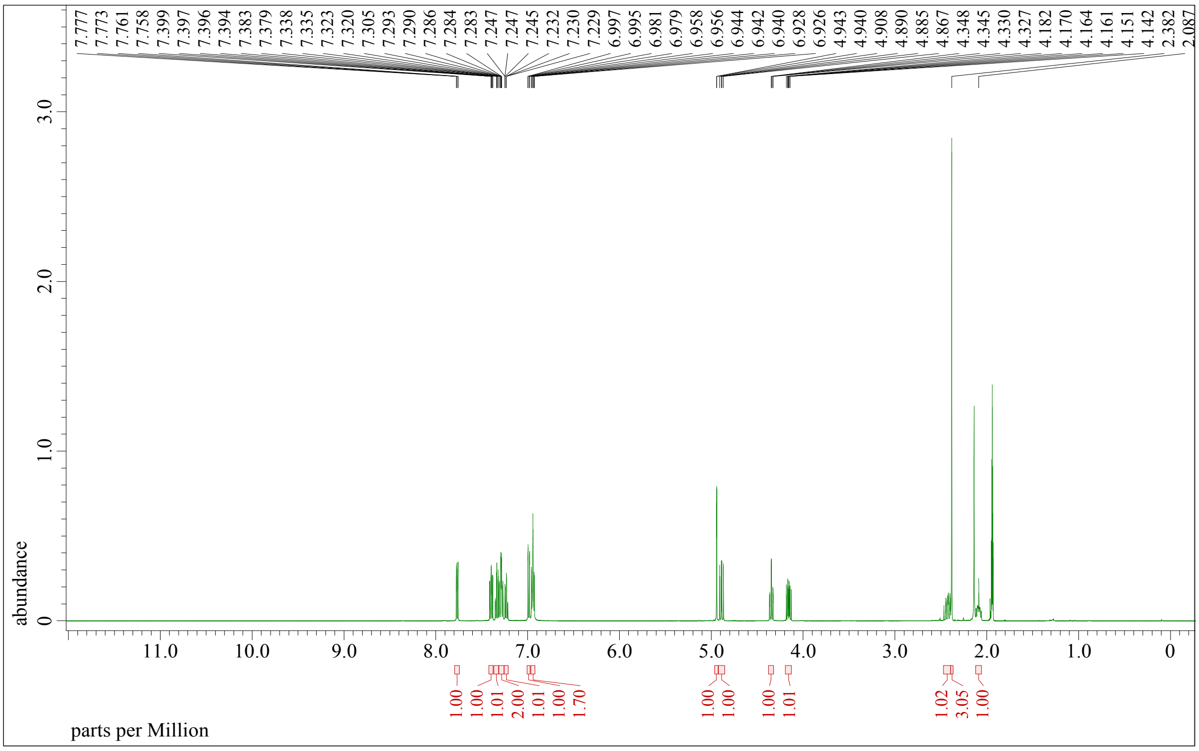

Figure S80. ^{1}H NMR spectrum of **9h** (500 MHz, MeCN-d_3)

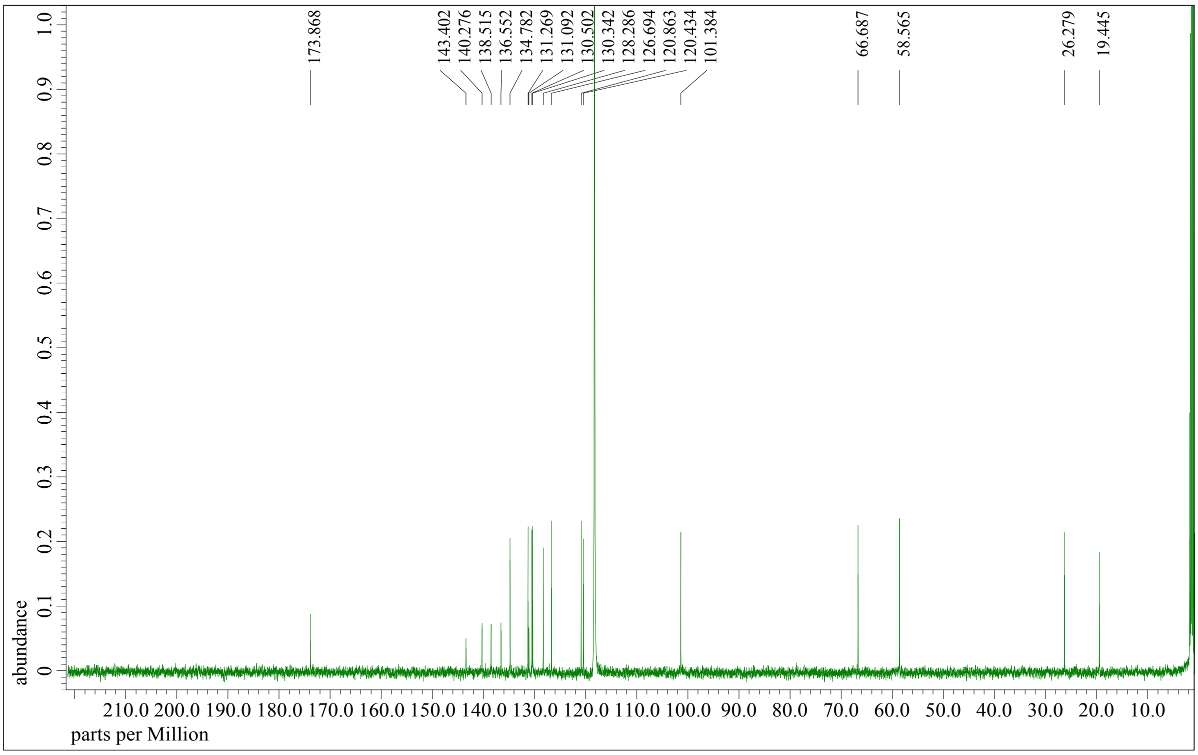

Figure S81. $^{13}C\{^{1}H\}$ NMR spectrum of **9h** (126 MHz, MeCN-d_3)

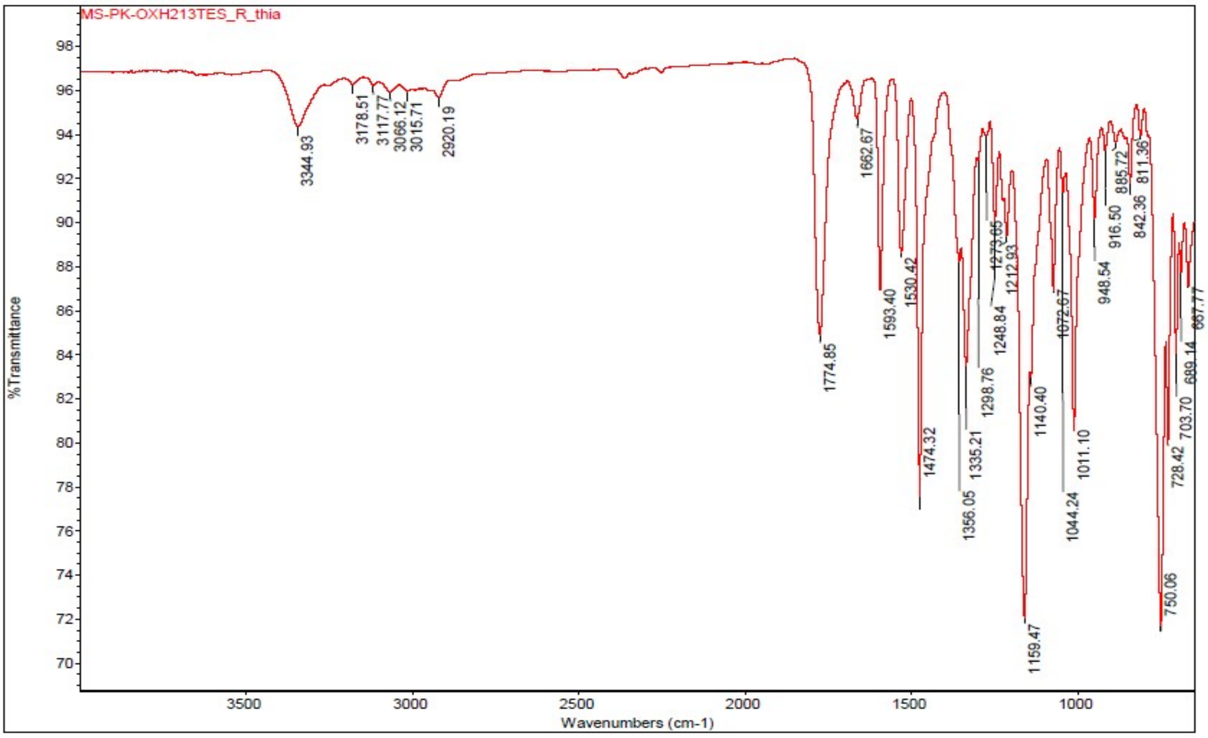

Figure S82. IR spectrum of **9h**

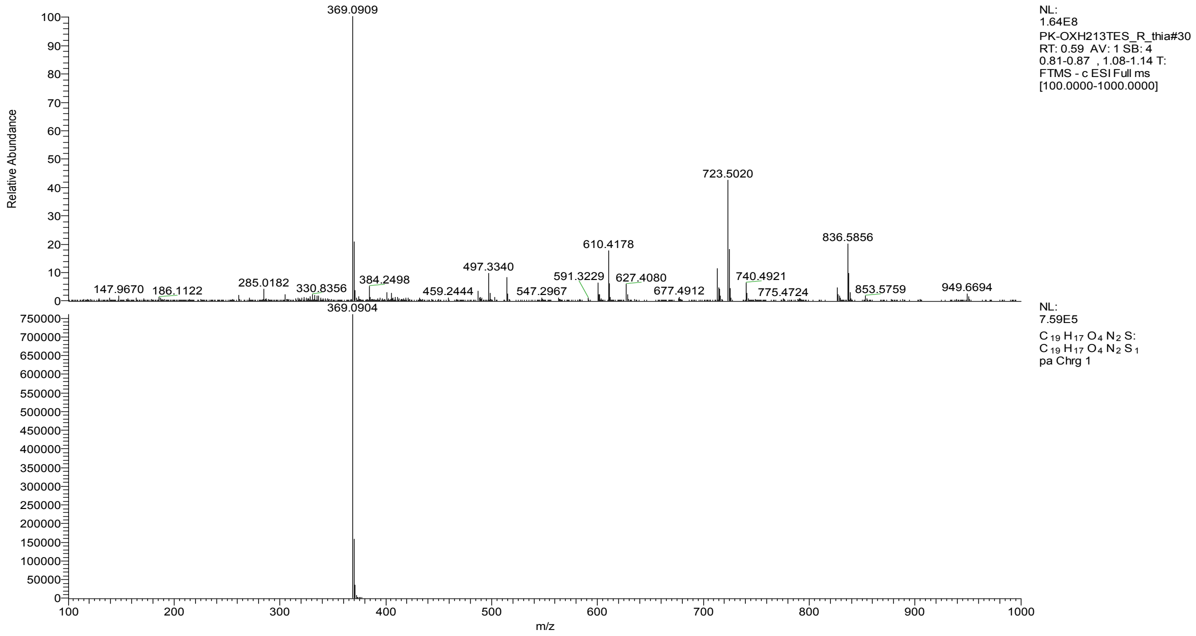

Figure S83. HRMS spectrum of **9h**

(-)-(*S*)-3-(4-(2-bromophenyl)-1,1-dioxobenzo[*f*][1,2,5]thiadiazepin-2(5*H*)-yl)dihydrofuran-2(3*H*)-one 9j

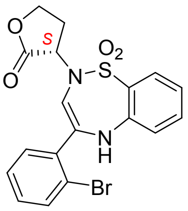

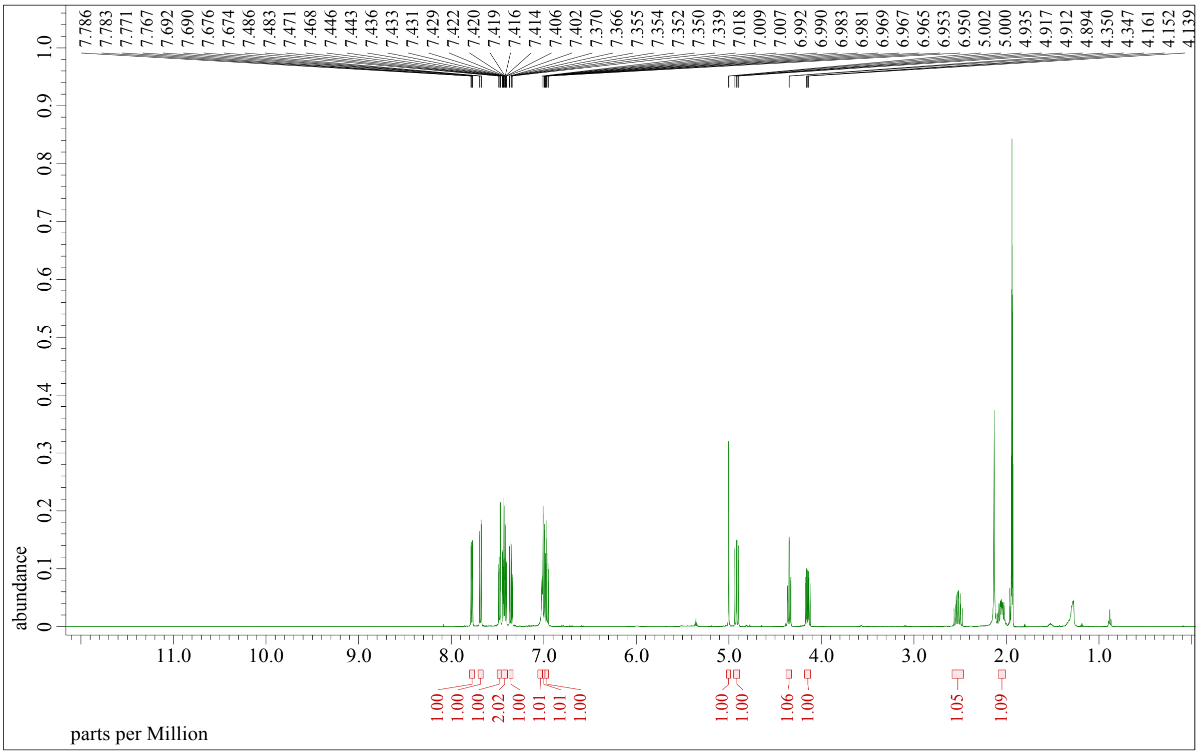

Figure S84. ^{1}H NMR spectrum of **9j** (500 MHz, MeCN-d_3)

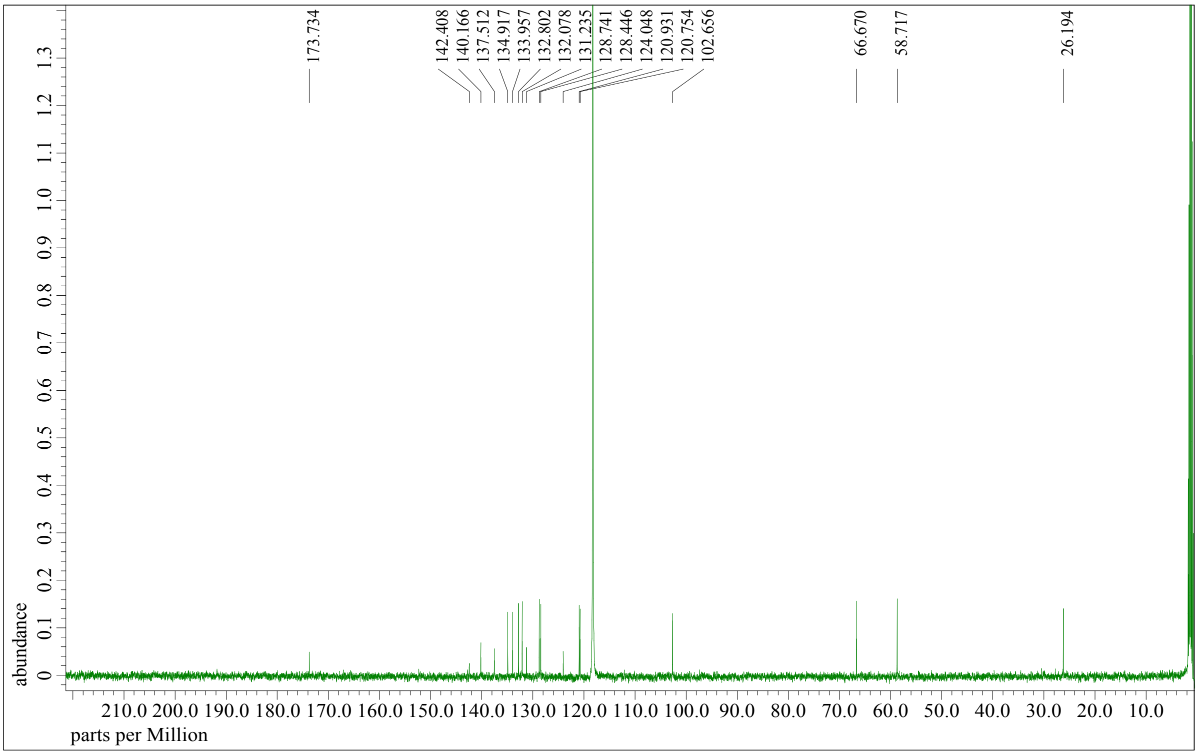

Figure S85. $^{13}C\{^{1}H\}$ NMR spectrum of **9j** (126 MHz, MeCN-d_3)

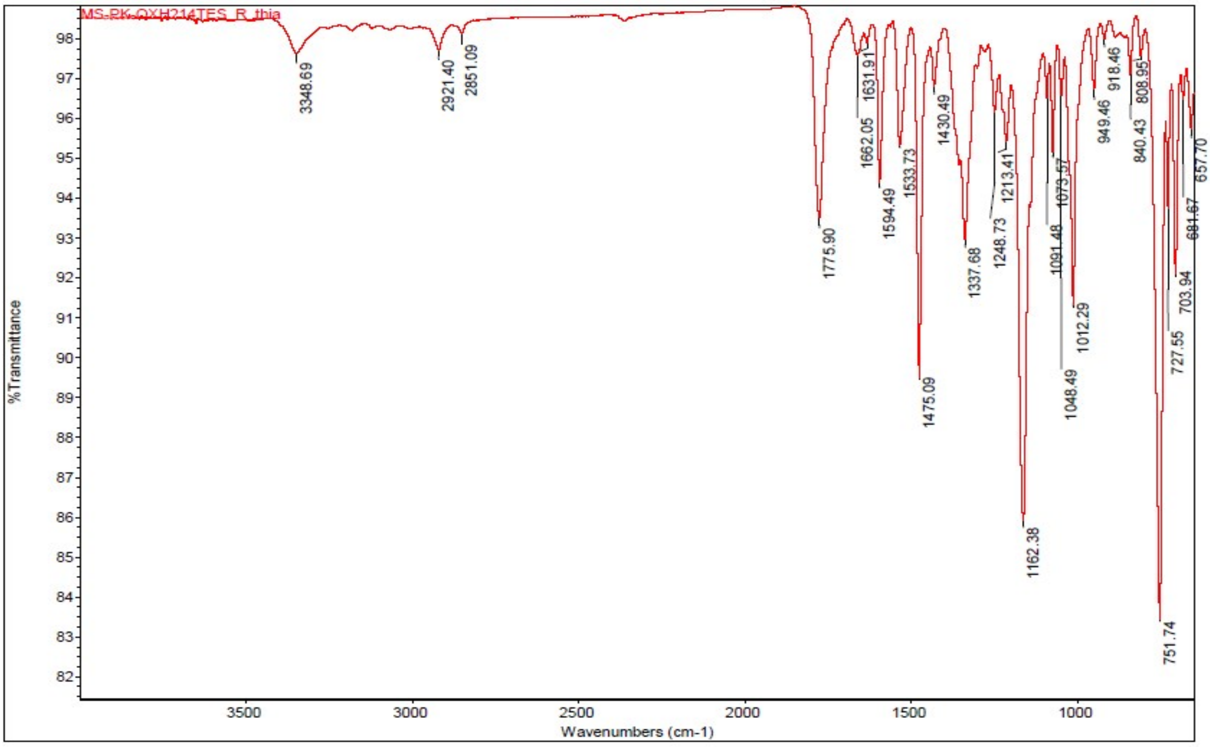

Figure S86. IR spectrum of **9j**

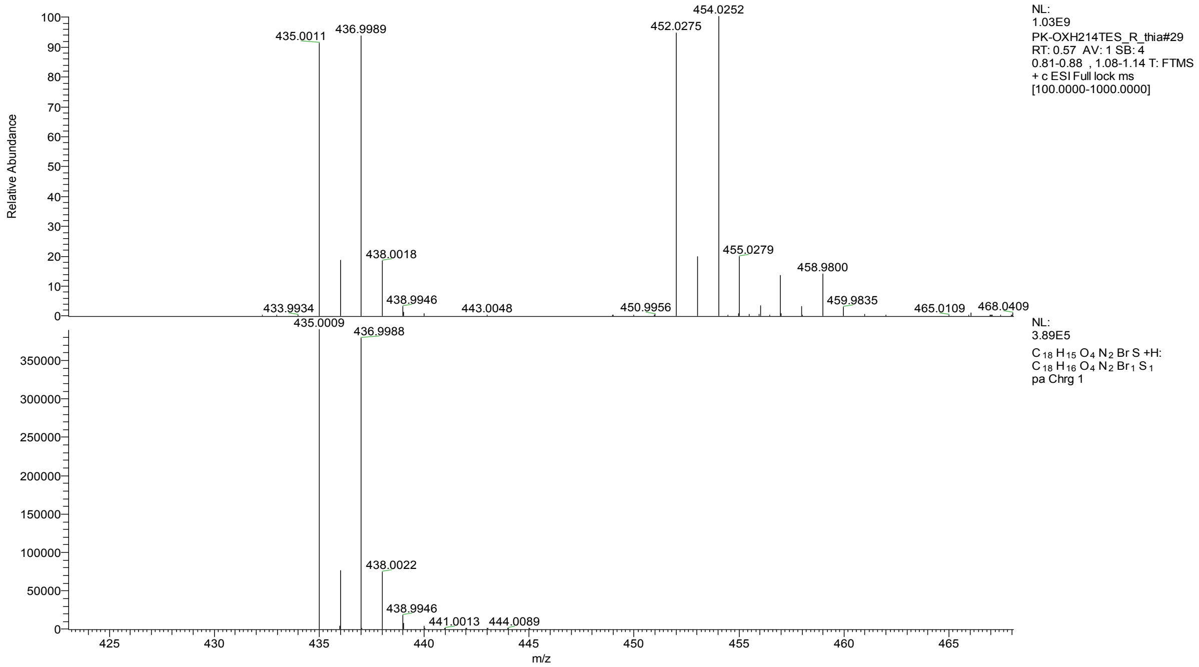

Figure 87. HRMS spectrum of **9j**. Note: ions 452 and 454 belongs to the ammonium adduct originating from mobile phase.

(+)-(*S*)-((2-aminophenyl)sulfonyl)-*L*-homoserine 10r

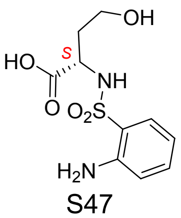

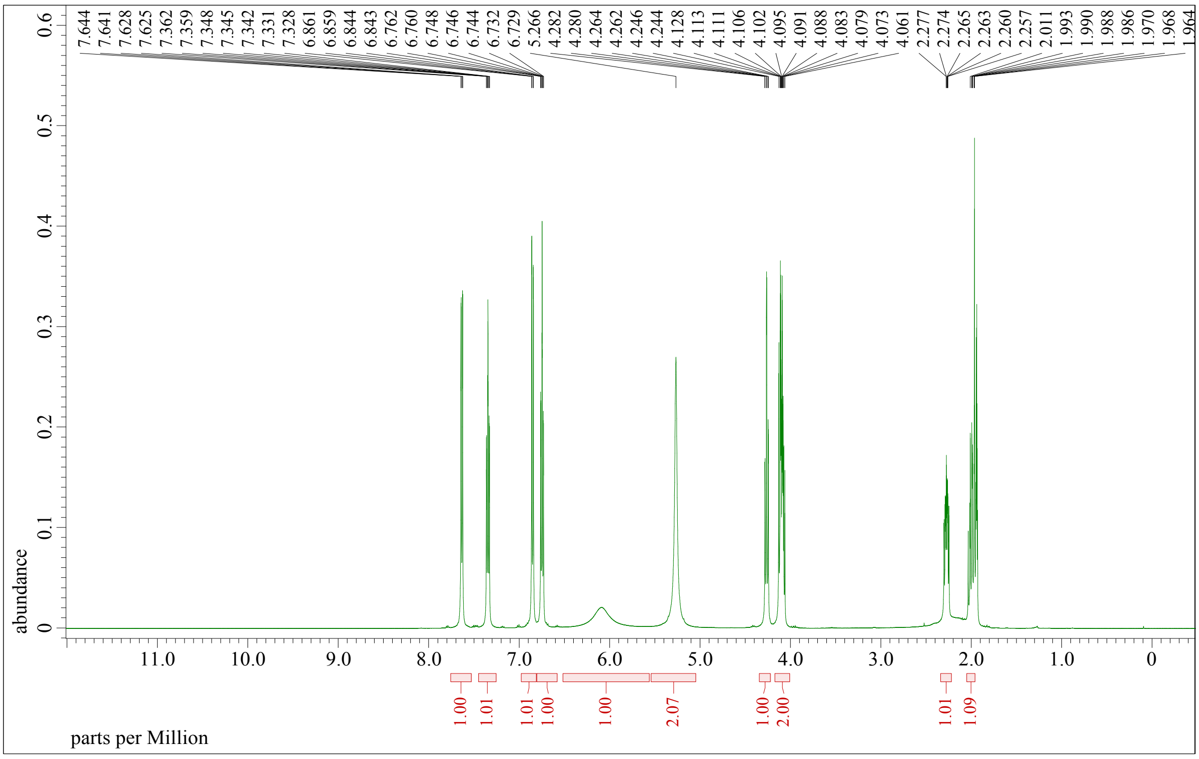

Figure S88. ^{1}H NMR spectrum of **10r** (500 MHz, MeCN-d_3)

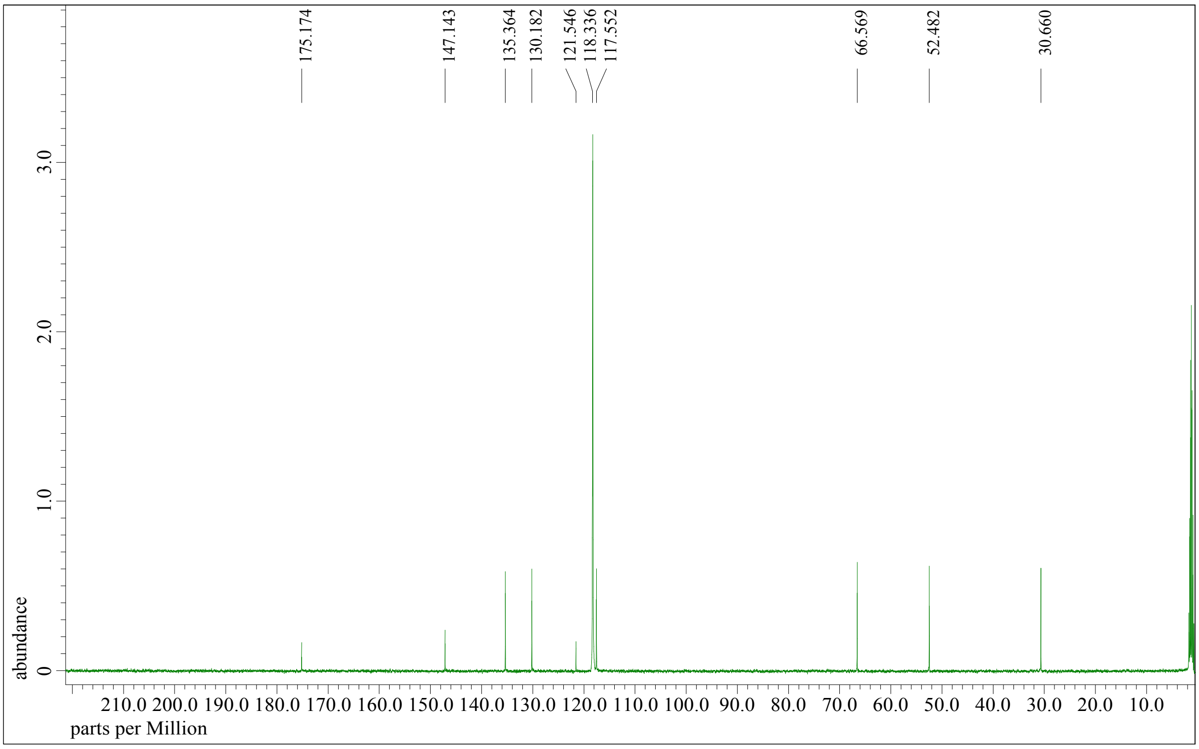

Figure S89. $^{13}C\{^{1}H\}$ NMR spectrum of **10r** (126 MHz, MeCN-d_3)

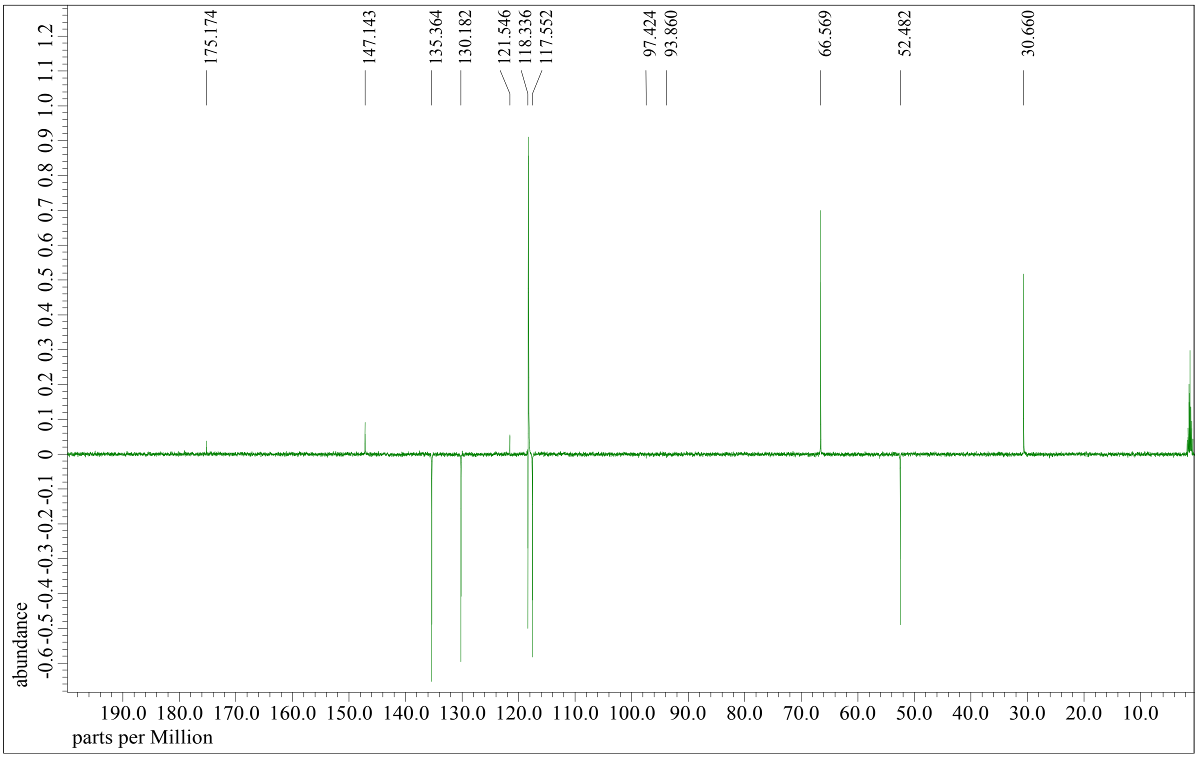

Figure S90. ^{13}C APT NMR spectrum of **10r** (126 MHz, MeCN-d_3)

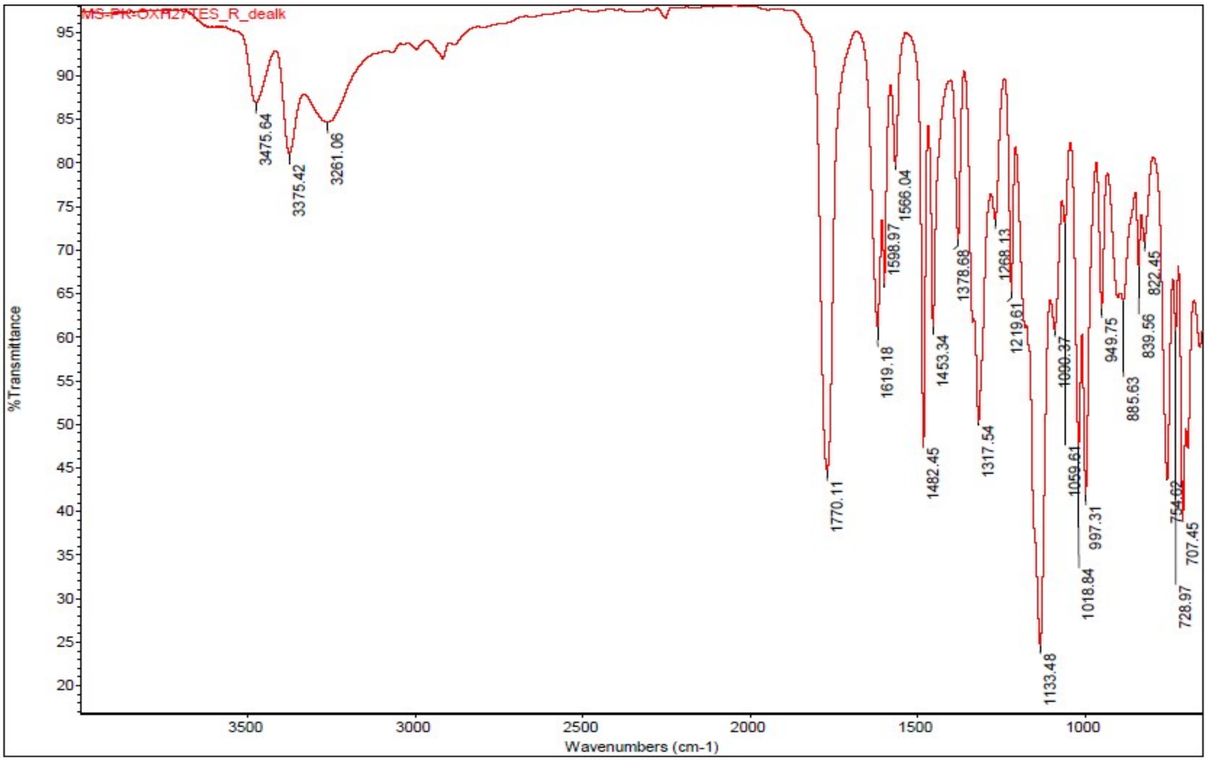

Figure S91. IR spectrum of **10r**

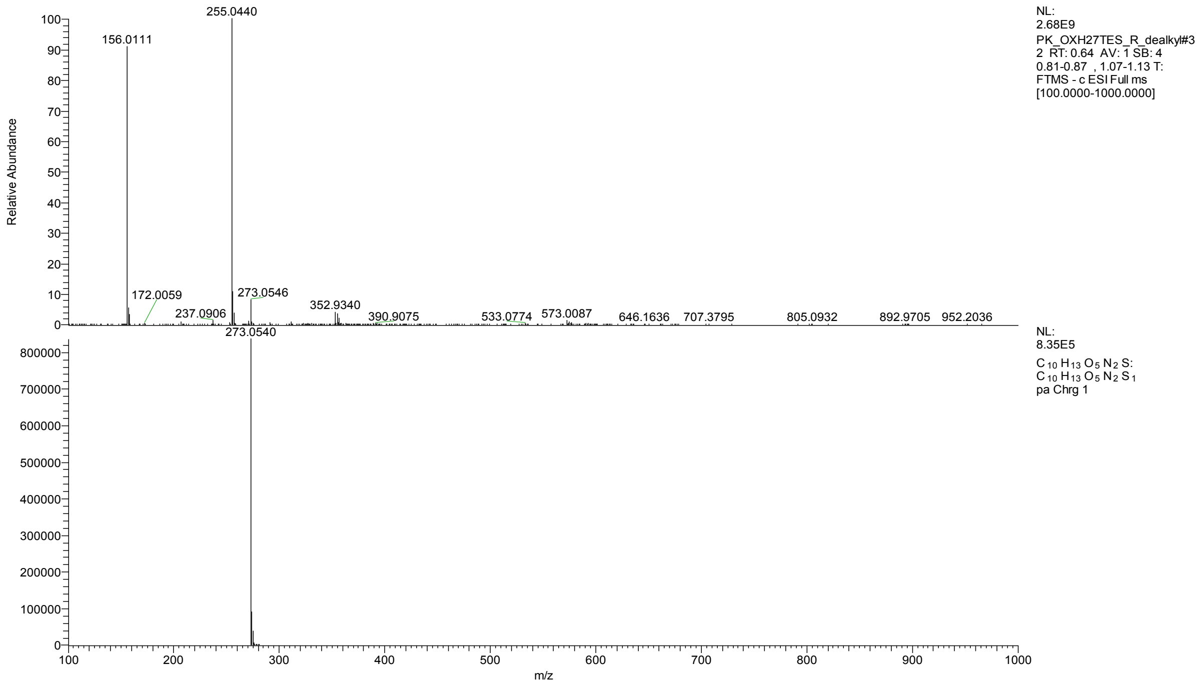

Figure S92. HRMS spectrum of **10r**

(-)-(*S*)-*N*-(4-methoxyphenethyl)-2-nitro-*N*-(2-oxotetrahydrofuran-3-yl)benzenesulfonamide 11n

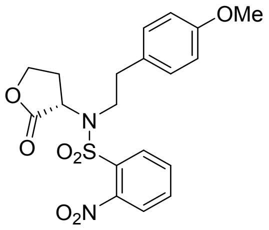

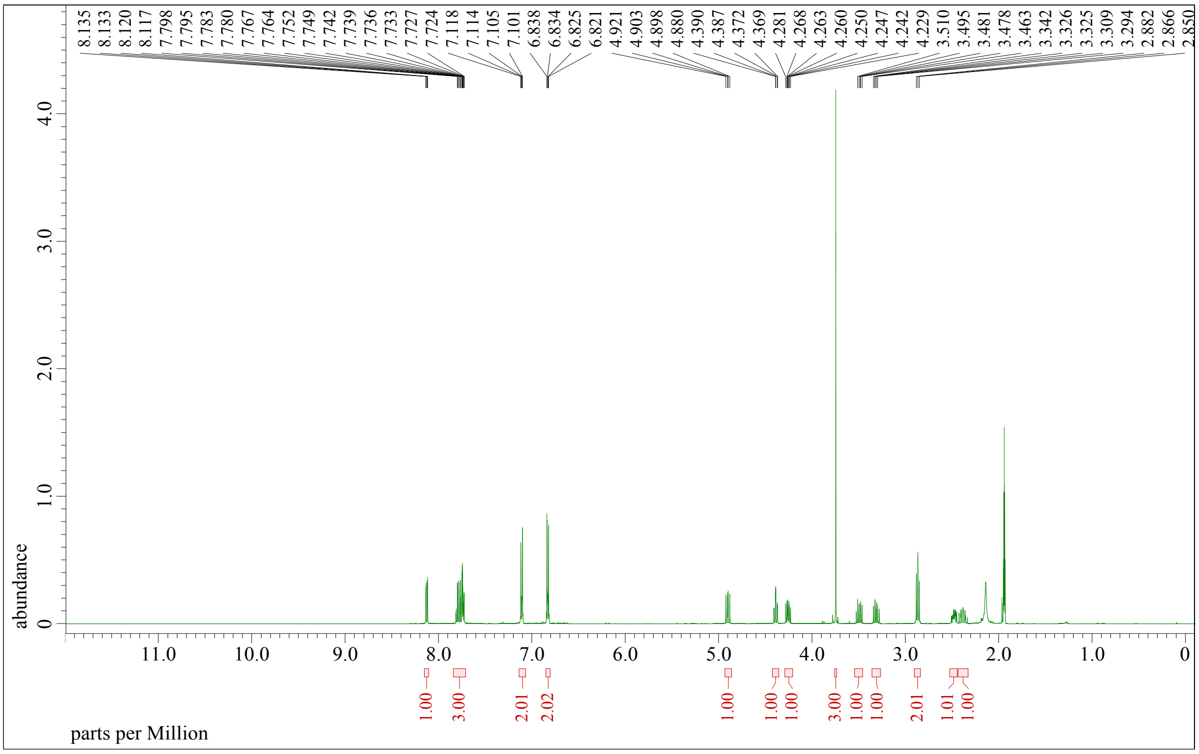

Figure S93. ^{1}H NMR spectrum of **11n** (500 MHz, MeCN-d_3)

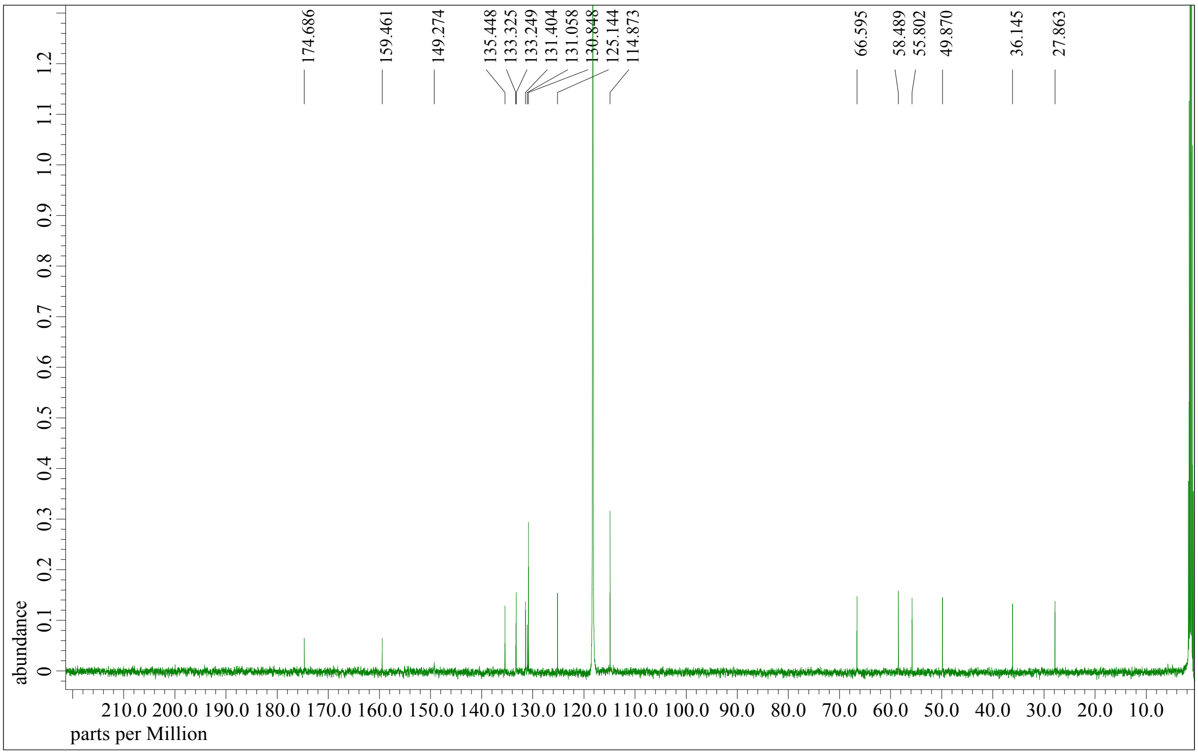

Figure S94. $^{13}C\{^1H\}$ NMR spectrum of **11n** (126 MHz, MeCN-d_3)

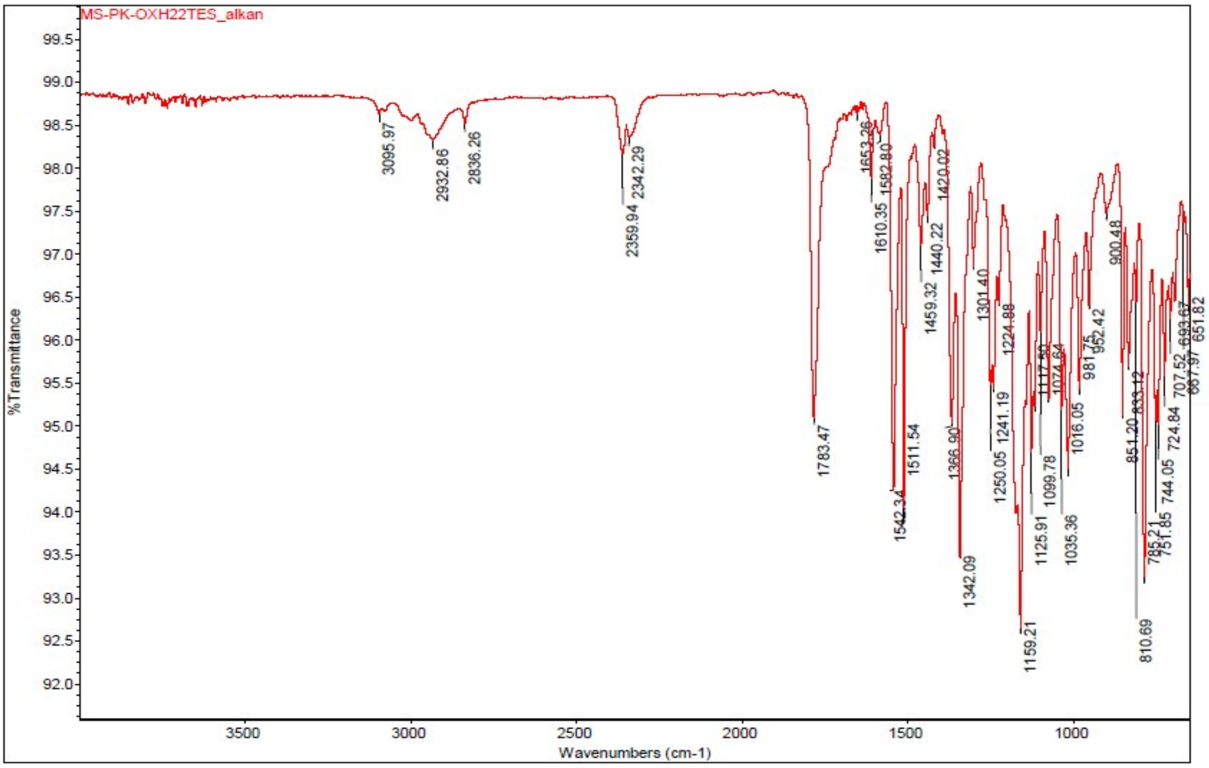

Figure S95. IR spectrum of **11n**

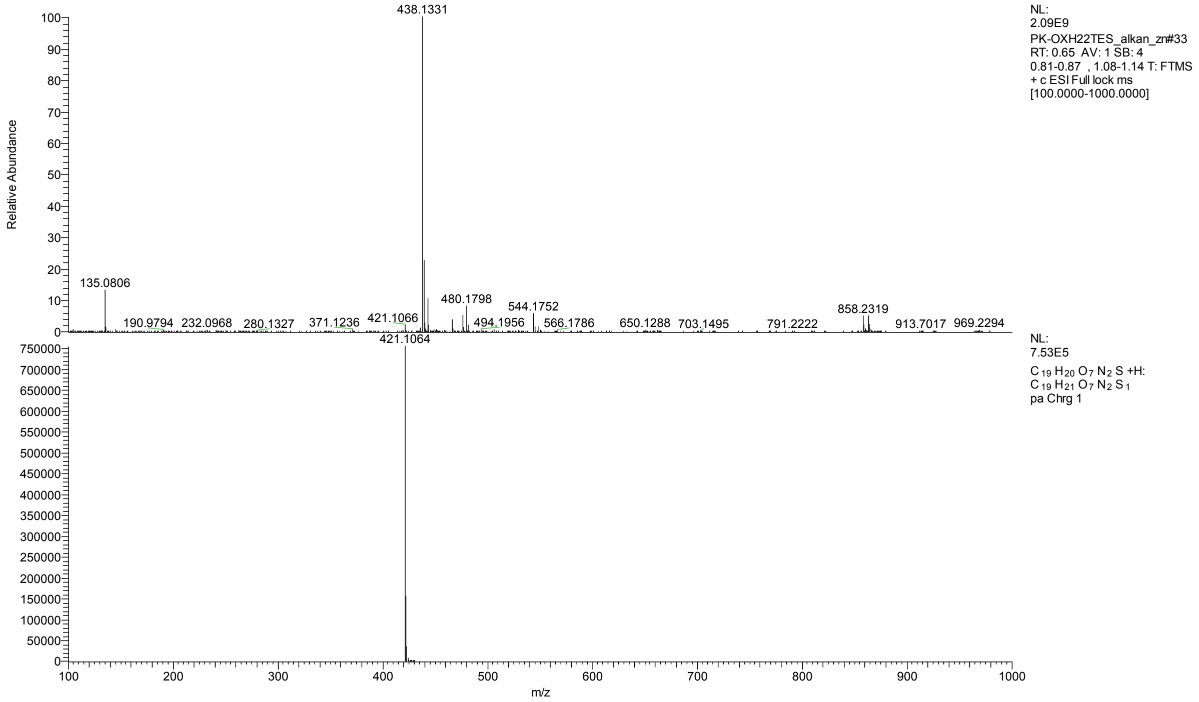

Figure S96. HRMS spectrum of **11n**. Note: ion 438 belongs to the ammonium adduct originating from mobile phase.
